# 3-Chloropropylbis(catecholato)silicate as a Bifunctional Reagent for the One-Pot Synthesis of Tetrahydroquinolines from *o*-Bromosulfonamides

**DOI:** 10.1021/acs.joc.3c02267

**Published:** 2024-02-27

**Authors:** Noah Brodsky, Nidheesh Phadnis, Mohamed Ibrahim, Isabel M. Andino, Inés Blanc Giro, John A. Milligan

**Affiliations:** Department of Biological and Chemical Sciences, College of Life Sciences, Thomas Jefferson University, 4201 Henry Avenue, Philadelphia, Pennsylvania 19144, United States

## Abstract

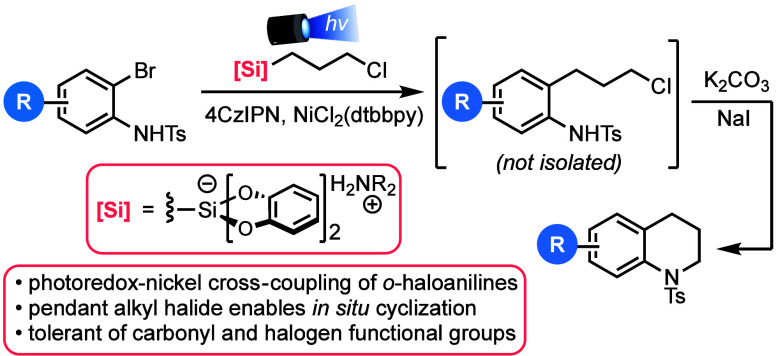

Bis(catecholato)silicate salts are easily accessible reagents that can be used to install alkyl fragments through photoredox-enabled cross-coupling. These reagents can incorporate various functional groups including pendant alkyl halides. A halogenated organosilicate reagent was leveraged to develop a one-pot synthesis of tetrahydroquinolines from *o*-bromosulfonamides, where the bifunctional reagent participates in a nickel/photoredox cross-coupling followed by intramolecular nucleophilic substitution. The functional group tolerance of this cross-coupling strategy allowed for the preparation of a series of substituted tetrahydroquinolines.

Tetrahydroquinolines are among the most common heterocycles found in medicinally useful molecules. Substantial effort has therefore been devoted to developing methods for their synthesis.^1^ Of the many possible retrosynthetic bond disconnections to attain the tetrahydroquinoline scaffold, the installation of a saturated three-carbon unit onto readily attained *o*-halogenated anilines is particularly attractive. This constitutes a retrosynthetic logic akin to the Larock indole synthesis, where *o*-halogenated anilines and alkynes are joined to make indoles.^2^ In fact, the Larock group themselves demonstrated in 1998 that alkene-tethered aryl sulfonamides can be used to prepare tetrahydroquinolines through a palladium-mediated intramolecular ring closure.^3^ Other groups have since developed related palladium-catalyzed tetrahydroquinoline formations from *o-*halogenated anilines containing a tethered alkene,^4^ an *N-*cyclopropyl substituent,^5^ or appended (*in situ* derived) alkyl boronate.^6^ Intramolecular C–N bond formation through cross-coupling with tethered amines has also been demonstrated.^7^ More recent work has built upon these precedents by developing direct approaches for the synthesis of tetrahydroquinolines from anilines and readily obtained reactants such as allylphenols^8^ or 1,3-diols^9^ (Scheme 1).

**Scheme 1:**
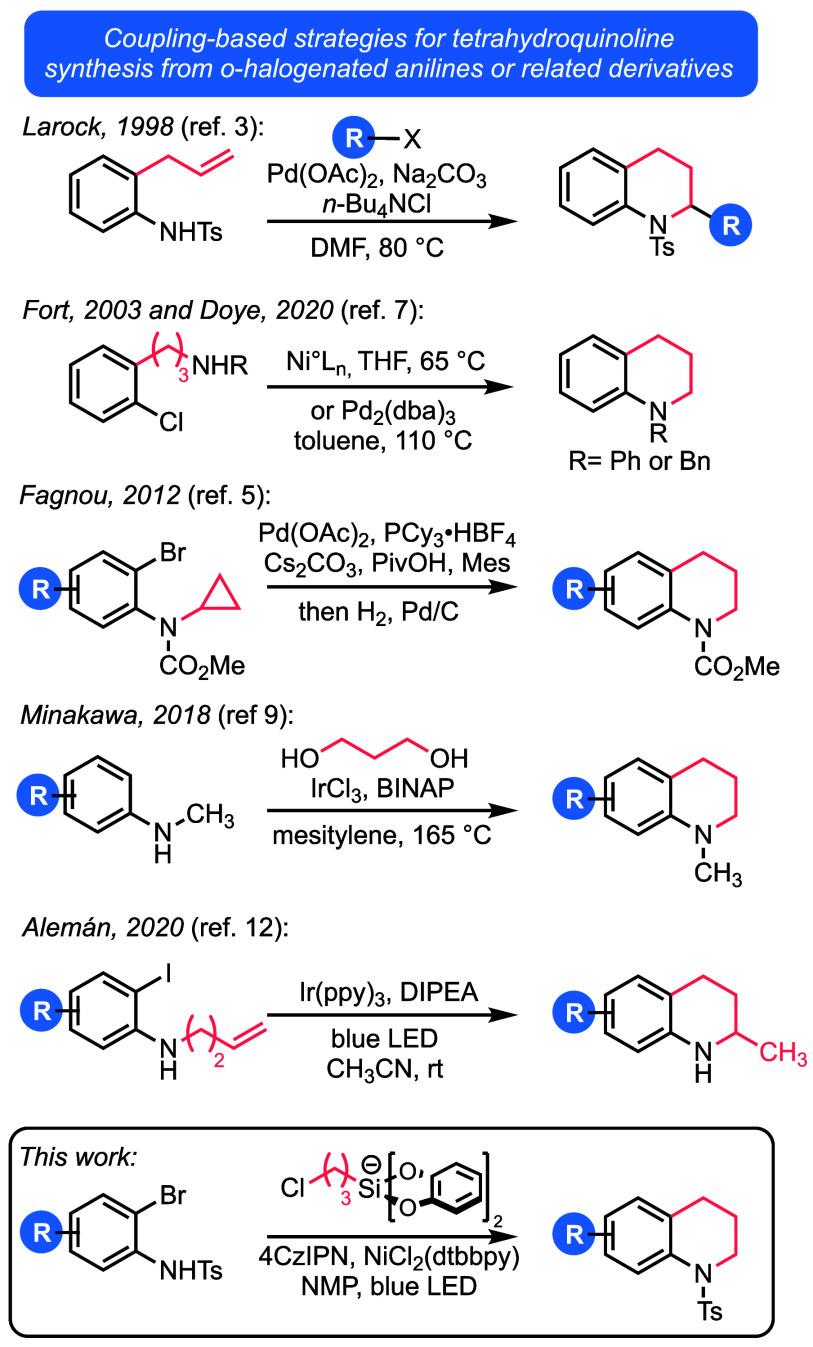
Coupling-Based Approaches to Tetrahydroquinolines through Reactions of Halogenated Anilines and Related Derivatives

A general limitation of most of these methods is the need to prepare pre-alkylated aniline precursors. Although more recent strategies^8,9^ obviate this necessity, the use of elevated temperatures or otherwise harsh conditions to accomplish the cyclization step can limit functional group compatibility in the preparation of tetrahydroquinolines. In contrast, photoredox-based cross-coupling methods have allowed chemists to reimagine classical cross-coupling approaches, including those in the service of heterocycle formation, in a way that is much more functional group tolerant.^10^ For example, photoredox catalysis has been used to prepare indolines from *o*-iodoanilines and functionalized alkenes.^11^ Although photoredox catalysis has been used to prepare tetrahydroquinolines through intramolecular cyclization of homoallylic anilines (Scheme 1),^12^ we are unaware of such a photoredox-based process for the direct installation of the saturated three-carbon unit that is needed for tetrahydroquinoline synthesis from *o*-halogenated anilines.

As part of our efforts to develop reagents for photoredox-enabled carbocycle and heterocycle synthesis,^13^ we became intrigued by the possibility of developing a bifunctional reagent^14^ capable of sequential, one-pot cross-coupling and nucleophilic substitution reactions. Bis(catecholato)silicate salts are well suited for such a purpose.^13,15^ These reagents, which are easily prepared from inexpensive trialkoxysilanes, have low oxidation potentials (approximately +0.75 V vs SCE) and can readily furnish primary alkyl radical equivalents under photoredox catalysis. Importantly, these organosilicates can be adorned with a variety of pendant functional groups, including halogens. Thus, these organosilicate salts have great potential as bifunctional reagents: the organosilicate and halide ends can operate independently of one another in radical and polar reactions, respectively (Scheme 2). This feature of the reagents has been leveraged in the past for pyrrolidine formation and the synthesis of several types of substituted cyclopropanes using a net-neutral radical/polar crossover mechanism.^13^

**Scheme 2:**
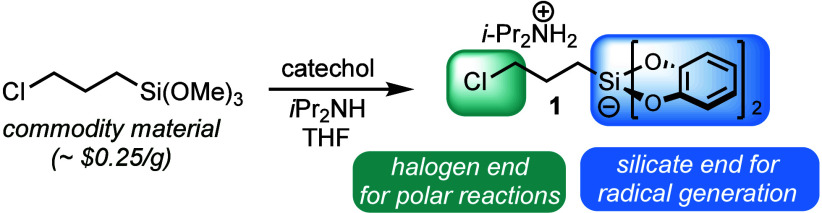
3-Chloropropyl (Bis)catecholatosilicate as a Bifunctional Reagent

Given these advantageous properties of organosilicates, we sought to apply 3-chloropropyl bis(catecholato)silicate **1** as a bifunctional reagent for tetrahydroquinoline synthesis. Reagent **1** (and other cation analogues thereof) has been shown to be competent in photoredox-mediated nickel cross-coupling^16^ and Giese addition reactions,^17^ and its brominated analogue has been used for pyrrolidine formation through nickel-free radical/polar crossover annulation reactions.^13b^ However, its use for the installation of a propylene unit through sequential cross-coupling and nucleophilic ring closure has yet to be explored. Sulfonamides containing an *o*-bromo substituent were envisioned to be ideal substrates to apply this concept, as the sulfonamide is expected to be an effective spectator for the cross-coupling step, which can be easily deprotonated to serve as a nucleophile in the subsequent ring closure. Therefore, we set out to apply the bifunctional reagent **1** in the synthesis of tetrahydroquinolines.

Our initial investigations focused on the identification of a suitable protocol for tetrahydroquinoline synthesis (Table 1; more extensive screening experiments are detailed in the Supporting Information). The reaction conditions were adapted from previously published organosilicate cross-coupling reactions, which clearly show that reagents such as **1** are only effective in polar aprotic solvents.^15^ In our initial screening, NMP, DMF, and DMA were similarly effective, with DMSO being less effective. There is some level of restriction as to the nature of the photocatalyst used, as the photocatalyst must be able to both oxidize the organosilicate reagent to furnish a radical and reduce the nickel halide intermediate to turn over the catalytic cycle.^15^ While standard iridium- and ruthenium-based catalysts are also viable for the reaction, we opted to use the easily prepared organic photocatalyst 4CzIPN.^18^ A broad screening of nickel ligands was not conducted due to the substantial number of references that use bipyridyl ligands for this type of cross-coupling. However, we found that using pre-complexed nickel (as opposed to adding NiCl_2_·dme and ligand separately to the reaction) improved the yield of product obtained.

**Table 1:**
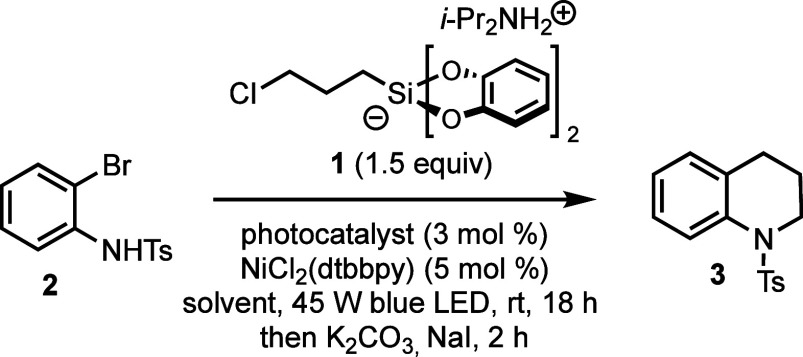
Variation of Reaction Parameters

solvent	photocatalyst	yield (%)^a^
NMP	4CzIPN	97 (71)
NMP	[Ir]^b^	84
NMP	[Ru(bpy)_3_](PF_6_)_2_	90
NMP	[Ru(bpy)_3_](PF_6_)_2_	56^c^
NMP	[Ru(bpy)_3_](PF_6_)_2_	54^d^
NMP	eosin Y	0
DMF	4CzIPN	85
DMA	4CzIPN	67
DMSO	4CzIPN	28

a Yields determined by HPLC using caffiene as an internal standard. Isolated yield in parentheses.

b [Ir] = [Ir{dF(CF_3_)_2_ppy]_2_(bpy)PF_6_.

c NiCl_2_·dme and dtbbpy added to the flask without pre-formation of ligated complex.

d Organosilicate loading reduced to 1.1 equiv.

The selected cross-coupling conditions were applied to sulfonamide **2**. After the mixture was stirred under blue LED irradiation for 18 h, LC-MS analysis of a crude reaction aliquot revealed a 1:1.3 ratio of tetrahydroquinoline **3** and the uncyclized alkyl chloride precursor, **3′** (Scheme 3). We found that simply treating the reaction mixture with K_2_CO_3_ (3 equiv) and NaI (1 equiv) without any workup facilitated clean conversion to **3** within 2 h. Lower loadings of these salts could be used, but given their inexpensive nature, we found that using an excess of the reagents gave consistently fast ring closure on a variety of substrates. However, we found that the one-pot sequence must be performed in two separate steps. Addition of NaI to the initial photoredox cross-coupling induces decomposition, presumably through the undesired redox chemistry of iodide.

**Scheme 3:**
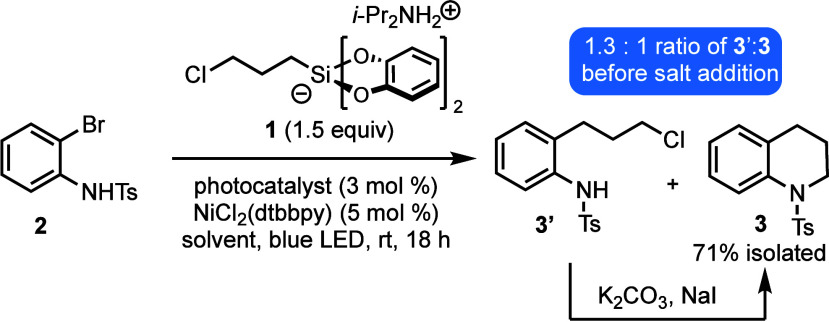
Description of the One-Pot Cross-Coupling/Cyclization Approach

The feasibility of the method was tested on a series of *o*-bromoaniline precursors (Scheme 4). As has typically been seen with other reactions of bis(catecholato)silicates, the reaction is tolerant of ketones, esters, and amides (**4**–**7**), including those with a free N–H group (**6**). Changing the position of the ester to the meta position (**8**) was also well tolerated. An aryl acetate derivative (**9**), despite having an activated methylene position, was also successful. Halogenated tetrahydroquinolines are also accessible through this approach. The haloselectivity of cross-couplings with bis(catecholato)silicate reagents^19^ is highlighted through the successful synthesis of **12**, which was obtained through both the *ortho*-iodo and *ortho*-bromo precursor.

**Scheme 4:**
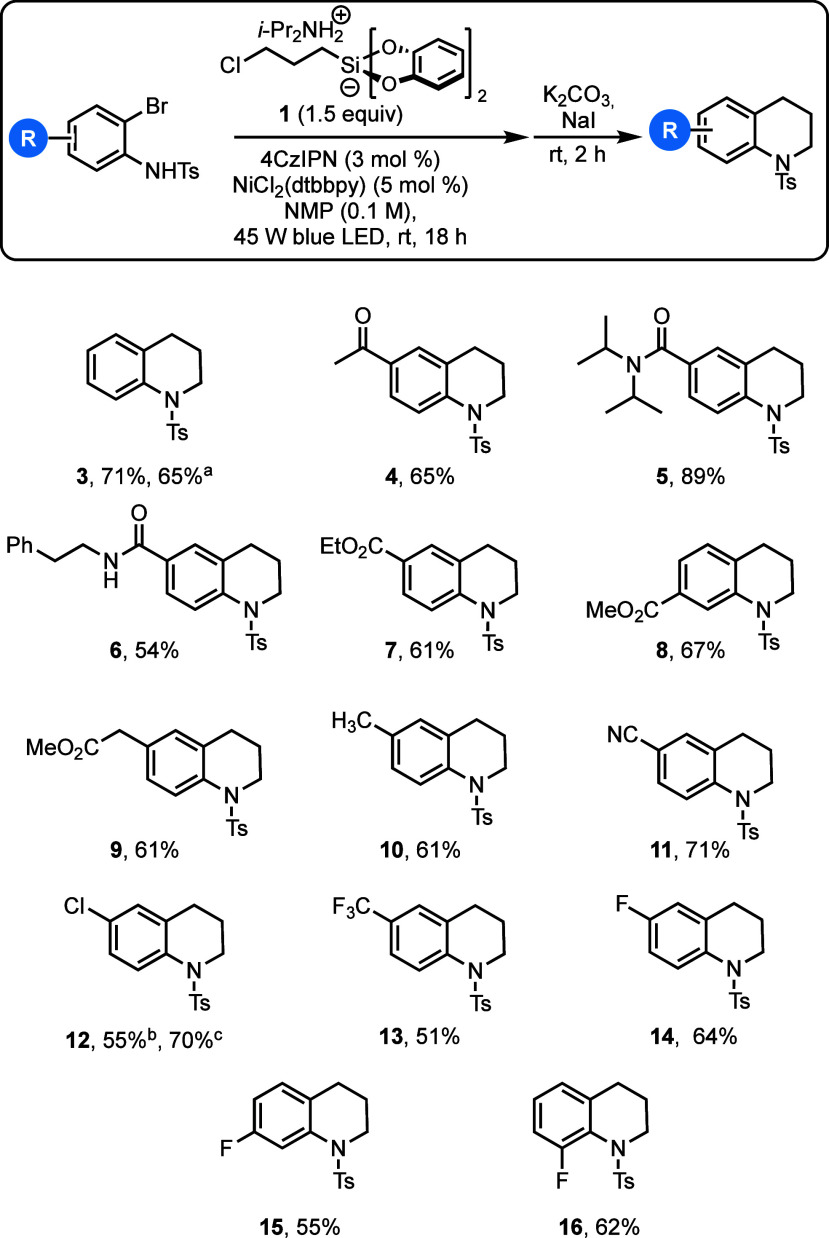
Scope of Tolerable Substrates in the Reaction Reaction conducted on a 1 g scale. *N*-(2-iodo-4-chlorophenyl)-*p*-toluenesulfonamide used as the starting material. *N*-(2-bromo-4-chlorophenyl)-*p*-toluenesulfonamide used as the starting material.

Unfortunately, substrates with redox-sensitive functional groups (such as nitro groups) were not successful in the reaction (see the Supporting Information for a full tabulation). The choice of photocatalyst for these reactions is inherently limited by the need to both efficiently generate primary alkyl radicals and turn over the catalytic cycle by reducing a halogenated nickel intermediate, which limits the ability to use photocatalysts with other redox windows that accommodate these more sensitive substrates. For this reason, this strategy for heterocycle formation could not be extended to the synthesis of chromanes due to the undesired redox activity of the requisite *o-*bromo phenol precursors.

Various other nitrogen protecting groups on the *o-*bromoaniline derivatives were tested. As expected, the sulfonamide group was the most successful in the reaction. Unsubstituted anilines did produce a trace amount of cross-coupling product, but substantial side product formation was observed. Mesylate- and trifluoroacetamide-substituted anilines were ineffective, giving only recovered starting material under the reaction conditions. Unfortunately, anilines with easily removed carbamate-based protecting groups such as Boc and Cbz were ineffective under cross-coupling conditions. In these cases, substantial amounts of protodehalogenated products were observed, suggesting that these groups facilitate oxidative addition of nickel but do not effectively promote cross-coupling with the photogenerated alkyl radical component. Although the N–S bond of the sulfonamide is not as easily cleaved as a carbamate protecting group, reductive, basic, and electrochemical methods for the deprotection of tetrahydroquinoline sulfonamide **3** and functionalized derivatives thereof have been reported.^20^

However, brominated acetanilide derivative **17** could be converted to tetrahydroquinoline **19** (Scheme 5). While cross-coupling to afford product **18** was successful, application of the one-pot protocol proved challenging because the excess diisopropyl ammonium and catechol-derived byproducts react with stronger bases such as NaH. We found it preferable to isolate the cross-coupled product **18** and then subject it to intramolecular ring closure with NaH to afford cyclized product **19**.

**Scheme 5:**
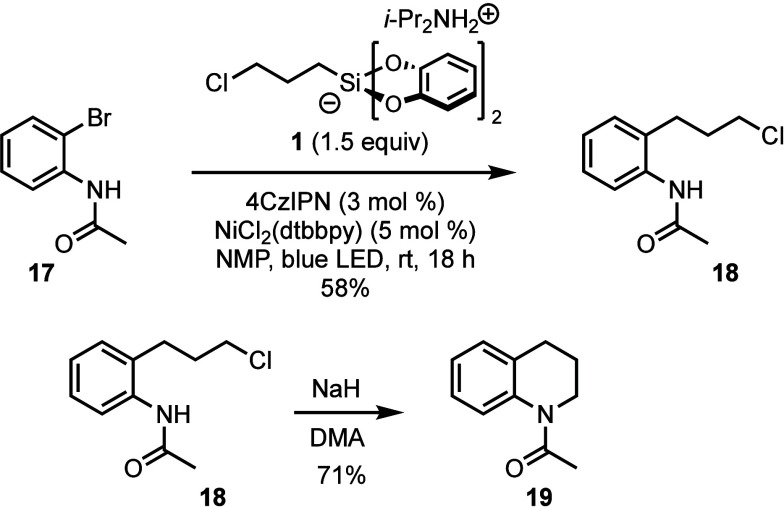
Cross-Coupling and Cyclization of an Acetanilide Derivative

Regardless of the nitrogen substituent employed, we propose that the reaction proceeds by the characteristic Ni^0^/Ni^I^/Ni^III^ redox cycle that is accepted for nickel-photoredox cross-couplings and has been computationally validated (Scheme 6).^21^ The photocatalyst (typically 4CzIPN) **A** becomes photoexcited upon irradiation (**B**). This excited state of the photocatalyst (P*/P– redox couple is +1.35 V for 4CzIPN) transfers an electron to oxidatively cleave the organosilicate reagent (oxidation potential of **1** is +0.75 V). The resultant transient primary alkyl radical is captured by the ligated nickel center **D** to form Ni^I^ complex **E**. Oxidative addition of the aryl bromide gives Ni^III^ intermediate **F**, which gives Ni^I^ bromide **G** and the cross-coupled product upon reductive elimination. As noted above, K_2_CO_3_ is added to the reaction mixture after irradiation to complete nucleophilic ring closure to form the tetrahydroquinoline product.

**Scheme 6:**
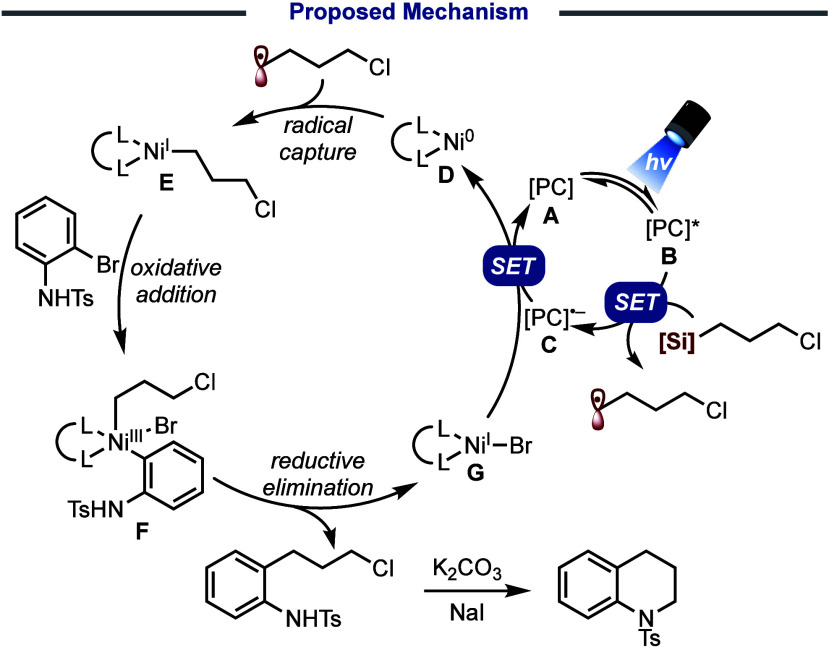
Proposed Mechanism

This application of chloro-tethered bis(catecholato)silicates in tetrahydroquinoline synthesis represents a novel strategy for deploying these inexpensive, readily obtained reagents. The specific approach developed herein also illustrates how readily attained *o-*bromo sulfonamides can be used as tetrahydroquinoline precursors through photoredox/nickel cross-coupling, which constitutes a milder alternative to other coupling-based approaches for tetrahydroquinoline synthesis. More broadly, we envision this family of bifunctional organosilicate reagents could be capable of other types of carbo- and heterocycle forming reactions, and investigations along these lines are ongoing in our laboratory.

## Experimental Section

### General Information

General experimental information and details on the preparation of the *o*-bromoaniline precursors are available in the Supporting Information.

### Experimental Procedures

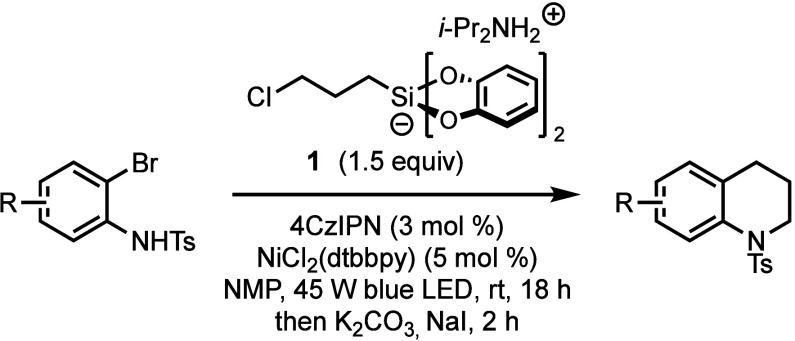



#### General Procedure for Cross-Coupling/Cyclization Sequences Using 3-Chloropropyl (Bis)catecholato Silicate **1**

A 2-dram vial with a cap containing a Teflon membrane was charged with the appropriate *o*-bromo sulfonamide (1.00 equiv), 3-chloropropylbis(catecholato) silicate **1** (1.50 equiv), 4CzIPN (3 mol %), and pre-formed NiCl_2_(dtbbpy) (5 mol %) in NMP (0.1 M). The vial was then capped and flushed with a N_2_ gas. The vial was irradiated with 2 blue PR-160 Kessil lamps arranged in a Kessil rig for 18 h. Without any workup, the reaction mixture was treated with NaI (1.00 equiv) and K_2_CO_3_ (3.00 equiv) and then stirred for 2–3 h without blue LED irradiation. The reaction mixture was then partitioned between 1 M NaOH (30 mL) and EtOAc (30 mL). The aqueous layer was extracted with additional EtOAc (30 mL). The combined organic layers were washed with water (50 mL) and brine (50 mL) and then dried and concentrated. Purification by chromatography on SiO_2_ (5–30% ethyl acetate in hexanes) afforded the desired tetrahydroquinoline products.

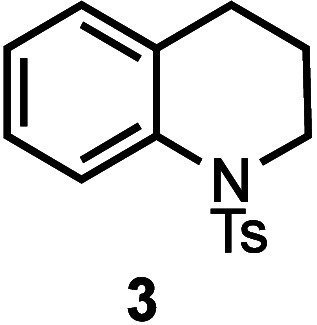



#### 1-(4-Methylbenzenesulfonyl)-1,2,3,4-tetrahydroquinoline **3**

Prepared according to General Procedure B from sulfonamide **2** (0.098 g, 0.30 mmol, 1.0 equiv), **1** (0.191 g, 0.45 mmol, 1.50 equiv), 4CzIPN (0.008 g, 3 mol %), and pre-formed NiCl_2_(dtbbpy) (0.006 mg, 5 mol %) in NMP (3 mL), followed by addition of NaI (0.045 g, 0.30 mmol, 1.00 equiv) and K_2_CO_3_ (0.124 g, 0.90 mmol, 3.00 equiv). Workup and purification by chromatography on SiO_2_ afforded **3** (0.061 g, 71%) as a colorless solid (mp 92–94 °C) . The spectroscopic properties of **3** are in accord with published data.^22^

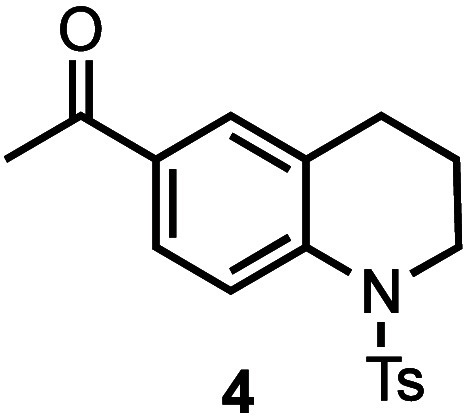



#### 6-Acetyl-1-(4-methylbenzenesulfonyl)-1,2,3,4-tetrahydroquinoline **4**

Prepared according to General Procedure B from sulfonamide **S4** (0.147 g, 0.40 mmol, 1.0 equiv), **1** (0.254 g, 0.60 mmol, 1.50 equiv), 4CzIPN (0.009 g, 3 mol %), and pre-formed NiCl_2_(dtbbpy) (0.008 g, 5 mol %) in NMP (4 mL), followed by addition of NaI (0.030 g, 0.20 mmol, 1.00 equiv) and K_2_CO_3_ (0.083 g, 0.60 mmol, 3.00 equiv). Workup and purification by chromatography on SiO_2_ afforded **4** (0.085 g, 65%) as a colorless oil: ^1^H NMR (400 MHz, CDCl_3_) δ 7.88 (d, 1 H, *J* = 8.4 Hz), 7.75–7.68 (m, 1 H), 7.63 (s, 1 H), 7.52 (d, 2 H, *J* = 8.0 Hz), 7.21 (d, 2 H, *J* = 8.0 Hz), 3.86–3.83 (m, 2 H), 2.61–2.51 (m, 2 H), 2.56 (s, 3 H), 2.38 (s, 3 H), 1.74–1.65 (m, 2 H); ^13^C{^1^H} NMR (100 MHz, CDCl_3_) δ 197.4, 144.0, 141.4, 136.4, 132.9, 129.8, 129.6, 129.4, 127.0, 123.3, 119.4, 46.8, 27.1, 26.5, 21.6, 21.5; IR (ATR) 3415 (w), 1677 (m), 1600 (m), 1354 (m), 1268 (m), 1162 (s), 1090 (w), 670 (m); HRMS (ESI+) calcd for C_18_H_20_NO_3_S [M + H] 330.1158, found 330.1157.

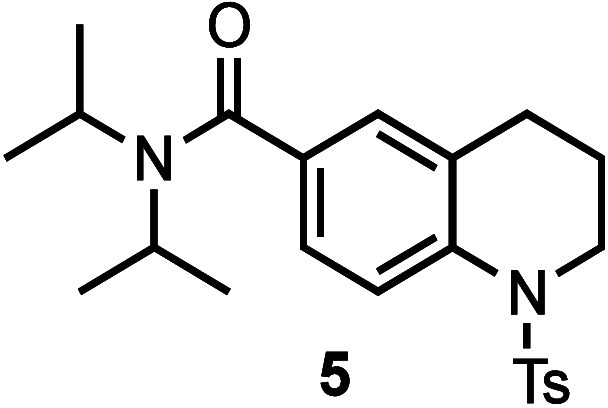



#### Diisopropyl 1-(4-Methylbenzenesulfonyl)-1,2,3,4-tetrahydroquinolin-6-carboxamide **5**

Prepared according to General Procedure B from sulfonamide **S5** (0.091 g, 0.20 mmol, 1.0 equiv), **1** (0.127 g, 0.30 mmol, 1.50 equiv), 4CzIPN (0.005 g, 3 mol %), and NiCl_2_(dtbbpy) (0.004 mg, 5 mol %), followed by addition of NaI (0.030 g, 0.20 mmol, 1.00 equiv) and K_2_CO_3_ (0.083 g, 0.60 mmol, 3.00 equiv). Purification by chromatography on SiO_2_ afforded **5** (0.074 g, 89%) as a colorless oil: ^1^H NMR (400 MHz, CDCl_3_) δ 7.81 (d, 1 H, *J* = 8.4 Hz), 7.54 (d, 2 H, *J* = 8.0 Hz), 7.22 (d, 2 H, *J* = 8.0 Hz), 7.12 (d, 1 H, *J* = 8.8 Hz), 7.04 (br s, 1 H), 3.85–3.77 (m, 2 H), 3.80–3.50 (br, 2 H), 2.54 (t, 2 H, *J* = 6.4 Hz), 2.41 (s, 3 H), 1.72–1.63 (m, 2 H), 1.64–0.99 (br, 12 H); ^13^C{^1^H} NMR (100 MHz, CDCl_3_) δ 170.6, 143.8, 137.4, 136.6, 134.9, 130.4, 129.7, 127.09, 127.06, 124.0, 123.7, 46.6, 26.7, 21.5, 21.4, 20.8; IR (ATR) 2964 (w), 1613 (m), 1442 (m), 1338 (s), 1161 (s), 1090 (w), 730 (w) cm^–1^; HRMS (ESI+) calcd for C_23_H_30_N_2_O_3_SNa [M + Na^+^] 437.1875, found 437.1869.

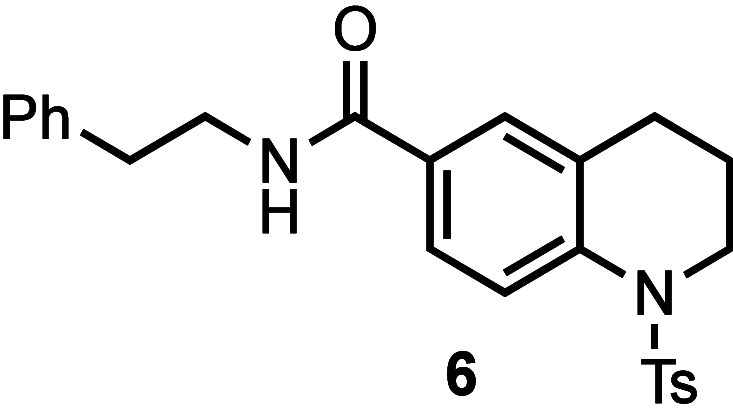



#### Phenethyl 1-(4-Methylbenzenesulfonyl)-1,2,3,4-tetrahydroquinolin-6-carboxamide **6**

Prepared according to General Procedure B from sulfonamide **S6** (0.118 g, 0.25 mmol, 1.0 equiv), **1** (0.159 g, 0.38 mmol, 1.50 equiv), 4CzIPN (0.006 g, 3 mol %), and pre-formed NiCl_2_(dtbbpy) (0.005 g, 5 mol %) in NMP (2.5 mL), followed by addition of NaI (0.038 g, 0.25 mmol, 1.00 equiv) and K_2_CO_3_ (0.104 g, 0.75 mmol, 3.00 equiv). Workup and purification by chromatography on SiO_2_ afforded **6** (0.059 g, 54%) as a colorless oil: ^1^H NMR (400 MHz, CDCl_3_) δ 7.84 (d, 1 H, *J* = 8.8 Hz), 7.54–7.47 (m, 3 H), 7.42–7.31 (m, 3 H), 7.31–7.18 (m, 5 H), 6.16 (br s, 1 H), 3.85–3.80 (m, 2 H), 3.76–3.65 (m, 2 H), 2.98–2.89 (m, 2 H), 2.54 (t, 2 H, *J* = 6.8 Hz), 2.40 (s, 3 H), 1.71–1.62 (m, 2 H); ^13^C{^1^H} NMR (100 MHz, CDCl_3_) δ 166.9, 143.9, 139.8, 138.9, 136.4, 130.4, 130.3, 129.9, 129.8, 128.83, 128.77, 128.75, 128.6, 127.2, 127.0, 126.6, 124.3, 123.9, 46.7, 41.4, 35.7, 26.9, 21.6, 21.3; IR (ATR) 3323 (w), 2927 (w), 1636 (m), 1541 (m), 1491 (s), 1341 (m), 1251 (m), 1162 (vs), 1090 (w), 699 (w) cm^–1^; HRMS (ESI+) calcd for C_25_H_26_N_2_O_3_SNa [M + Na^+^] 457.1562, found 457.1558.

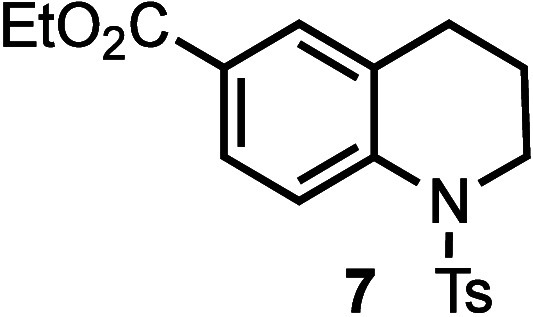



#### Ethyl 1-(4-Methylbenzenesulfonyl)-1,2,3,4-tetrahydroquinolin-6-carboxylate **7**

Prepared according to General Procedure B from sulfonamide **S7** (0.074 g, 0.20 mmol, 1.0 equiv), **1** (0.127 g, 0.30 mmol, 1.50 equiv), 4CzIPN (0.005 g, 3 mol %), and pre-formed NiCl_2_(dtbbpy) (0.004 g, 5 mol %) in NMP (2 mL), followed by addition of NaI (0.030 g, 0.20 mmol, 1.00 equiv) and K_2_CO_3_ (0.083 g, 0.60 mmol, 3.00 equiv). Workup and purification by chromatography on SiO_2_ afforded **7** (0.044 g, 61%) as a colorless oil: ^1^H NMR (400 MHz, CDCl_3_) δ 7.87 (d, 1 H, *J* = 8.8 Hz), 7.81 (dd, 1 H, *J* = 8.8 Hz, 2.0 Hz), 7.70–7.68 (m, 1 H), 7.51 (d, 2 H, *J* = 8.4 Hz), 7.20 (d, 2 H, *J* = 8.0 Hz), 4.34 (q, 2 H, *J* = 7.2 Hz), 3.86–3.81 (m, 2 H), 2.56 (t, 2 H, *J* = 6.8 Hz), 2.37 (s, 3 H), 1.74–1.66 (m, 2 H), 1.37 (t, 3 H, *J* = 7.2 Hz); ^13^C{^1^H} NMR (100 MHz, CDCl_3_) δ 166.3, 143.9, 141.0, 136.4, 130.7, 129.7, 129.6, 127.8, 127.0, 126.2, 123.5, 60.9, 46.8, 27.0, 21.6, 21.4, 14.4; IR (ATR) 2938 (w), 1711 (m), 1609 (m), 1493 (m), 1342 (m), 1161 (s), 1089 (m), 662 (m) cm^–1^; HRMS (ESI+) calcd for C_19_H_22_NO_4_S [M + H] 360.1270, found 360.1271.

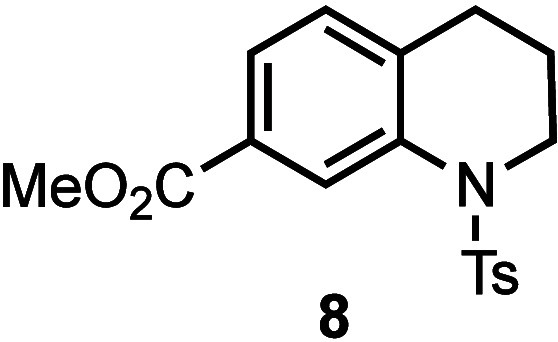



#### Methyl 1-(4-Methylbenzenesulfonyl)-1,2,3,4-tetrahydroquinolin-7-carboxylate **8**

Prepared according to General Procedure B from sulfonamide **S8** (0.154 g, 0.400 mmol, 1.00 equiv), **1** (0.254 g, 0.600 mmol, 1.50 equiv), 4CzIPN (0.009 g, 0.01 mmol, 3 mol %), and pre-formed NiCl_2_(dtbbpy) (0.008 g, 5 mol %) in NMP (4 mL), followed by addition of NaI (0.060 g, 0.24 mmol, 1.00 equiv) and K_2_CO_3_ (0.099 g, 0.72 mmol, 3.00 equiv). Workup and purification by chromatography on SiO_2_ afforded ester **8** (0.092 g, 67%) as a colorless oil: ^1^H NMR (400 MHz, CDCl_3_) δ 8.45 (d, 1 H, *J* = 1.6 Hz), 7.73 (dd, 1 H, *J* = 8.0 Hz, 2.0 Hz), 7.49 (d, 2 H, *J* = 8.4 Hz), 7.20 (d, 2 H, *J* = 8.4 Hz), 7.07 (d, 1 H, *J* = 8.0 Hz), 3.91 (s, 3 H), 3.84–3.77 (m, 2 H), 2.50 (t, 2 H, *J* = 6.8 Hz), 2.38 (s, 3 H), 1.70–1.62 (m, 2 H); ^13^C{^1^H} NMR (100 MHz, CDCl_3_) δ 166.8, 143.8, 137.0, 136.5, 135.7, 129.7, 129.2, 128.7, 127.2, 125.9, 125.8, 52.2, 46.3, 26.9, 21.6, 21.3; IR (ATR) 2952 (w), 1718 (m), 1597 (w), 1340 (m), 1256 (m), 1160 (s), 1090 (w), 660 (m) cm^–1^; HRMS (ESI+) calcd for C_18_H_19_NO_4_SNa [M + Na^+^] 368.0932, found 368.0930.

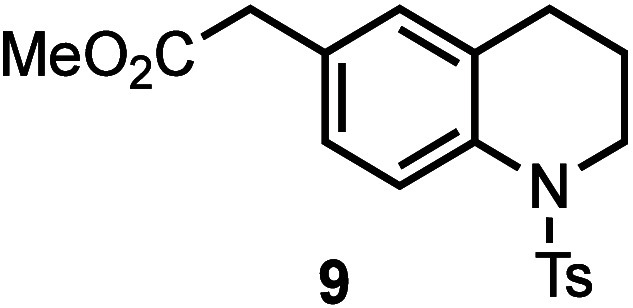



#### Methyl 1-(4-Methylbenzenesulfonyl)-1,2,3,4-tetrahydro-6-quinolinyl acetate **9**

Prepared according General Procedure B from **S9** (0.066 g, 0.17 mmol, 1.0 equiv), **1** (0.105 g, 0.25 mmol, 1.50 equiv), 4CzIPN (0.004 g, 3 mol %), and NiCl_2_(dtbbpy) (0.003 g, 5 mol %) in NMP (1.7 mL), followed by addition of NaI (0.025 g, 0.17 mmol, 1.00 equiv) and K_2_CO_3_ (0.068 g, 0.50 mmol, 3.00 equiv). Purification by chromatography on SiO_2_ afforded **9** (0.036 g, 61%) as a colorless oil: ^1^H NMR (400 MHz, CDCl_3_) δ 7.76 (d, 1 H, *J* = 7.6 Hz), 7.50 (d, 2 H, *J* = 8.0 Hz), 7.22 (d, 2 H, *J* = 8.0 Hz), 7.10 (dd, 1 H, *J* = 8.4 Hz, 1.6 Hz), 6.95 (br s, 1 H), 3.83–3.77 (m, 2 H), 3.72 (s, 3 H), 3.62–3.52 (br s, 2 H), 2.51–2.43 (m, 2 H), 2.41 (s, 3 H), 1.69–1.57 (m, 2 H); ^13^C{^1^H} NMR (100 MHz, CDCl_3_) δ 172.1, 143.6, 136.7, 135.9, 130.6, 130.4, 129.9, 129.6, 127.4, 127.1, 124.9, 52.1, 46.5, 40.5, 26.6, 21.6, 21.4; IR (ATR) 2963 (w), 1736 (m), 1597 (w), 1496 (w), 1340 (m), 1160 (s), 1091 (w), 683 (m) cm^–1^; HRMS (ESI+) calcd for C_19_H_21_NO_4_SNa [M + Na^+^] 382.1089, found 382.1085.

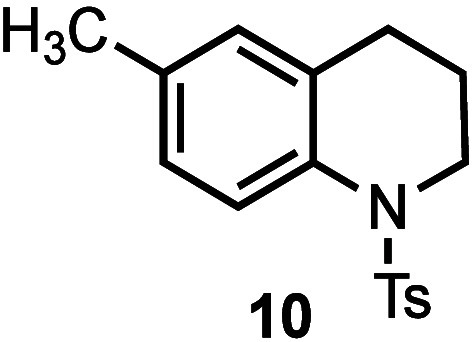



#### 6-Methyl-1-(4-methylbenzenesulfonyl)-1,2,3,4-tetrahydroquinoline **10**

Prepared according General Procedure B from **S10** (0.068 g, 0.20 mmol, 1.0 equiv), **1** (0.127 g, 0.30 mmol, 1.50 equiv), 4CzIPN (0.005 g, 3 mol %), and NiCl_2_(dtbbpy) (0.004 g, 5 mol %) in NMP (2 mL), followed by addition of NaI (0.030 g, 0.20 mmol, 1.00 equiv) and K_2_CO_3_ (0.083 g, 0.60 mmol, 3.00 equiv). Purification by chromatography on SiO_2_ afforded **10** (0.037 g, 61%) as a colorless oil: ^1^H NMR (400 MHz, CDCl_3_) δ 7.67 (d, 1 H, *J* = 8.0 Hz), 7.47 (d, 2 H, *J* = 8.4 Hz), 7.18 (d, 2 H, *J* = 8.0 Hz), 6.99 (d, 1 H, *J* = 8.0 Hz), 6.81 (s, 1 H), 3.80–3.73 (m, 2 H), 2.45–2.35 (m, 2 H), 2.38 (s, 3 H), 2.28 (s, 3 H), 1.62–1.54 (m, 2 H); ^13^C{^1^H} NMR (100 MHz, CDCl_3_) δ 143.4, 136.8, 134.6, 134.3, 130.5, 129.54, 129.52, 127.2, 127.1, 125.0, 46.5, 26.5, 21.6, 21.5, 20.8; IR (ATR) 2923 (w), 2227 (m), 1494 (m), 1339 (m), 1251 (m), 1161 (s), 1090 (m), 812 (m), 677 (s) cm^–1^; HRMS (ESI+) calcd for C_17_H_20_NO_2_S [M + H] 302.1215, found 302.1217.

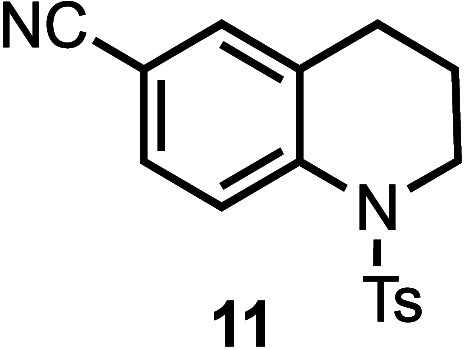



#### 6-Cyano-1-(4-methylbenzenesulfonyl)-1,2,3,4-tetrahydroquinoline **11**

Prepared according the General Procedure B from **S11** (0.088 g, 0.22 mmol, 1.0 equiv), **1** (0.140 g, 0.33 mmol, 1.50 equiv), 4CzIPN (0.005 g, 3 mol %), and NiCl_2_(dtbbpy) (0.004 g, 5 mol %) in NMP (2.2 mL), followed by addition of NaI (0.033 g, 0.20 mmol, 1.00 equiv) and K_2_CO_3_ (0.091 g, 0.66 mmol, 3.00 equiv). Purification by chromatography on SiO_2_ afforded **11** (0.049 g, 71%) as a colorless oil: ^1^H NMR (400 MHz, CDCl_3_) δ 7.90 (d, 1 H, *J* = 8.8 Hz), 7.53 (d, 2 H, *J* = 8.4 Hz), 7.42 (d, 1 H, *J* = 8.0 Hz), 7.31 (s, 1 H), 7.24 (d, 2 H, *J* = 8.4 Hz), 3.86–3.81 (m, 2 H), 2.56 (t, 2 H, *J* = 6.8 Hz), 2.42 (s, 3 H), 1.75–1.66 (m, 2 H); ^13^C{^1^H} NMR (100 MHz, CDCl_3_) δ 144.3, 141.2, 136.2, 133.1, 130.5, 130.3, 139.9, 127.0, 123.9, 118.7, 107.5, 46.7, 26.9, 21.6, 21.2; IR (ATR) 2924 (w), 2227 (m), 1604 (w), 1591 (w), 1490 (m), 1341 (m), 1251 (m), 1159 (s), 1088 (m), 812 (m) cm^–1^; HRMS (ESI+) calcd for C_17_H_16_N_2_O_2_SNa [M + Na^+^] 335.0830, found 335.0844.

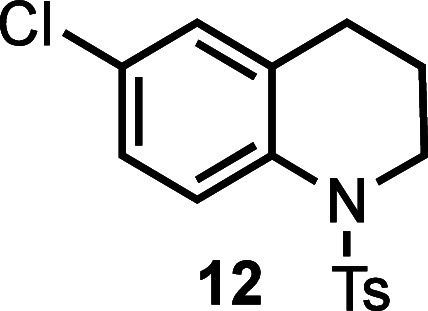



#### 6-Chloro-1-(4-methylbenzenesulfonyl)-1,2,3,4-tetrahydroquinoline **12**

Prepared according to General Procedure B from *o*-bromosulfonamide **S12a** (0.072 g, 0.20 mmol, 1.0 equiv), **1** (0.127 g, 0.30 mmol, 1.50 equiv), 4CzIPN (0.005 g, 3 mol %), and pre-formed NiCl_2_(dtbbpy) (0.004 g, 5 mol %) in NMP (2 mL), followed by addition of NaI (0.030 g, 0.20 mmol, 1.00 equiv) and K_2_CO_3_ (0.083 g, 0.60 mmol, 3.00 equiv). Workup and purification by chromatography on SiO_2_ afforded **12** (0.045 g, 70%) as a colorless solid: mp 81–83 °C.

Prepared according to General Procedure B from *o-*iodosulfonamide **S12b** (0.122 g, 0.30 mmol, 1.0 equiv), **1** (0.191 g, 0.45 mmol, 1.50 equiv), 4CzIPN (0.012 g, 3 mol %), and pre-formed NiCl_2_(dtbbpy) (0.006 g, 5 mol %) in NMP (3 mL), followed by addition of NaI (0.045 g, 0.30 mmol, 1.00 equiv) and K_2_CO_3_ (0.124 g, 0.90 mmol, 3.00 equiv). Workup and purification by chromatography on SiO_2_ afforded **12** (0.053 g, 55%) as a colorless solid. In both cases, the spectroscopic data obtained were consistent with a previous literature report of **12**.^23^

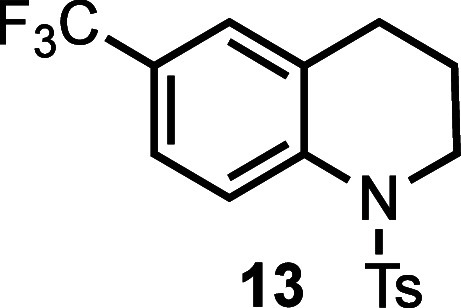



#### 6-Trifluoromethyl-1-(4-methylbenzenesulfonyl)-1,2,3,4-tetrahydroquinoline **13**

Prepared according to General Procedure B from sulfonamide **S13** (0.158 g, 0.40 mmol, 1.0 equiv), **1** (0.254 g, 0.60 mmol, 1.50 equiv), 4CzIPN (0.009 g, 3 mol %), and NiCl_2_(dtbbpy) (0.008 g, 5 mol %) in NMP (4 mL), followed by addition of NaI (0.060 g, 0.40 mmol, 1.00 equiv) and K_2_CO_3_ (0.166 g, 1.20 mmol, 3.00 equiv). Workup and purification by chromatography on SiO_2_ afforded **13** (0.072 g, 51%) as a colorless oil: ^1^H NMR (400 MHz, CDCl_3_) δ 8.11 (s, 1 H), 7.52 (d, 2 H, *J* = 8.0 Hz), 7.29 (d, 1 H, *J* = 8.0 Hz), 7.21 (d, 2 H, *J* = 8.0 Hz), 7.13 (d, 1 H, *J* = 7.6 Hz), 3.88–3.80 (m, 2 H), 2.53 (t, 2 H, J = 6.4 Hz), 2.41 (s, 3 H), 1.75–1.64 (m, 2 H); ^13^C{^1^H} NMR (100 MHz, CDCl_3_) δ 140.0, 137.3, 136.2, 134.0, 129.7, 129.6, 128.9 (q, ^2^*J*_CF_ = 33 Hz), 127.3, 123.9 (q, ^1^*J*_CF_ = 271 Hz), 121.5 (q, ^3^*J*_CF_ = 4 Hz), 121.1 (q, ^3^*J*_CF_ = 5 Hz), 46.4, 26.8, 21.5, 21.2; ^19^F{^1^H} NMR (376 MHz, CDCl_3_) δ −65.6; IR (ATR) 2925 (w), 1507 (w), 1422 (m), 1323 (s), 1251 (m), 1161 (s), 1090 (m), 658 (m) cm^–1^; HRMS (ESI+) calcd for C_17_H_16_NF_3_O_2_SNa [M + Na^+^] 378.0752, found 378.0743.

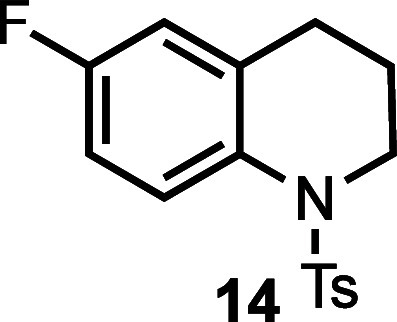



#### 6-Fluoro-1-(4-methylbenzenesulfonyl)-1,2,3,4-tetrahydroquinoline **14**

Prepared according to General Procedure B from sulfonamide **S14** (0.138 g, 0.40 mmol, 1.0 equiv), **1** (0.254 g, 0.60 mmol, 1.50 equiv), 4CzIPN (0.009 g, 3 mol %), and NiCl_2_(dtbbpy) (0.008 g, 5 mol %) in NMP (4 mL), followed by addition of NaI (0.060 g, 0.40 mmol, 1.00 equiv) and K_2_CO_3_ (0.166 g, 1.20 mmol, 3.00 equiv). Workup and purification by chromatography on SiO_2_ afforded **14** (0.061 g, 64%) as a colorless solid: mp 117–119 °C; ^1^H NMR (400 MHz, CDCl_3_) δ 7.81–7.74 (dd, 1 H, *J* = 8.8 Hz, 5.6 Hz), 7.44 (d, 2 H, *J* = 8.4 Hz), 7.19 (d, 2 H, *J* = 8.0 Hz), 6.94–6.86 (m, 1 H), 6.74–6.68 (m, 1 H), 3.80–3.74 (m, 2 H), 2.41–2.35 (m, 2 H), 2.39 (s, 3 H), 1.63–1.55 (m, 2 H); ^13^C{^1^H} NMR (100 MHz, CDCl_3_) δ 160.0 (d, ^1^*J*_CF_ = 243 Hz), 143.6, 136.5, 133.2 (d, ^3^*J*_CF_ = 7 Hz), 132.8 (d, ^4^*J*_CF_ = 3 Hz), 129.6, 127.2 (d, ^3^*J*_CF_ = 7 Hz), 127.1, 115.2 (d, ^2^*J*_CF_ = 22 Hz), 113.5 (d, ^2^*J*_CF_ = 22 Hz), 46.3, 26.6, 21.6, 21.1; ^19^F{^1^H} NMR (376 MHz, CDCl_3_) δ −120.7; IR (ATR) 2952 (w), 1597 (w), 1490 (m), 1340 (m), 1161 (s), 1090 (m), 677 (m) cm^–1^; HRMS (ESI+) calcd for C_16_H_16_NFO_2_SNa [M + Na^+^] 328.0783, found 328.0778.

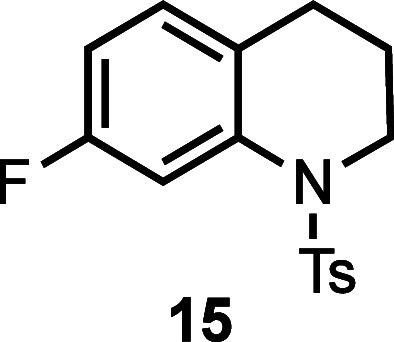



#### 7-Fluoro-1-(4-methylbenzenesulfonyl)-1,2,3,4-tetrahydroquinoline **15**

Prepared according to General Procedure B from *o*-bromosulfonamide **S15** (0.138 g, 0.40 mmol, 1.0 equiv), **1** (0.254 g, 0.60 mmol, 1.50 equiv), 4CzIPN (0.009 g, 3 mol %), and NiCl_2_(dtbbpy) (0.008 g, 5 mol %) in NMP (4 mL), followed by addition of NaI (0.060 g, 0.40 mmol, 1.00 equiv) and K_2_CO_3_ (0.166 g, 1.20 mmol, 3.00 equiv). Workup and purification by chromatography on SiO_2_ afforded **15** (0.067 g, 55%) as a colorless solid: mp 113–115 °C; ^1^H NMR (400 MHz, CDCl_3_) δ 7.59 (dd, 1 H, *J* = 11.2 Hz, 2.4 Hz), 7.52 (d, 2 H, *J* = 8.4 Hz), 7.21 (d, 2 H, *J* = 8.0 Hz), 6.96–6.91 (m, 1 H), 6.76 (td, 1 H, *J* = 8.0 Hz, 2.8 Hz), 3.81–3.76 (m, 2 H), 2.45 (t, 2 H, *J* = 6.8 Hz), 2.38 (s, 3 H), 1.67–1.60 (m, 2 H); ^19^F{^1^H} NMR (376 MHz, CDCl_3_) δ −118.0 (s); ^13^C{^1^H} NMR (100 MHz, CDCl_3_) δ 160.9 (d, ^1^*J*_CF_ = 241 Hz), 143.7, 137.8 (d, ^3^*J*_CF_ = 11 Hz), 136.4, 130.0 (^3^*J*_CF_ = 9 Hz), 129.7, 127.3 (d, ^4^*J*_CF_ = 5 Hz), 127.1, 125.5 (d, ^4^*J*_CF_ = 3 Hz), 111.6 (d, ^2^*J*_CF_ = 22 Hz), 111.3 (d, ^2^*J*_CF_ = 26 Hz), 46.5, 30.6, 26.3, 21.5; IR (ATR) 3087 (w), 2931 (w), 1597 (m), 1486 (m), 1339 (m), 1161 (s), 791 (m), 686 (s), 657 (s) cm^–1^; HRMS (ESI+) calcd for C_16_H_16_NFO_2_SNa [M + Na^+^] 328.0783, found 328.0780.

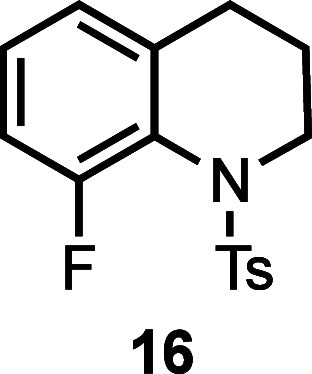



#### 8-Fluoro-1-(4-methylbenzenesulfonyl)-1,2,3,4-tetrahydroquinoline **16**

Prepared according to General Procedure B from *o*-bromosulfonamide **S16** (0.138 g, 0.40 mmol, 1.0 equiv), **1** (0.254 g, 0.60 mmol, 1.50 equiv), 4CzIPN (0.009 g, 3 mol %), and NiCl_2_(dtbbpy) (0.008 g, 5 mol %) in NMP (4 mL), followed by addition of NaI (0.060 g, 0.40 mmol, 1.00 equiv) and K_2_CO_3_ (0.166 g, 1.20 mmol, 3.00 equiv). Workup and purification by chromatography on SiO_2_ afforded **16** (0.076 g, 62%) as a colorless oil: ^1^H NMR (400 MHz, CDCl_3_) δ 7.75 (d, 2 H, J = 8.0 Hz), 7.27 (d, 2 H, *J* = 8.0 Hz), 7.10 (dt, 1 H, *J* = 8.0 Hz, 4.8 Hz), 7.00–6.94 (m, 1 H), 6.88 (d, 1 H, *J* = 8.0 Hz), 3.61 (t, 2 H, *J* = 6.4 Hz), 2.51 (t, 2 H, *J* = 6.8 Hz), 2.43 (s, 3 H), 1.98 (pent, 2 H, *J* = 6.8 Hz); ^19^F{^1^H} NMR (376 MHz, CDCl_3_) δ −116.2 (s); ^13^C{^1^H} NMR (100 MHz, CDCl_3_) δ 157.4 (d, ^1^*J*_CF_ = 250 Hz), 143.6, 137.4, 136.5, 129.5, 127.5, 126.8 (d, ^4^*J*_CF_ = 9 Hz), 125.5 (^3^*J*_CF_ = 12 Hz), 124.0, 114.2 (d, ^2^*J*_CF_ = 21 Hz), 45.6, 25.4, 23.2, 21.6; IR (ATR) 2952 (w), 1585 (w), 1474 (m), 1337 (m), 1263 (m), 1156 (s), 1091 (m), 841 (m), 665 (s) cm^–1^; HRMS (ESI+) calcd for C_16_H_16_NFO_2_SNa [M + Na^+^] 328.0783, found 328.0779.

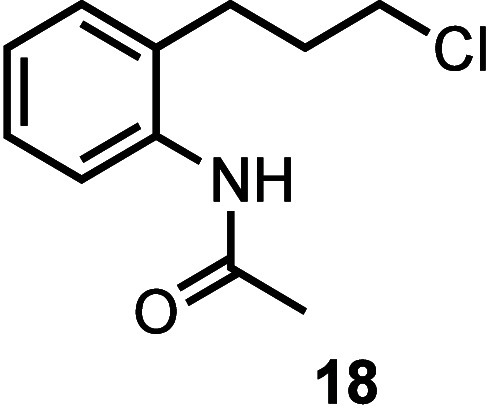



#### 2′-(3-Chloropropyl)acetanilide **18**

Prepared according to General Procedure B from *o-*bromoacetanilide **17**(24) (0.086 g, 0.40 mmol, 1.0 equiv), 3-chloropropyl bis(catacholato)silicate (0.254 g, 0.60 mmol, 1.50 equiv), 4CzIPN (0.009 g, 3 mol %), and pre-formed NiCl_2_(dtbbpy) (0.008 g, 5 mol %) in NMP (4 mL). Workup and purification by chromatography on SiO_2_ afforded **18** (0.049 g, 58%) as a thick oil that solidified on standing: mp 81–84 °C; ^1^H NMR (400 MHz, CDCl_3_) δ 7.66 (d, 1 H, *J* = 8.0 Hz), 7.34 (br s, 1 H), 7.48 (d, 1 H, *J* = 7.6 Hz), 7.25–7.02 (m, 3 H), 3.50 (t, 2 H, *J* = 6.0 Hz), 2.71 (t, 2 H, *J* = 7.2 Hz), 2.14 (s, 3 H), 2.04–1.94 (m, 2 H); ^13^C{^1^H} NMR (100 MHz, CDCl_3_) δ 169.1, 135.5, 132.5, 129.9, 127.3, 125.9, 124.8, 44.9, 32.9, 27.9, 24.3; IR (ATR) 3258 (w), 3040 (w), 1660 (s), 1529 (s), 1498 (s), 1442 (s), 1370 (w), 1265 (w), 751 (s) cm^–1^; HRMS (ESI+) calcd for C_11_H_14_NClONa [M + Na^+^] 234.0662, found 234.0659.

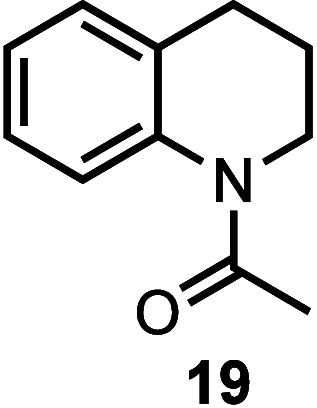



#### 1-Acetyl-1,2,3,4-tetrahydroquinoline **19**

A solution of amide **18** (34 mg, 0.16 mmol) in DMA (1 mL) was treated with NaH (6 mg, 0.16 mmol, 60% dispersion in oil). The reaction mixture immediately became dark in color. The reaction mixture was stirred at rt for 5 h, at which point it was quenched with water (20 mL) and extracted with ethyl acetate (3 × 20 mL). The organic layer was washed with brine (30 mL), dried (Na_2_SO_4_), and concentrated. Purification by chromatography on SiO_2_ afforded the desired product (20 mg, 71%) as a colorless oil. The spectroscopic data of **19** was in accord with previous reports.^25^

## Data Availability

The data underlying this study are available in the published article and its Supporting Information.

## Supporting Information Available

Additional experimental procedures, additional control experiments, and NMR spectra of synthesized products (PDF)
