# Supplementary material for: 3-Chloropropylbis(catecholato)silicate as a Bifunctional Reagent for the One-Pot Synthesis of Tetrahydroquinolines from o-Bromosulfonamides
Source: J Org Chem. 2024 Feb 27;89(6):4191–8. doi: 10.1021/acs.joc.3c02267 (PMC10949236; doi:10.1021/acs.joc.3c02267)

Supporting Information for

"3-Chloropropylbis(catecholato)silicate as a Bifunctional Reagent for the One-pot Synthesis of Tetrahydroquinolines from o-Bromosulfonamides"

Noah Brodsky, Nidheesh Phadnis, Mohamed Ibrahim, Isabel M. Andino, Inés Blanc Giro and John A. Milligan*

Department of Biological and Chemical Sciences, College of Life Sciences, Thomas Jefferson University, 4201 Henry Ave, Philadelphia, Pennsylvania 19144, United States

Table of contents

General information S2
Photochemical reaction setup S3
Synthesis of reagent **1** and *o*-bromo sulfonamide precursors S4
Control/comparison experiments S6
Gram-scale reaction procedure S9
Unsuccessful substrates S10
References S11
NMR spectra of new compounds S12

General Information

All chemical transformations requiring inert atmospheric conditions used Schlenk line techniques with a 4-port dual-bank manifold. Nitrogen was used to provide the inert atmosphere. NMR spectra (^{1}H, ^{13}C, and ^{19}F) were obtained at 298 K. ^{1}H NMR spectra were referenced to residual, non-deuterated chloroform (δ 7.26). ^{13}C NMR spectra were referenced to $CDCl_3$ (δ 77.3). ^{19}F NMR spectra were referenced using hexafluorobenzene (δ –164.9) as an internal standard and run with C-F/C-H decoupling. TLC analysis was performed using hexanes/EtOAc as the eluant and visualized using permanganate stain, p-anisaldehyde stain, and/or UV light. Flash chromatography was carried out using standard column chromatography on silica gel (60 Å porosity, 32-63 µm).

Photochemical reaction setup

Blue LED irradiation was conducted by using two Kessil PR 160L lamps emitting at 440 nm at a maximum power of 45 W (light intensity of 399 mW/cm^2). More information on the spectral distribution and intensity of these lamps can be found at: https://kessil.com/products/science_PR160L.php

The lamps were supported by a Kessil PR160 rig that held each lamp 7 cm from the reaction vessel. The reactions were conducted in standard borosilicate glass vials affixed with PTFE septa (to maintain the reaction under an N_2 atmosphere). No filters were used. A picture of the reaction setup is shown below:

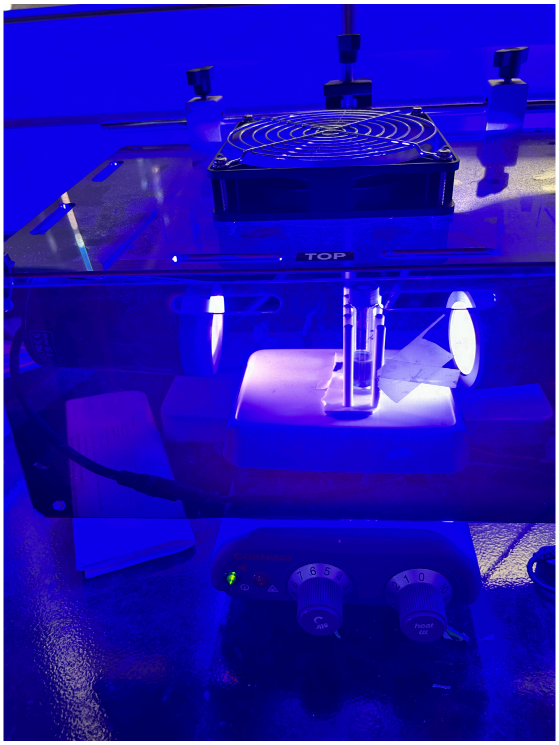

Synthesis of reagent 1 and *o*-bromo sulfonamide precursors

The organosilicate reagent **1** (3-chloropropylbis(catecholato)silicate) was prepared according to previous reports.[1] A detailed procedure for preparing this and related reagents was published in *Organic Syntheses*.[2]

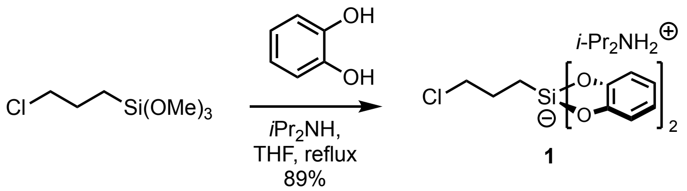

The following sulfonamide precursors were synthesized as reported in the corresponding references:

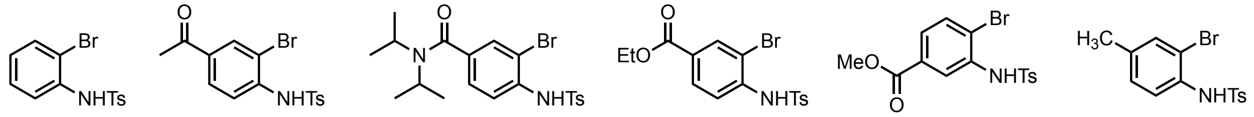

2 ref. 3 | **S4** ref. 3 | **S5** ref. 3 | **S7** ref. 3 | **S8** ref. 4 | **S10** ref. 3

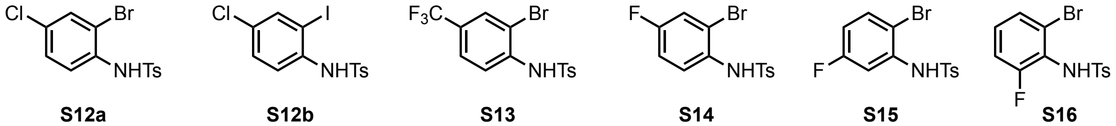

S12a ref. 3 | **S12b** ref. 5 | **S13** ref. 3 | **S14** ref. 3 | **S15** ref. 6 | **S16** ref. 7

Synthesis of new *o*-bromosulfonamide precursors

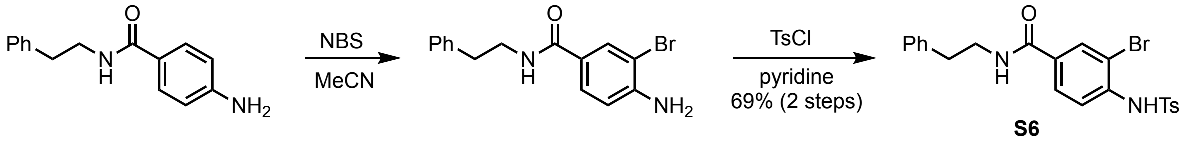

Bromination: To a solution of aniline[8] (0.247 g, 1.03 mmol, 1.00 equiv.) in acetonitrile (5 mL) at rt was added *N*-bromosuccinimide (0.182 g, 1.03 mmol, 1.0 equiv.). The reaction mixture was warmed to rt and stirred for 16 h. The reaction was partitioned between water and CH_2Cl_2 (30 mL each). The organic layer was dried and concentrated to afford the brominated product (0.347 g) as a brown-tinted sticky foam. The material was used without further purification.

Representative general procedure A (sulfonylation of aniline precursors): A solution of amide (0.327 g, 1.02 mmol) in pyridine (2 mL) was treated with tosyl chloride (0.195 g, 1.02 mmol, 1.00 equiv). The reaction was stirred at rt for 18 h, then was diluted with water (25 mL) and extracted with CH_2Cl_2 (2 x 30 mL). The extracts were washed with 1 M HCl (50 mL) and brine (50 mL), then dried (Na_2SO_4) and concentrated. Purification by chromatography on SiO_2 afforded **S6** (333 mg, 69% over 2 steps) as a colorless foam: ^{1}H NMR (400 MHz, $CDCl_3$) δ 7.84 (d, 1 H, *J* = 2.0 Hz), 7.66 (d, 2 H, *J* = 8.4 Hz), 7.62 (d, 1 H, *J* = 8.4 Hz), 7.52 (dd, 1 H, *J* = 9.2 Hz, 1.6 Hz), 7.34-7.27 (m, 2 H), 7.26-7.16 (m, 6 H), 6.28-6.21 (br s, 1 H), 3.65 (q, 2 H, *J* = 6.8 Hz), 2.89 (t, 2 H, *J* = 6.8 Hz), 2.37 (s, 3 H); ^{13}C{^{1}H} NMR (100 MHz, $CDCl_3$) δ 165.3, 144.7,

138.7, 137.4, 135.5, 132.0, 131.9, 129.9, 128.77, 128.75, 127.3, 126.73, 126.69, 120.6, 114.9, 41.3, 35.6, 21.6; IR (ATR) 3312 (w), 2927 (w), 1636 (m), 1488 (s), 1307 (m), 1163 (s), 1090 (m), 911 (m), 813 (m), 700 (s), 661 (s) cm^{-1}; HRMS (ESI+) calc'd for $C_{22}H_{22}BrN_2O_3S$ [M+H] 473.0535, found 473.0535.

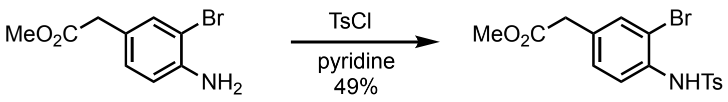

S9

Prepared according to General Procedure A from the corresponding o-bromoaniline[9] (1.04 g, 4.30 mmol), pyridine (5 mL) and tosyl chloride (0.817 g, 4.30 mmol, 1.00 equiv). Purification by chromatography on SiO_2 afforded sulfonamide **S9** (0.833 g, 49%) as a colorless solid: mp 99–101 °C; ^{1}H NMR (400 MHz, $CDCl_3$) δ 7.67 (d, 2 H, *J* = 8.0 Hz), 7.62 (d, 1 H, *J* = 8.0 Hz), 7.38 (s, 1 H), 7.24 (d, 2 H, *J* = 8.0 Hz), 7.20 (d, 1 H, *J* = 8.0 Hz), 6.98 (s, 1 H), 3.71 (s, 3 H), 3.55 (s, 2 H), 2.41 (s, 3 H); ^{13}C{^{1}H} NMR (100 MHz, $CDCl_3$) δ 171.2, 144.3, 135.9, 133.7, 133.2, 132.2, 129.7, 129.6, 127.3, 122.3, 115.6, 52.3, 39.9, 21.6; IR (ATR) 3238 (w), 2955 (w), 1723 (m), 1494 (w), 1427 (m), 1332 (m), 1258 (s), 1154 (s), 1087 (w), 813 (m), 682 (s) cm^{-1}; HRMS (ESI+) calc'd for $C_{16}H_{16}BrNO_4SNa$ [M+Na$^+$] 418.9881, found 418.9882

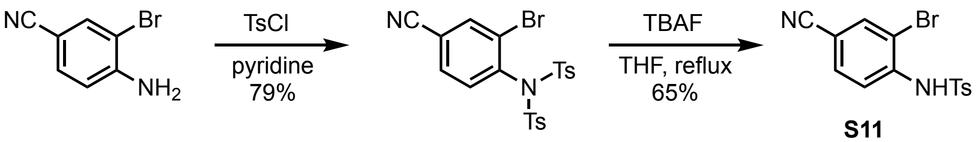

S11

*Attempted sulfonylation of the corresponding aniline to provide **S11** produced a mixture of mono and doubly sulfonylated material under several conditions. The following sequence was therefore used to prepare **S11**:*

A solution of commercially available nitrile (0.591 g, 3.00 mmol) in ethanol (1.5 mL, 0.5 M) was treated with tosyl chloride (0.630 g, 3.30 mmol, 1.10 equiv). The reaction was stirred at rt for 16 h, at which point the reaction had developed a colorless precipitate. Crude LC-MS indicated the reaction contained a mixture of starting material, monosulfonylated, and disulfonylated material. Additional pyridine (3 mL) and tosyl chloride (0.630 g, 3.30 mmol, 1.10 equiv) was added. The reaction was stirred at rt for an additional 14 h, at which point additional colorless precipitate formed. This solid was isolated by filtration, washed with water, then dried at 70 °C for 1 h to afford the double sulfonamide (1.31 g, 79%) as a buff solid.

To a 100 mL round bottomed flask charged with the doubly sulfonylated intermediate (1.31 g, 2.60 mmol, 1 equiv) in THF (26 mL, 0.1 M) was added tetrabutylammonium fluoride (5.2 mL, 5.2 mmol, 2.00 equiv, 1 M in THF), which caused immediate formation of a dark red solution. The reaction mixture was then heated to 80 °C and stirred for 16 h. Upon completion, the reaction mixture was allowed to cool to room temperature, concentrated, then partitioned between water and EtOAc (30 mL each). The organic layer was dried and concentrated to give a semi-solid-mass. This solid was recrystallized from ethanol to give **S11** (0.590 g, 65%) as tan-colored needles: Melting point 144-147 °C; ^{1}H NMR (400 MHz, $CDCl_3$) δ 7.77-7.75 (m, 1 H), 7.75-7.73 (m, 2 H), 7.71 (s, 1 H), 7.54 (dd, 1 H, *J* = 8.4 Hz, 2.0 Hz), 7.45 (s, 1 H), 7.29 (dd, 1 H, *J* = 8.0 Hz, 0.4 Hz), 2.41 (s, 3 H); ^{13}C{^{1}H} NMR (100 MHz, $CDCl_3$) δ 145.1, 139.2, 136.2, 135.3, 132.5, 130.0, 127.3, 120.0, 117.0, 113.9, 108.8, 21.7; IR (ATR) 3224 (m), 2234 (m), 1596 (m), 1307 (s), 1187 (m), 1046 (w), 900 (s), 662 (s) cm^{-1}; HRMS (ESI+) calc'd for $C_{14}H_{11}BrN_2O_2SNa$ [M+Na$^+$] 372.9622, found 372.9624

Control/comparison experiments

Solvent variations:

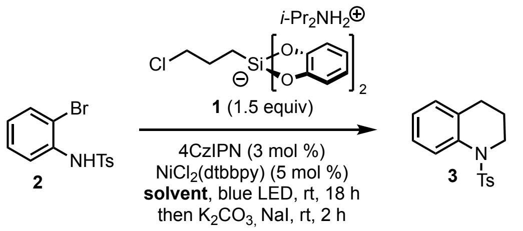

solvent	yield (%)[a]
NMP	97
DMF	85
DMA	67
EtOAc	41
DMSO	28
acetone	0
THF	0
CH_2Cl	0
NMP + H_2O[b]	0
NMP[c]	49

[a]HPLC yield determined using caffiene as an internal standard
[b]Reaction conducted in NMP (2 mL) with water (0.1 mL) added
[c]Reaction conducted without flushing the vial with N_2 prior to irradiation

Photocatalyst variations:

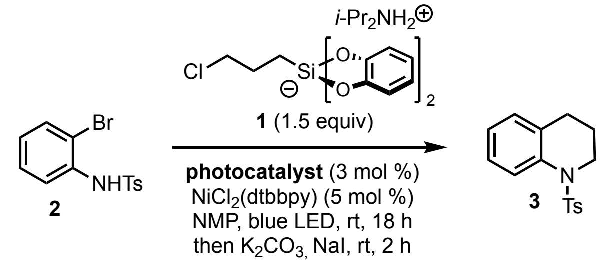

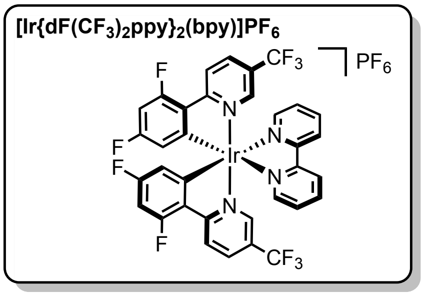

photocatalyst	yield (%)[a]
4CzIPN	97
$[Ru(bpy)_3](PF_6)_2$	90
$[Ir\{dF(CF_3)_2ppy\}_2(bpy)]PF_6$	84
Cl-4CzIPN	41
eosin Y	0
MesAcr	0
4CzIPN[b]	0
none	0

[a]HPLC yield determined using caffiene as an internal standard
[b]reaction done in the absence of light

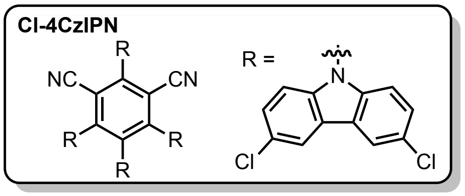

Nickel variations:

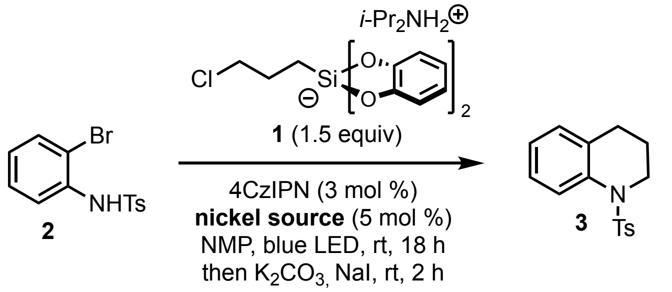

nickel source	yield (%)[a]
$NiCl_2$•dtbbpy	97
$NiCl_2$•dme + dtbbpy[b]	56
$NiCl_2$•phenanthroline	<5
$NiCl_2$•dme	<5
no nickel	0

[a]HPLC yield determined using caffiene as an internal standard
[b]nickel and ligand (5 mol % each) added without pre-formation of a ligated complex

Silicate reagent variations:

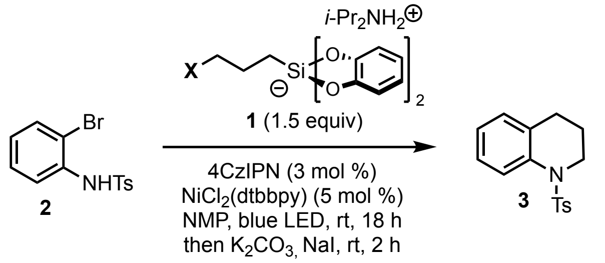

X	reagent **1** equiv.	yield (%)[a]
Cl	1.5	97
Cl	1.1	54
Br	1.5	0
I	1.5	0

[a]HPLC yield determined using caffiene as an internal standard

Base variations:

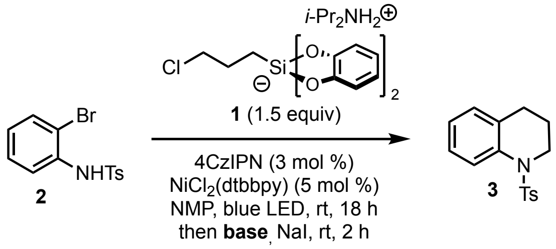

base	yield (%)[a]
K_2CO_3	97
K_3PO_4	94

[a]HPLC yield determined using caffiene as an internal standard

Nitrogen substituent variations:

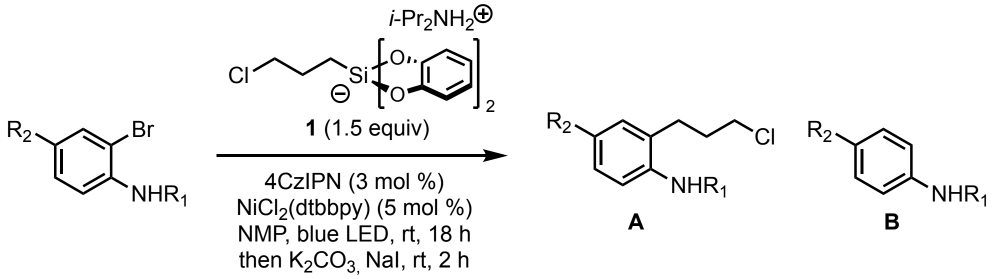

R_1	R_2	result
H	H	complex reaction mixture
$COCH_3$	H	**A**, 58%, (see text)
$COCF_3$	H	recovered starting material
Ms	H	recovered starting material
Boc	CO_2Et	protodehalogenated product **B** + trace **A**

Gram-scale reaction

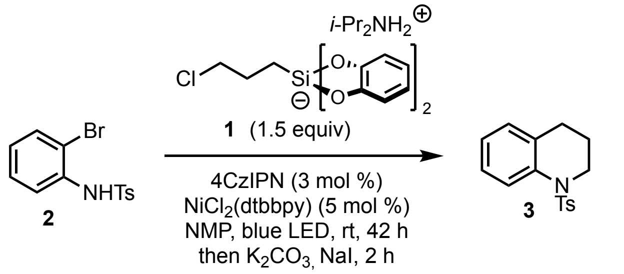

A 100 mL round bottom was charged with **2** (1.01 g, 3.10 mmol, 1.00 equiv), silcate reagent **1** (1.93 g, 4.65 mmol, 1.50 equiv), 4CzIPN (0.073 g, 0.093 mmol, 3 mol %) and pre-formed $NiCl_2$•dtbpy (0.062 g, 0.15 mmol, 5 mol %). The flask was then sealed with a rubber septum and flushed with N_2 gas. Under nitrogen, NMP (30 mL) was added. The flask was clamped in between two Kessil lamps and irradiated for 42 h, at which point LC-MS analysis of a reaction aliquot indicated completion. Without any workup, the reaction mixture was treated with NaI (0.465 g, 3.10 mmol, 1.00 equiv) and K_2CO_3 (1.28 g, 9.30 mmol, 3.00 equiv). The color of the reaction mixture changed from bright yellow to a very dark brown after the addition of the salts. After stirring for 2 h, the reaction mixture was then partitioned between 1 M NaOH and EtOAc (75 mL each). The aqueous layer was extracted with additional EtOAc (50 mL). The combined organic layers were washed with water (50 mL) and brine (50 mL), then dried and concentrated. The product was purified using flash column chromatography over SiO_2 using a mobile phase comprised of Hexanes/EtOAc (0-50% EtOAc in hexanes) to afford 0.580 g (65%) of the desired product as a pale-yellow solid.

Unsuccessful substrates

The following substrates gave complex mixtures with little to no product formation upon subjection to the standard reaction conditions:

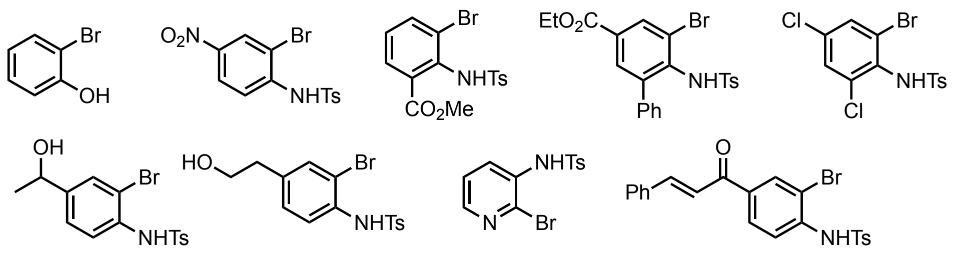

The following electron rich arene substrates gave the desired cyclized product as the major product in modest (30-50%) yield. However, these products were prone to decomposition and were contaminated with unidentified side products of very similar polarity that could not be completely removed by chromatography:

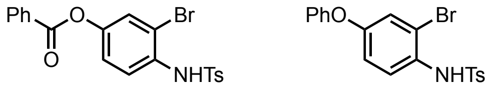

The following hydrazone did successfully undergo the cross-coupling/cyclization reaction, but the tetrahydroquinoline product was unstable and decomposed within hours of purification:

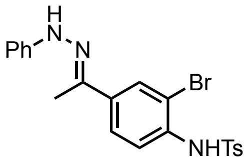

References

1. Pantaine, L. R. E.; Milligan, J. A.; Matsui, J. K.; Kelly, C. B.; Molander, G. A. *Org. Lett.* **2019**, *21*, 2317
2. a) Lin, K.; Kelly, C. B.; Jouffroy, M.; Molander, G. M. *Org. Synth.* **2017**, *94*, 16; b) Kelly, C. B.; Milligan, J. A.; Jouffroy, M. *Org. Synth.* **2022**, *99*, 342.
3. Vaith, J. Rodina, D.; Spaulding, G. C.; Paradine, S. M. *J. Am. Chem. Soc.* **2022**, *144*, 6667.
4. Tao, M.; Tu, Y.; Liu, Y.; Wu, H.; Liu, L.; Zhang, J. *Chem. Sci.* **2020**, *11*, 6283.
5. Ono, Y.; Higuchi, K.; Yamaguchi, M.; Sugino, K.; Nakazaki, A.; Adachi, M.; Nishikawa, T. Synlett **2023**, *34*, 364.
6. Zhao, Q.; Li, M.; Xue, X.; Chen, J.; Xiao, W. *Org. Lett.* **2019**, *21*, 3861.
7. Chen, D.; Chen, Y.; Ma, Z.; Zou, L.; Li, J.; Liu, Y. *J. Org. Chem.* **2018**, *83*, 6805.
8. Patel, A. M.; Meena, P.; Jahan, K.; Bharatam, P. V.; Verma, A. K. *Org. Lett.* **2021**, *23*, 565.
9. Long, C.; Ni, S.; Su, M.; Wang, X.; Tan, W. *ACS Catal.* **2020**, *10*, 13641.

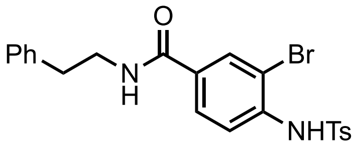

S6

^{1}H NMR

400 MHz, $CDCl_3$

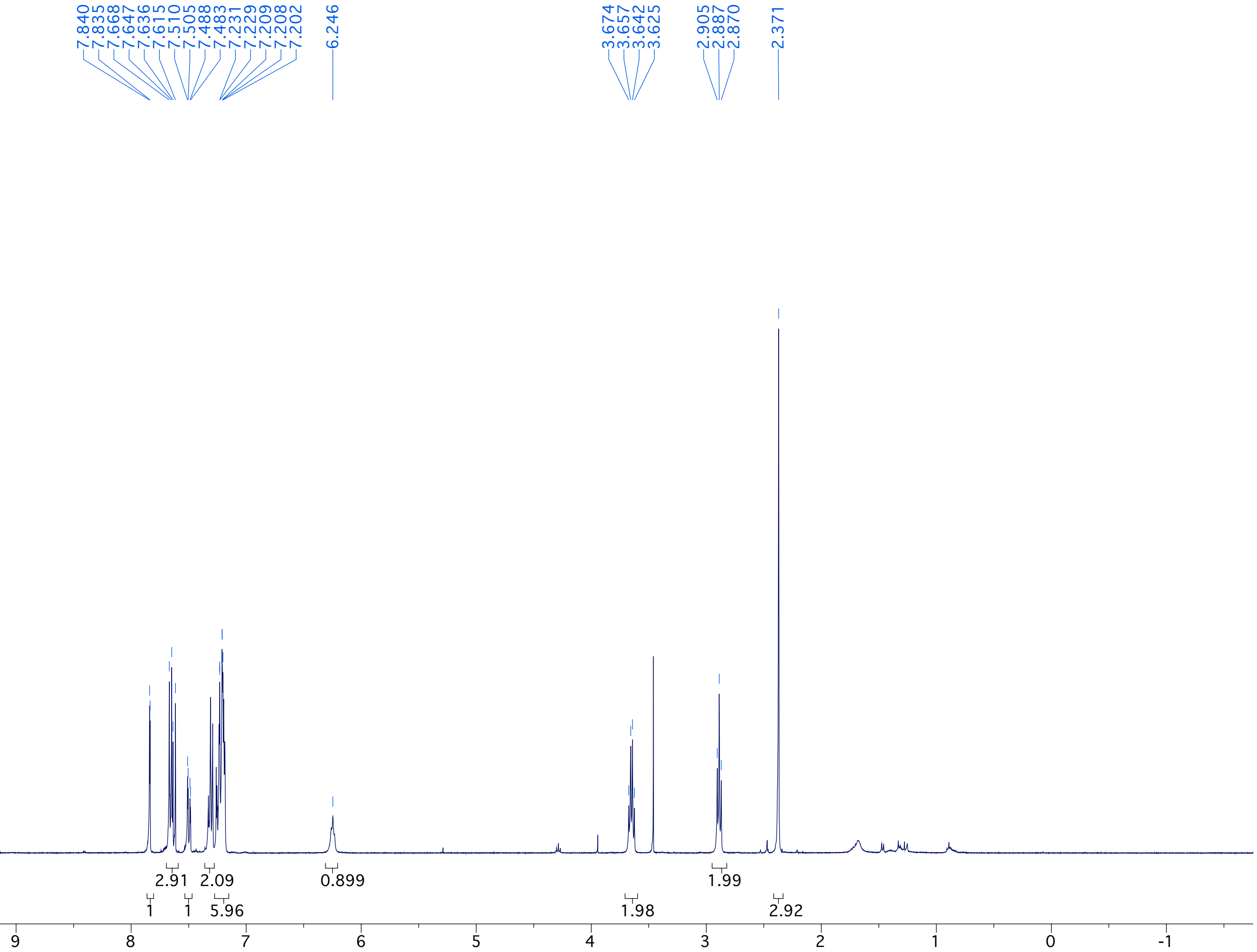

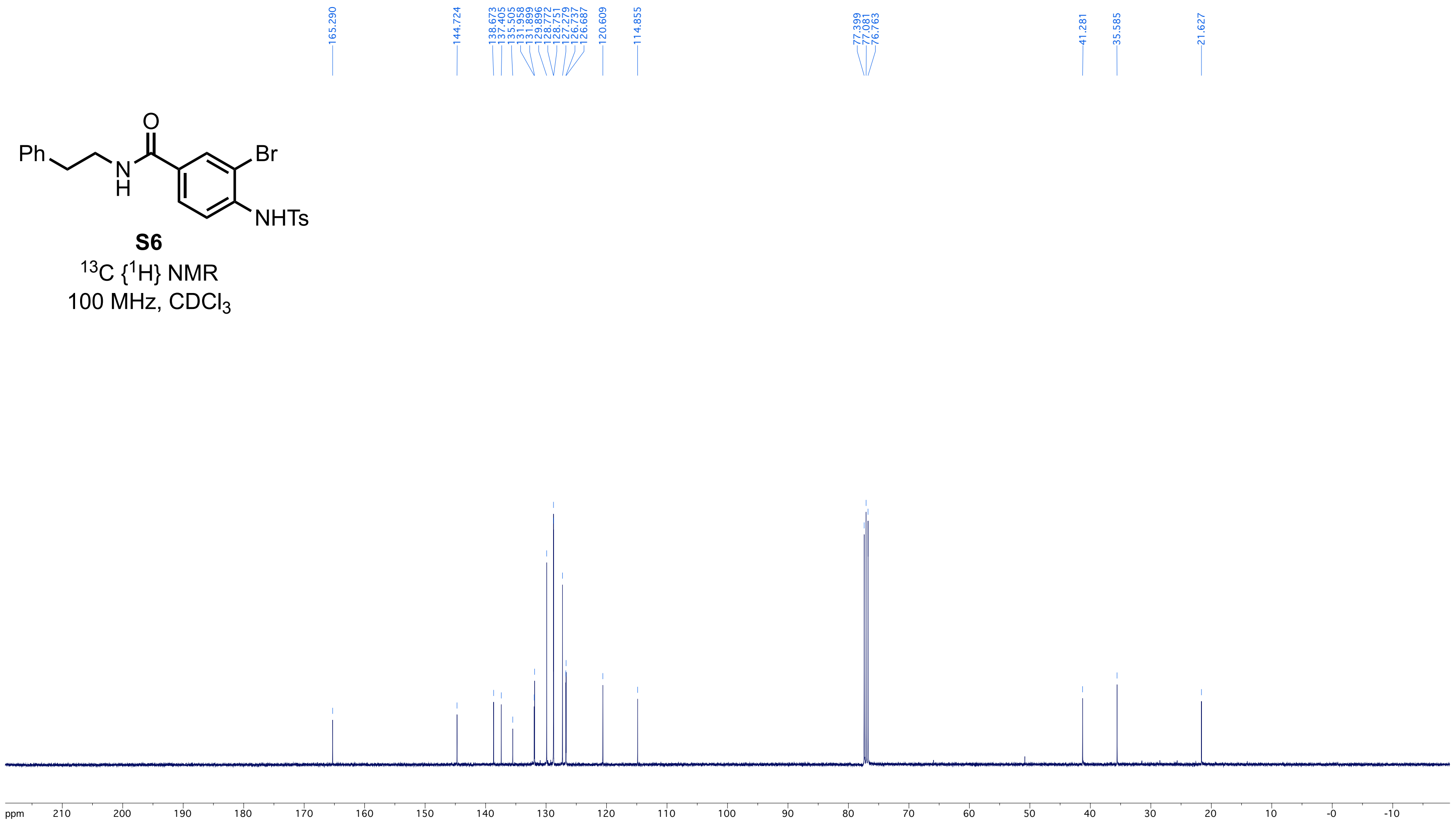

S6

$^{13}C\ \{^1H\}$ NMR

100 MHz, $CDCl_3$

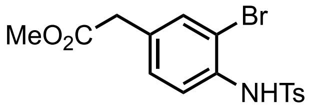

S9

^{1}H NMR

400 MHz, $CDCl_3$

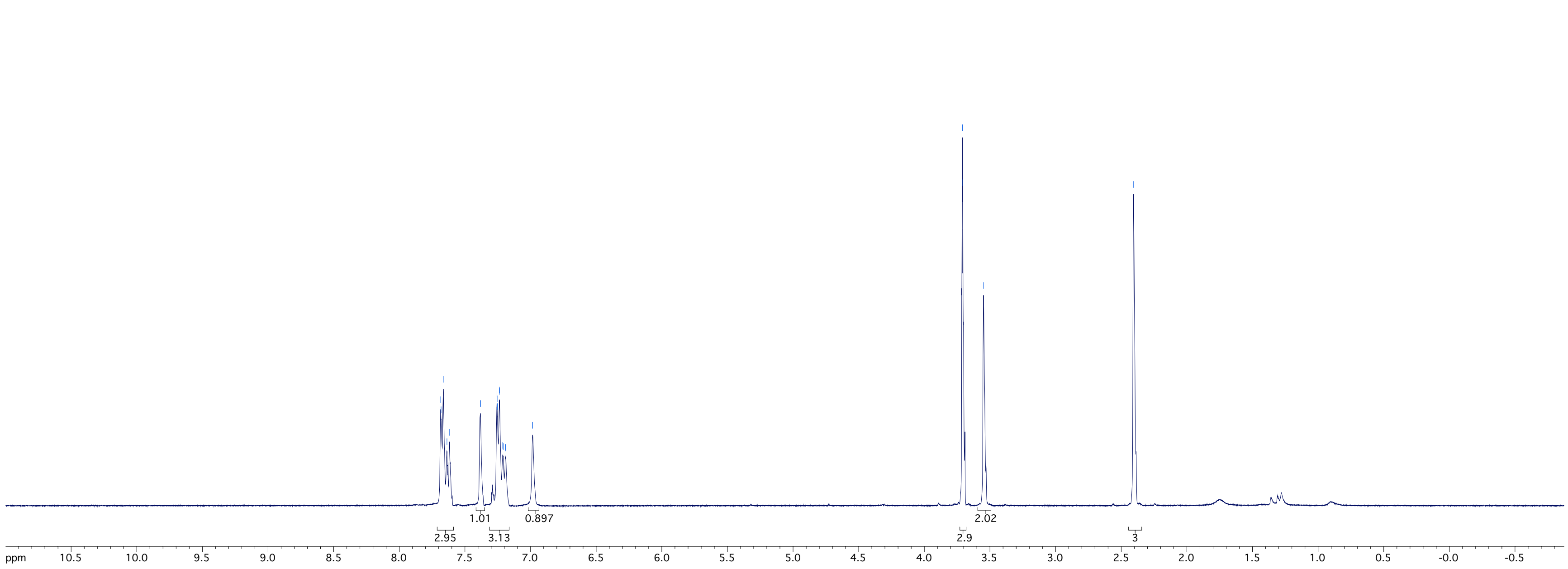

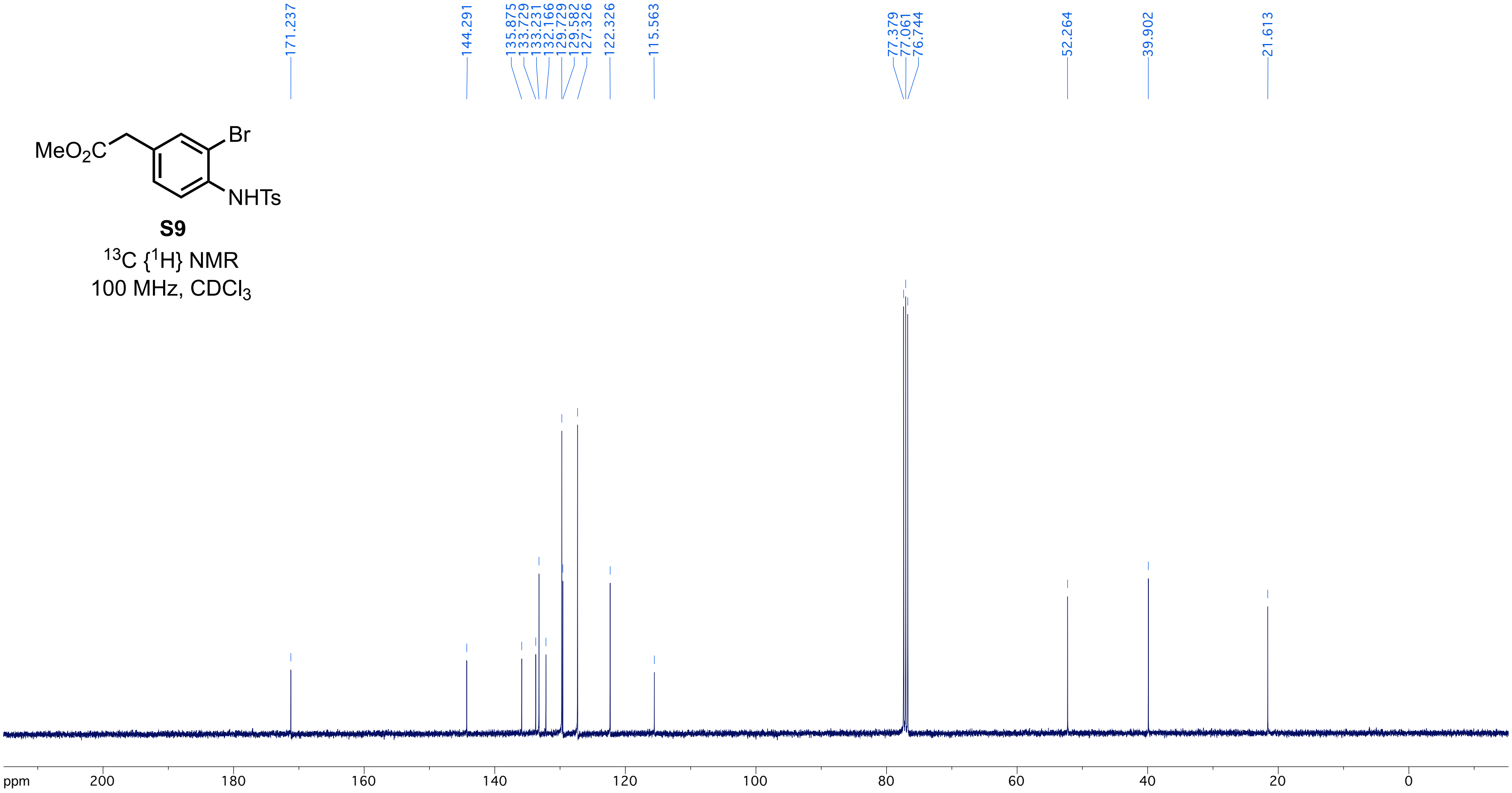

MeO2C
Br
NHTs
S9
13C {1H} NMR
100 MHz, CDCl3
171.237
144.291
135.875
133.729
133.231
132.166
129.729
129.582
127.326
122.326
115.563
77.379
77.061
76.744
52.264
39.902
21.613
ppm
200
180
160
140
120
100
80
60
40
20
0

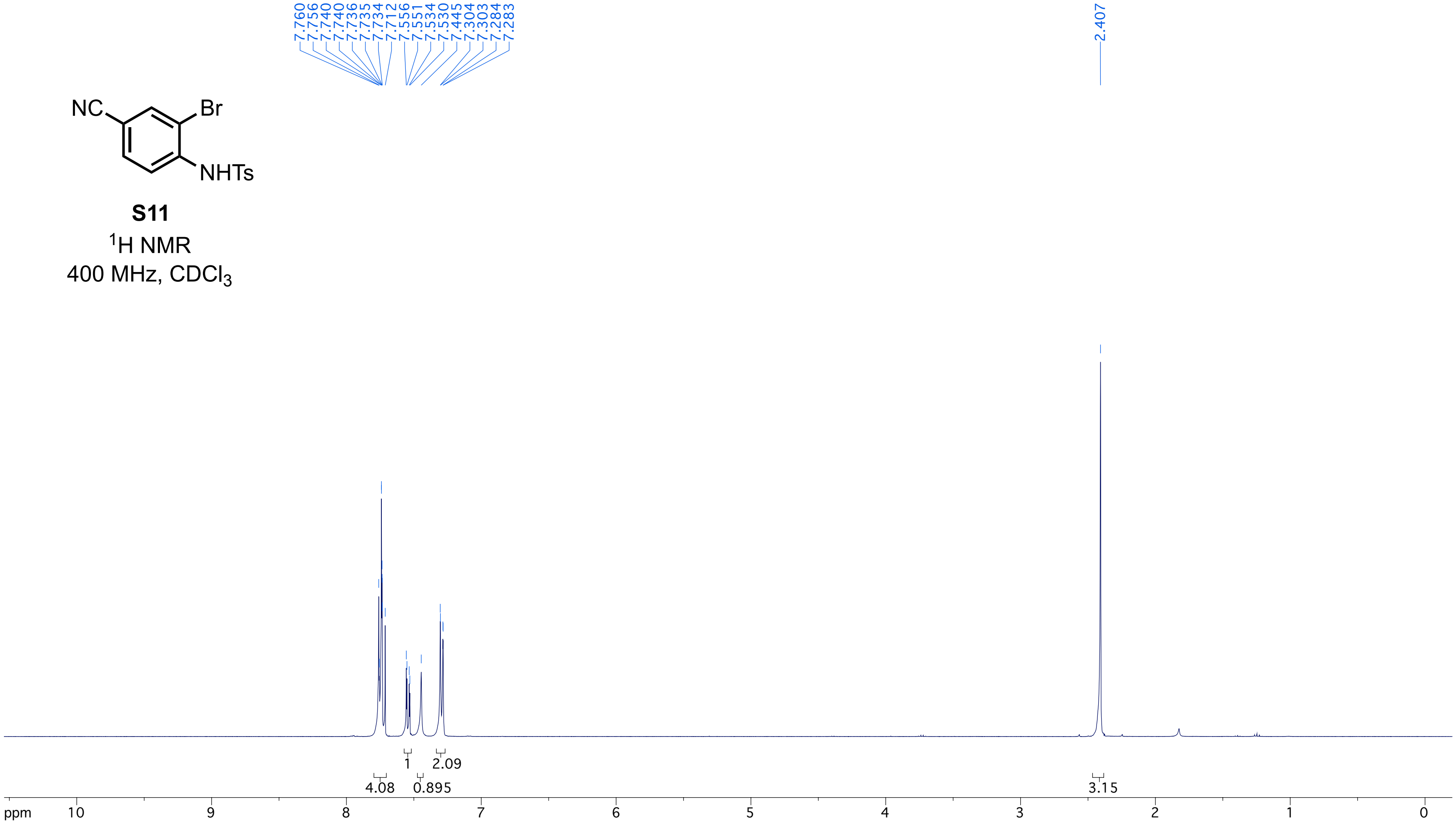

S11

^{1}H NMR

400 MHz, $CDCl_3$

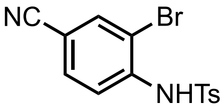

S11

$^{13}C\ \{^{1}H\}$ NMR

100 MHz, $CDCl_3$

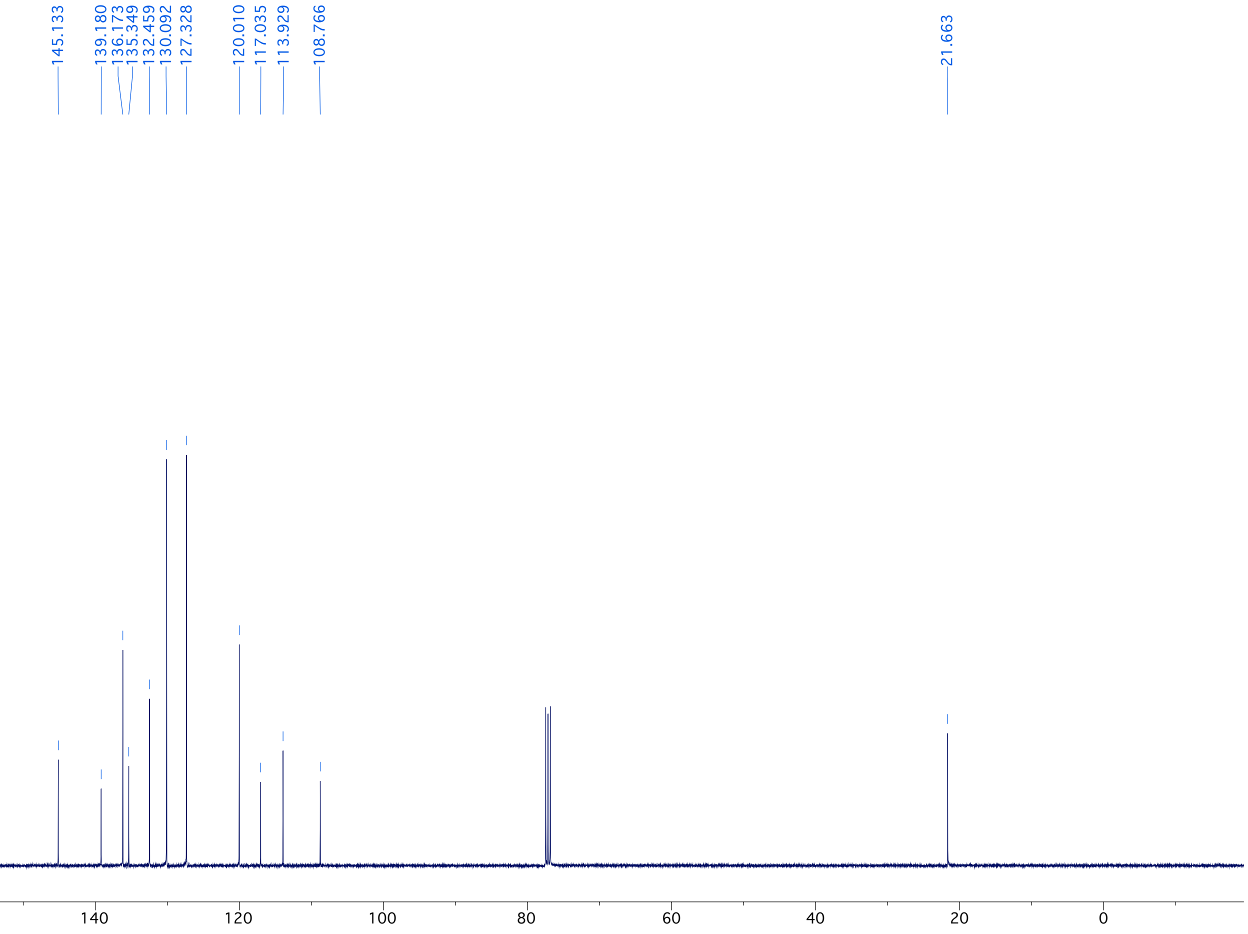

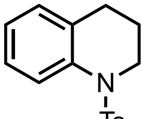

3

^{1}H NMR

400 MHz, $CDCl_3$

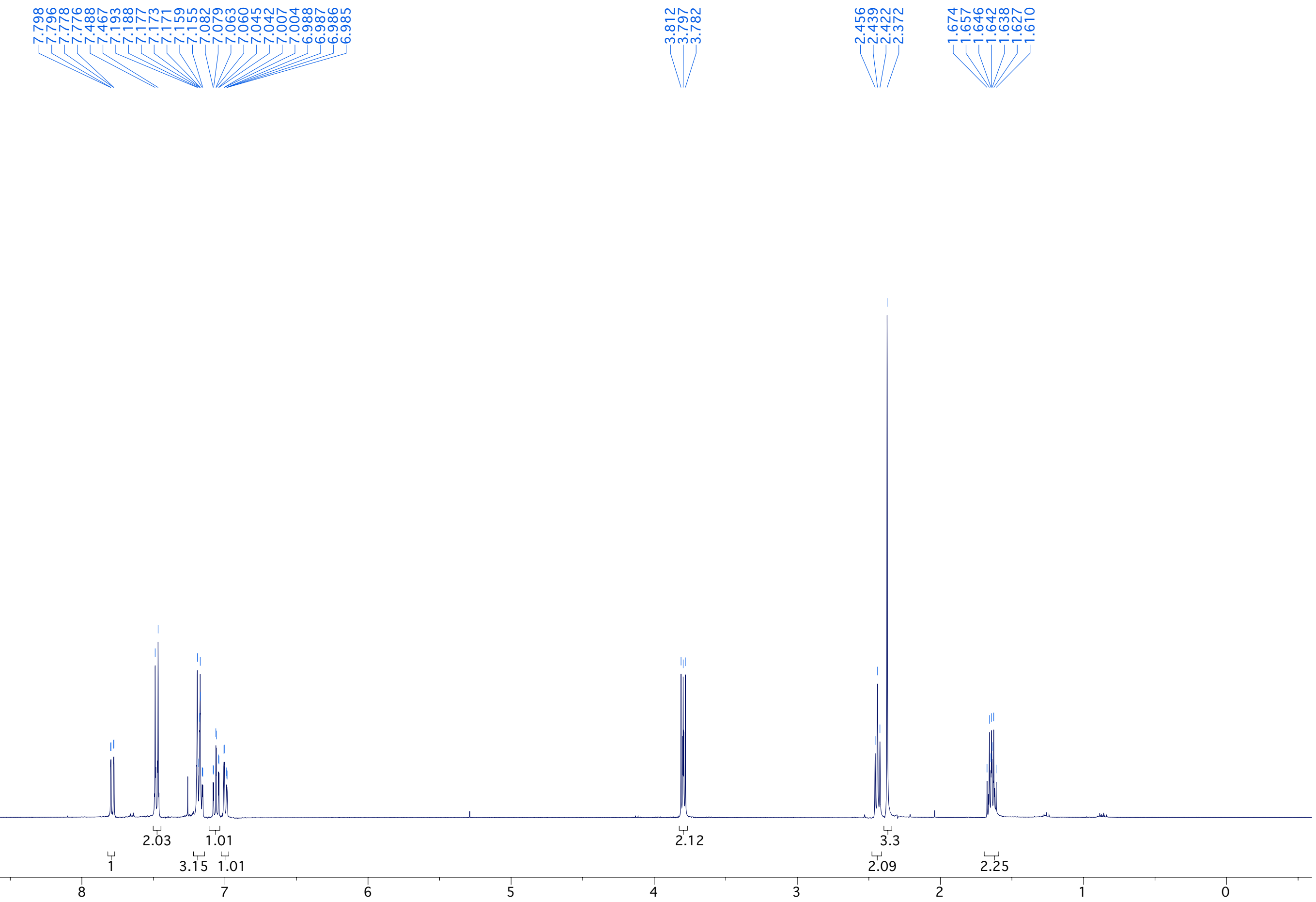

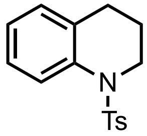

3

$^{13}C\ \{^1H\}$ NMR

100 MHz, $CDCl_3$

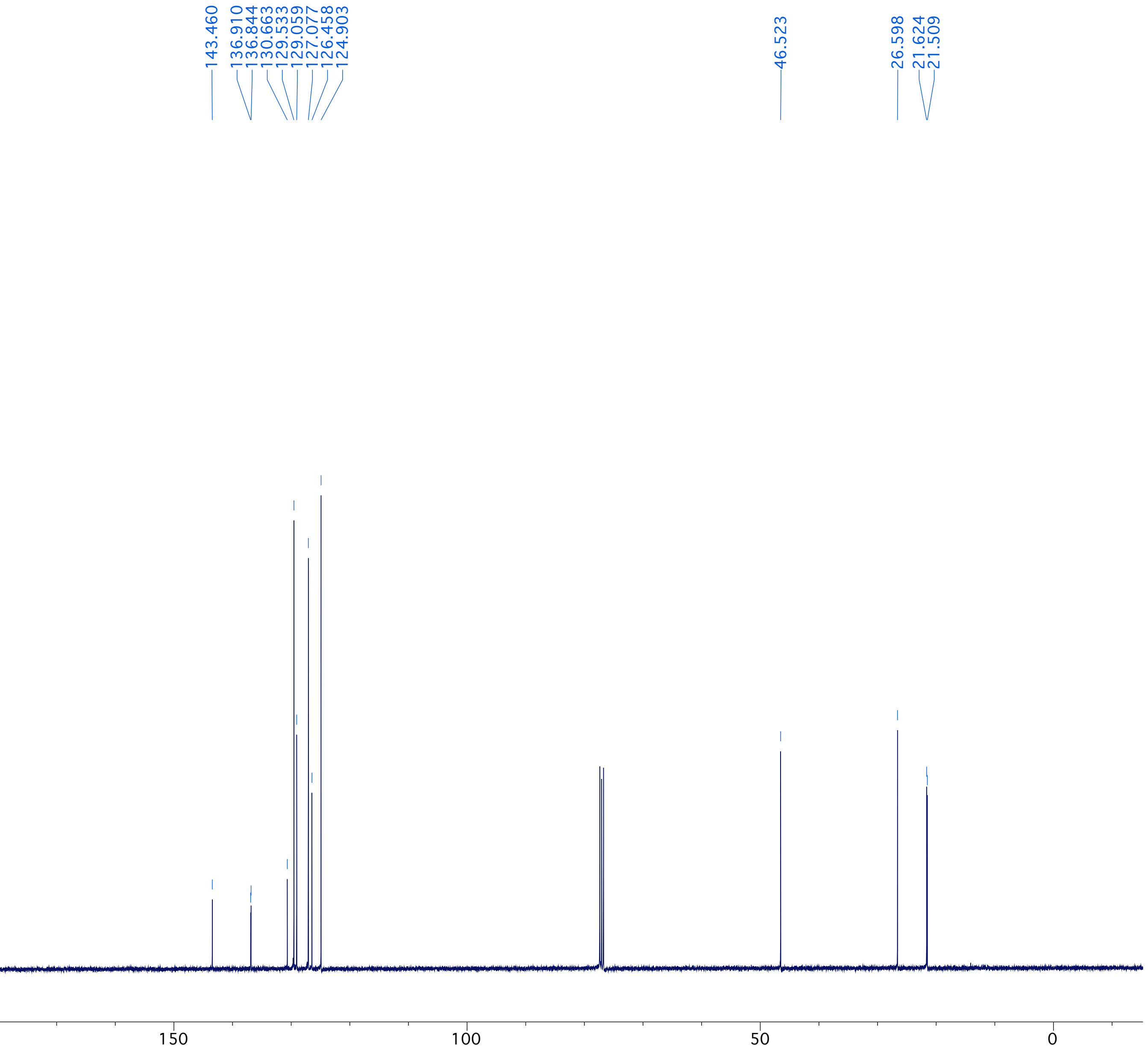

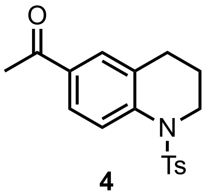

4

^{1}H NMR

400 MHz, $CDCl_3$

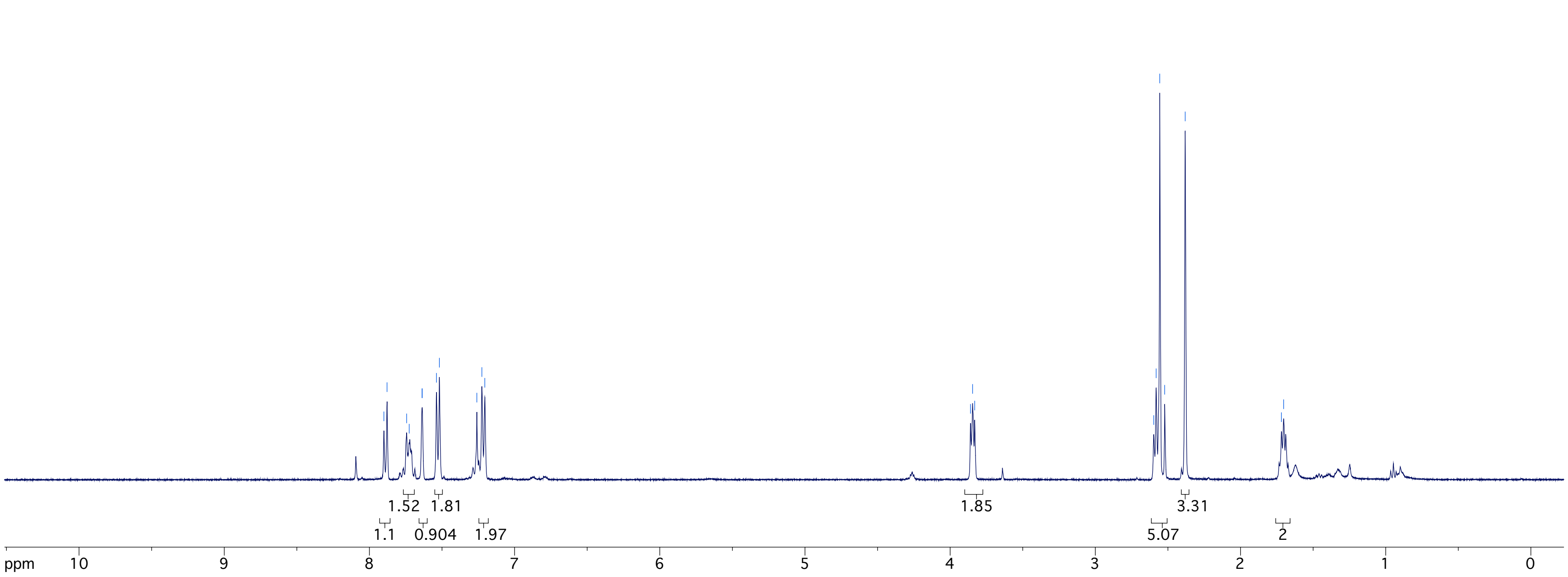

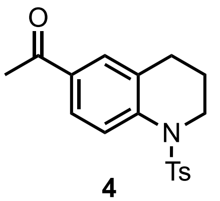

4

$^{13}C\ \{^{1}H\}$ NMR

100 MHz, $CDCl_3$

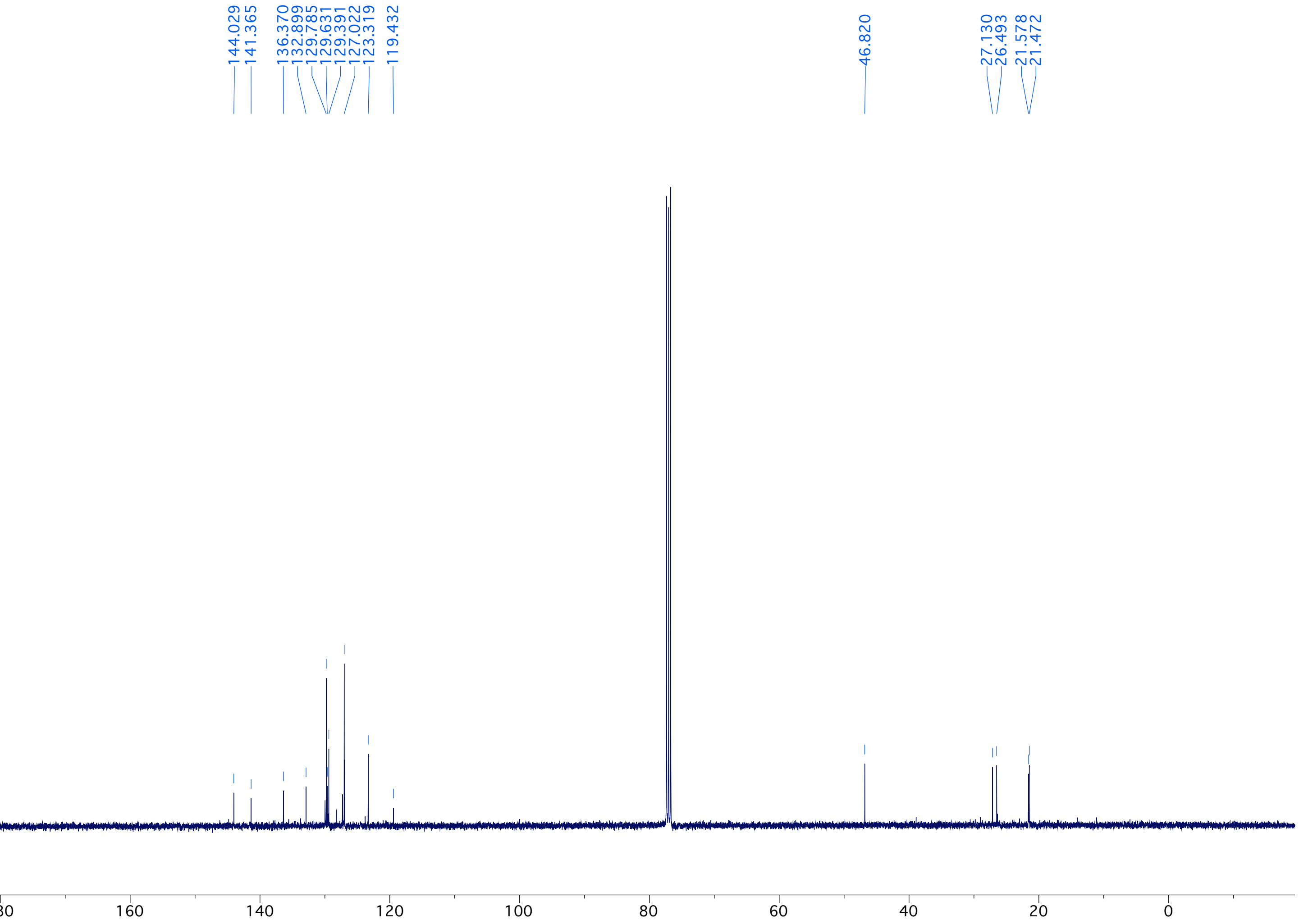

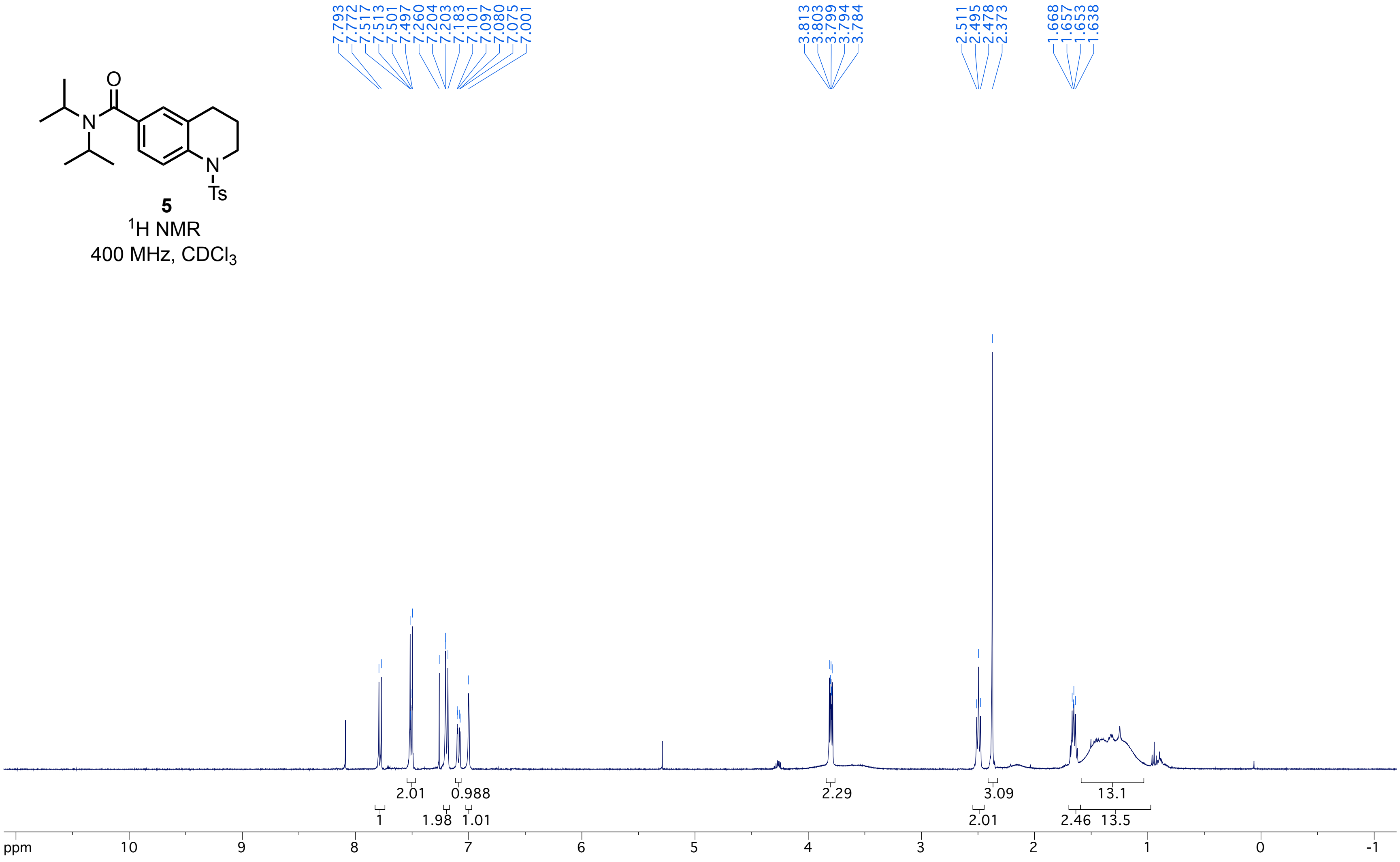

O
N
N
Ts
5
^{1}H NMR
400 MHz, $CDCl_3$
7.793 7.772 7.517 7.513 7.501 7.497 7.260 7.204 7.203 7.183 7.101 7.097 7.080 7.075 7.001
3.813 3.803 3.799 3.794 3.784
2.511 2.495 2.478 2.373
1.668 1.657 1.653 1.638
2.01 0.988 2.29 3.09 13.1
1 1.98 1.01 2.01 2.46 13.5
ppm 10 9 8 7 6 5 4 3 2 1 0 -1

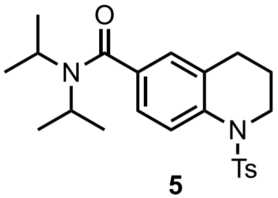

5

^{13}C {^{1}H} NMR

100 MHz, $CDCl_3$

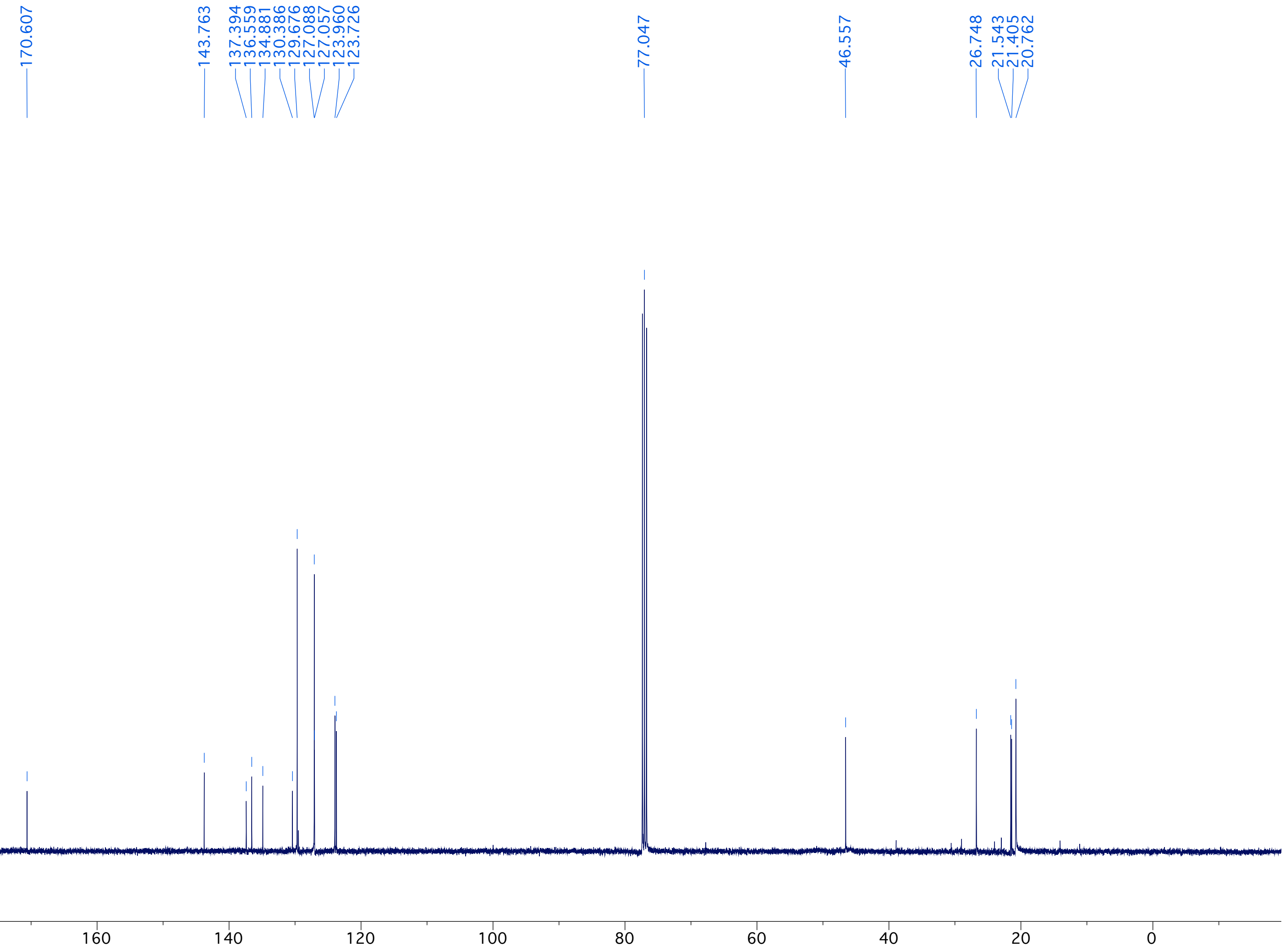

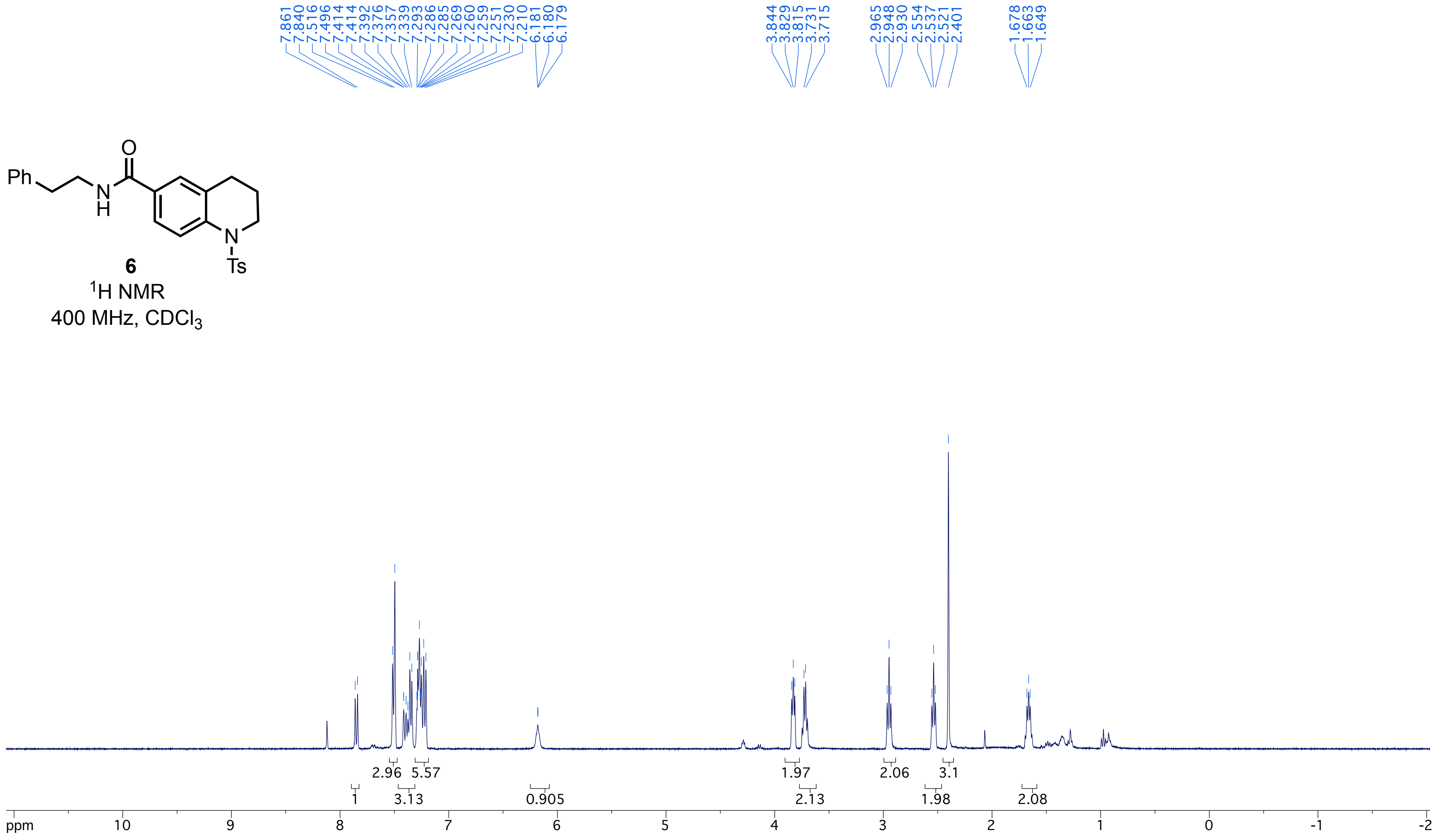

6

^{1}H NMR

400 MHz, $CDCl_3$

6

^{13}C {^{1}H} NMR

100 MHz, $CDCl_3$

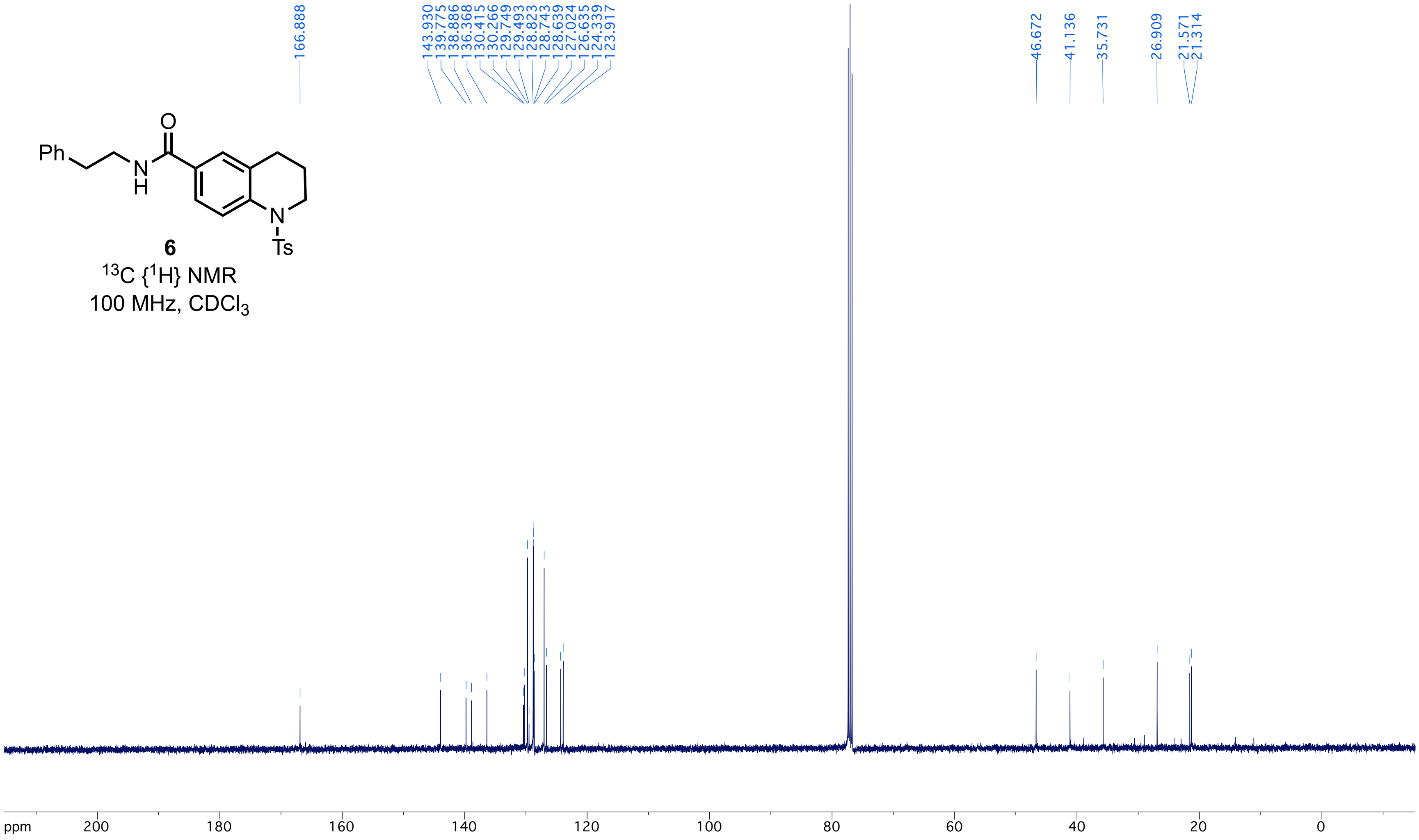

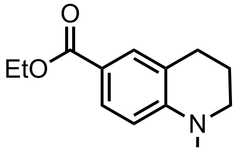

7

^{1}H NMR
400 MHz, $CDCl_3$

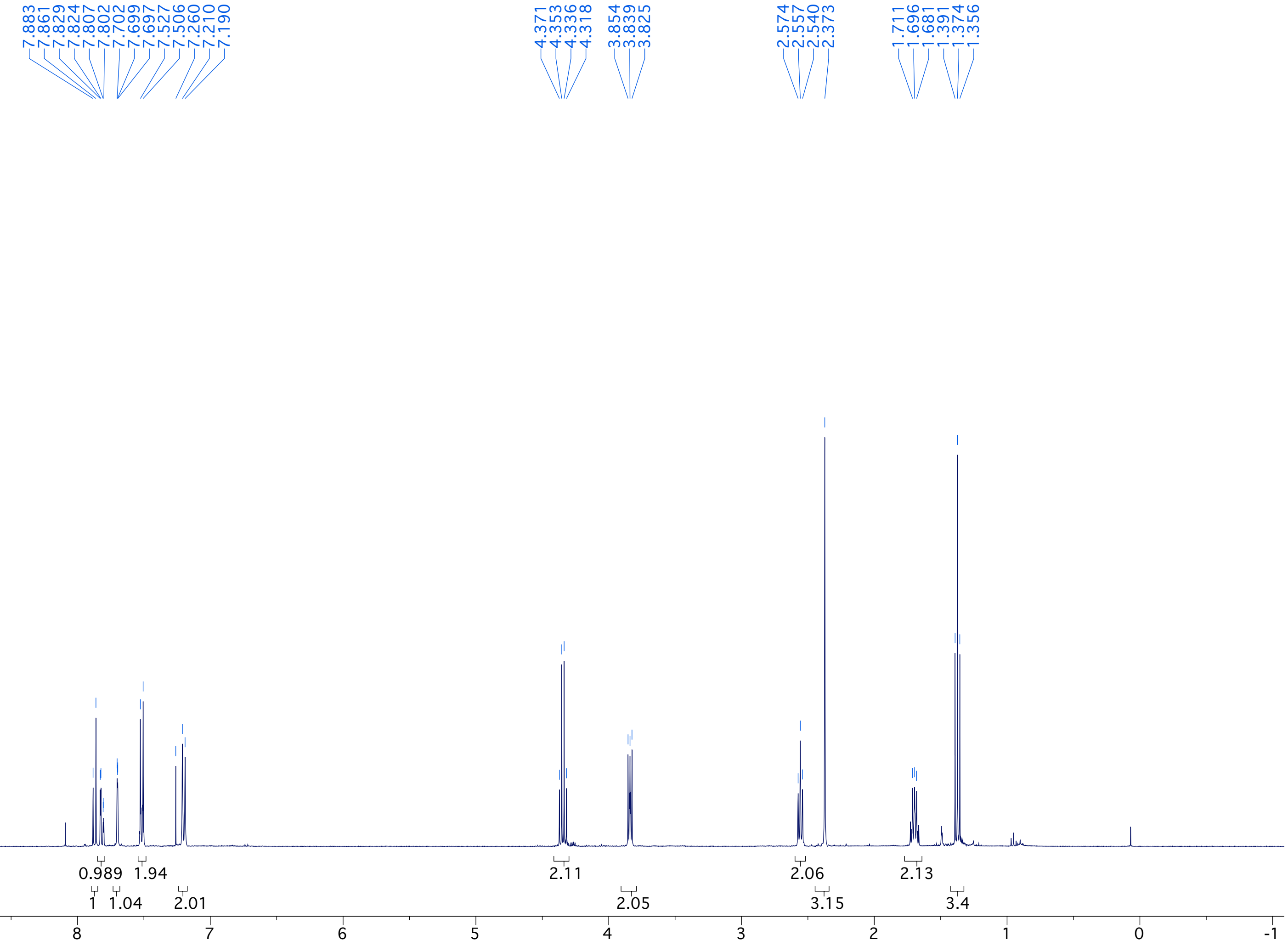

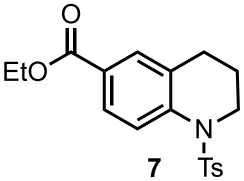

7

$^{13}C\ \{^1H\}$ NMR

100 MHz, $CDCl_3$

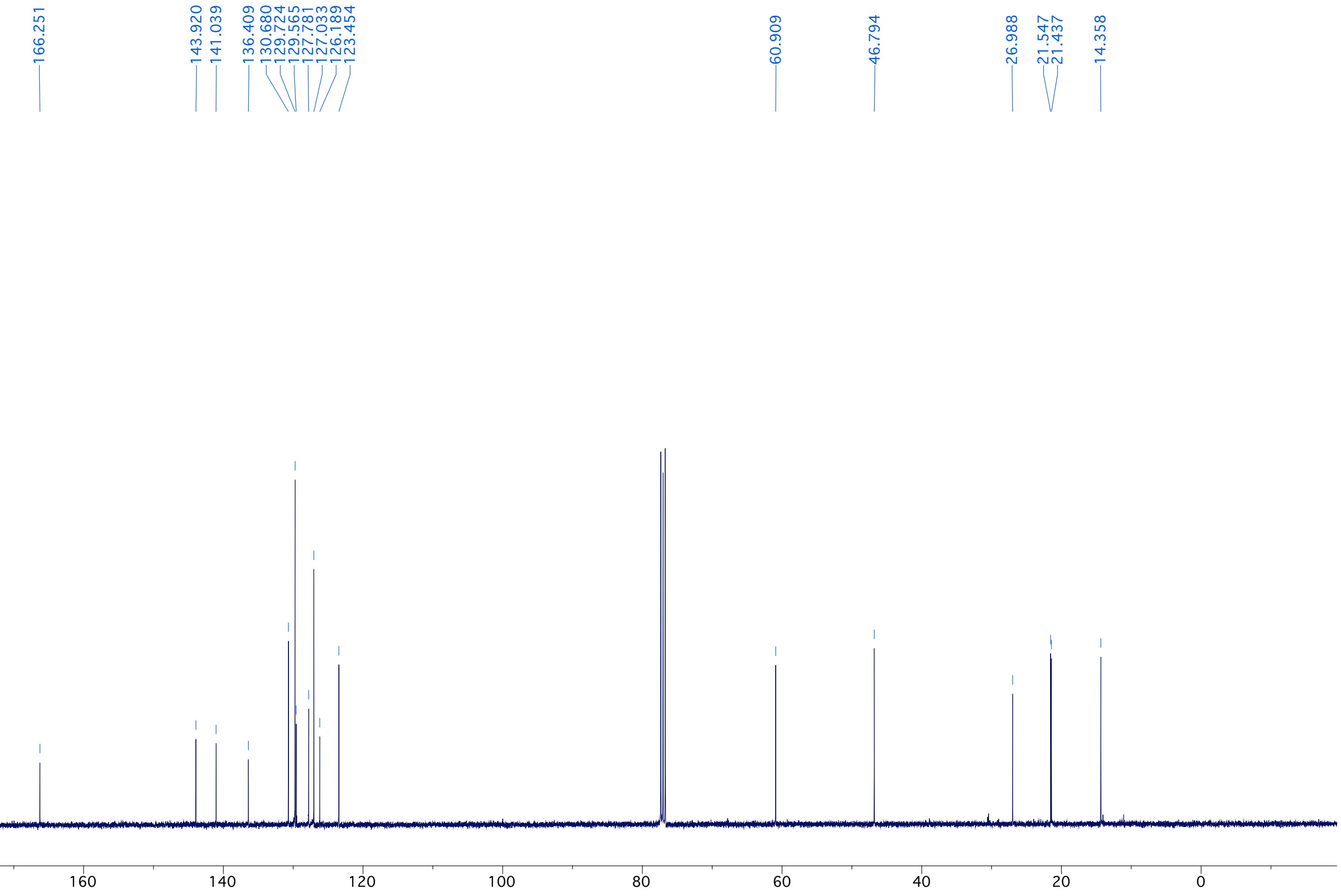

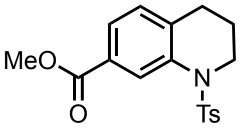

8
^1H NMR
400 MHz, $CDCl_3$

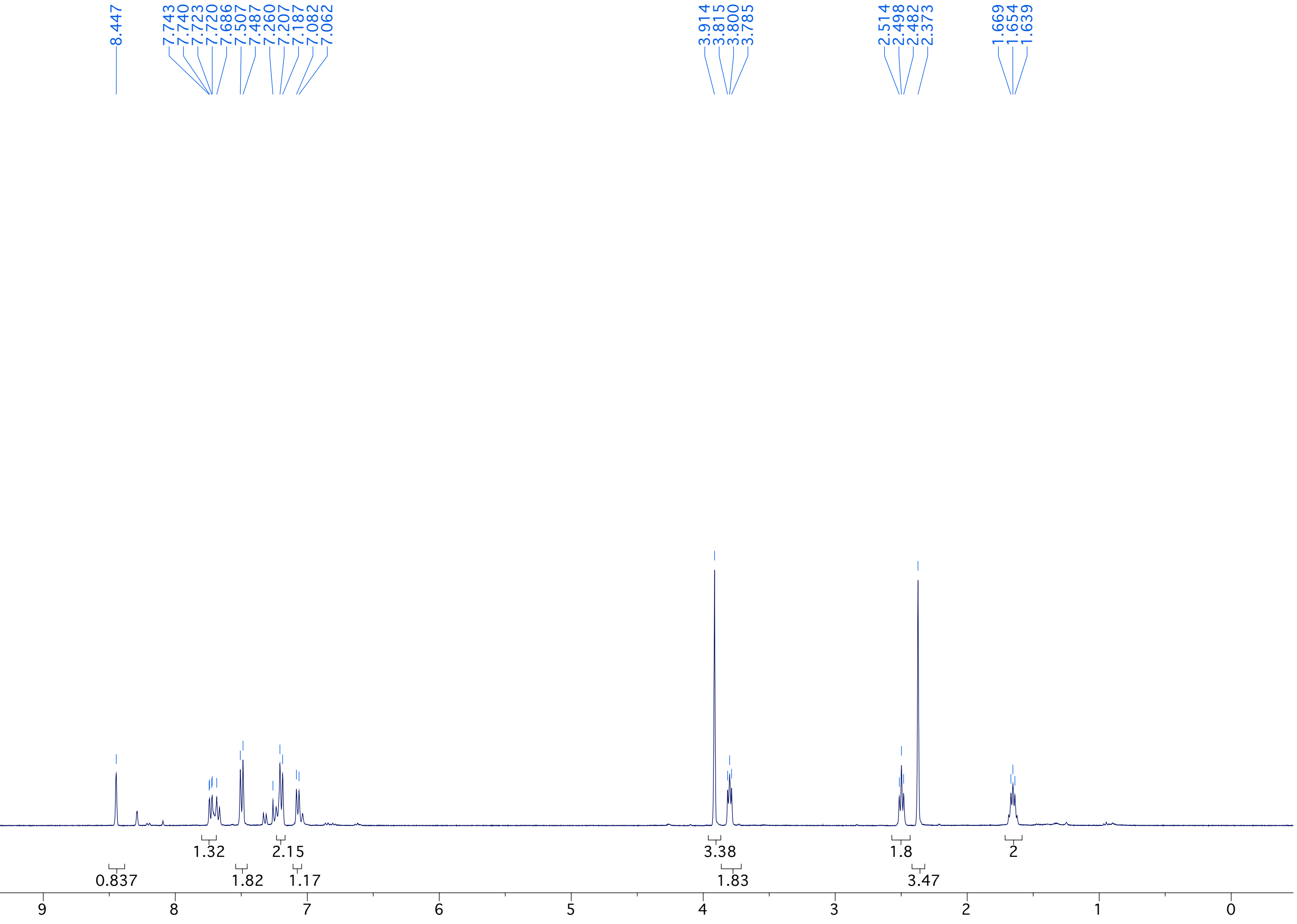

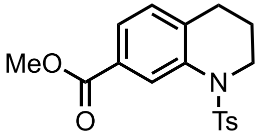

8

^{13}C {^{1}H} NMR

100 MHz, $CDCl_3$

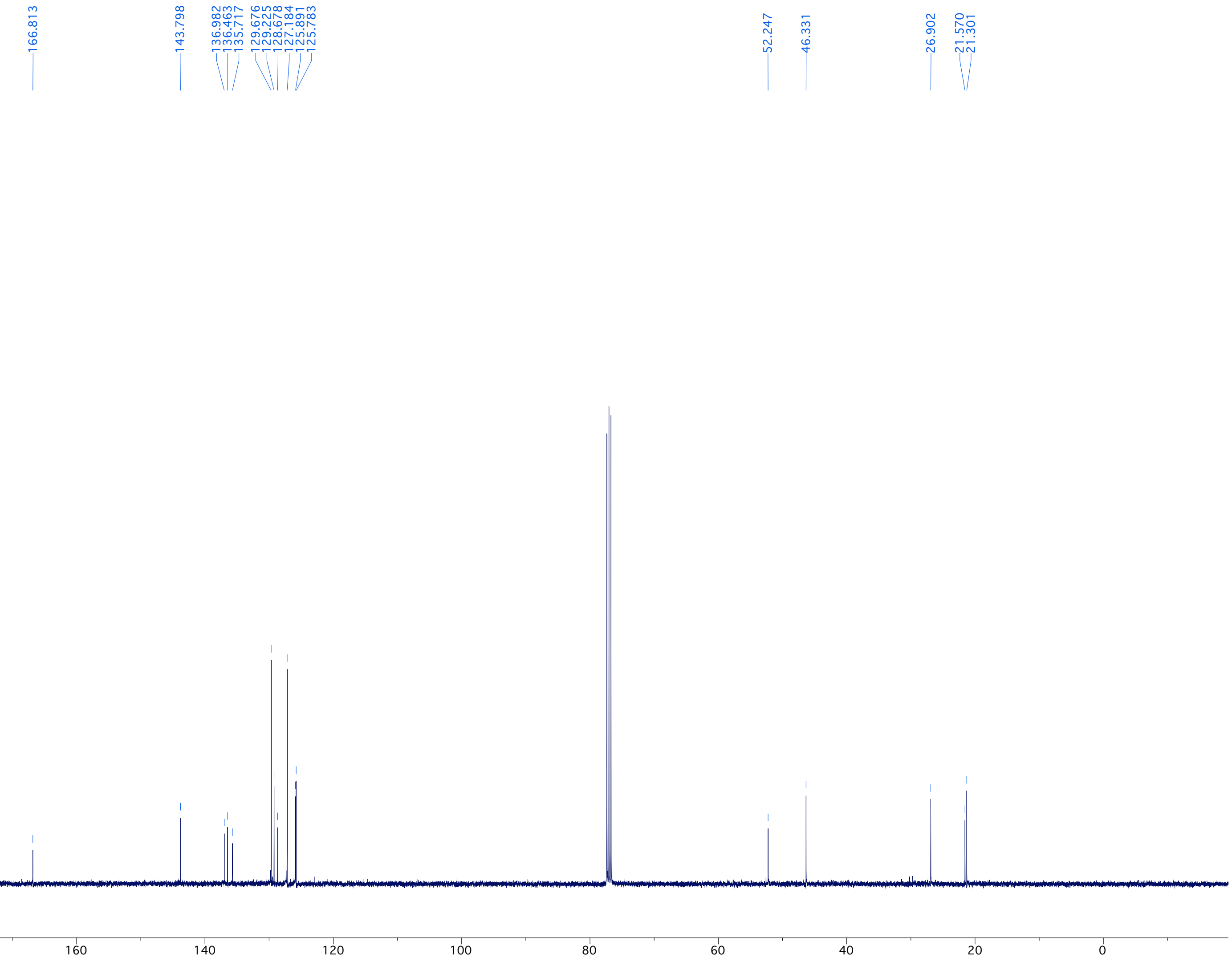

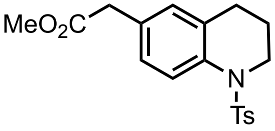

9

^{1}H NMR

400 MHz, $CDCl_3$

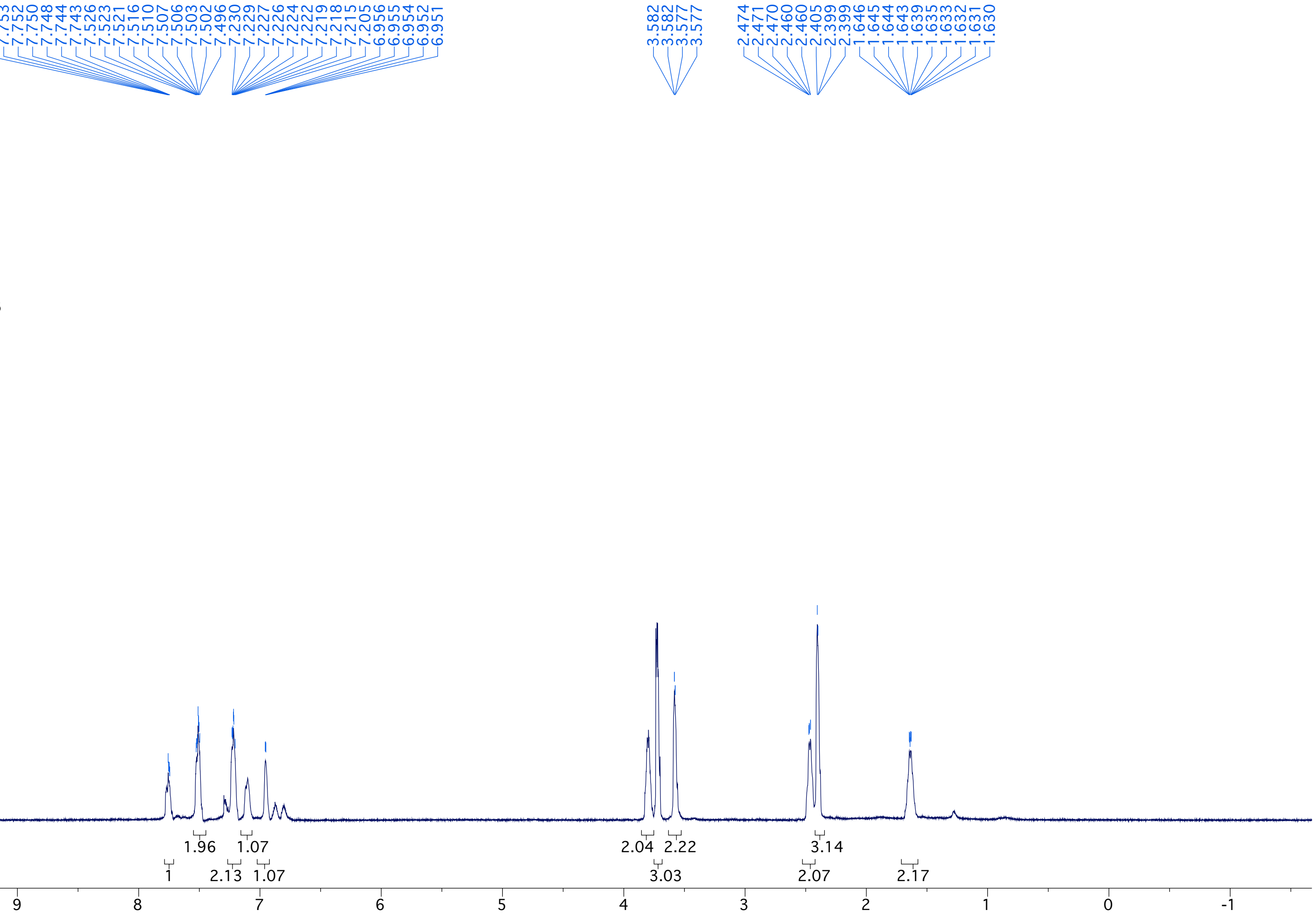

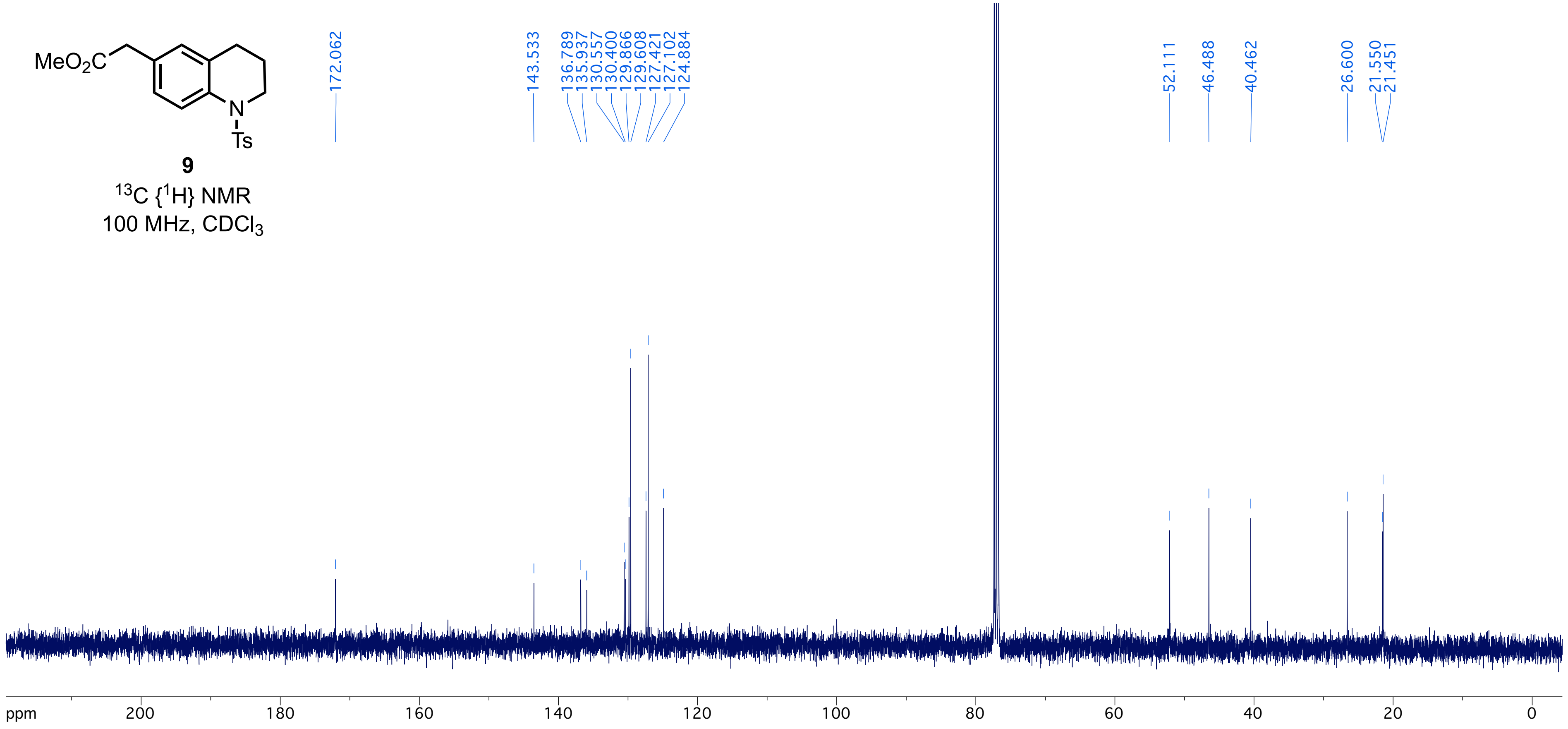

9

$^{13}C\{^{1}H\}$ NMR

100 MHz, $CDCl_3$

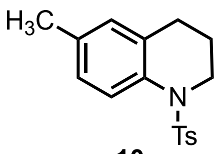

10

^{1}H NMR

400 MHz, $CDCl_3$

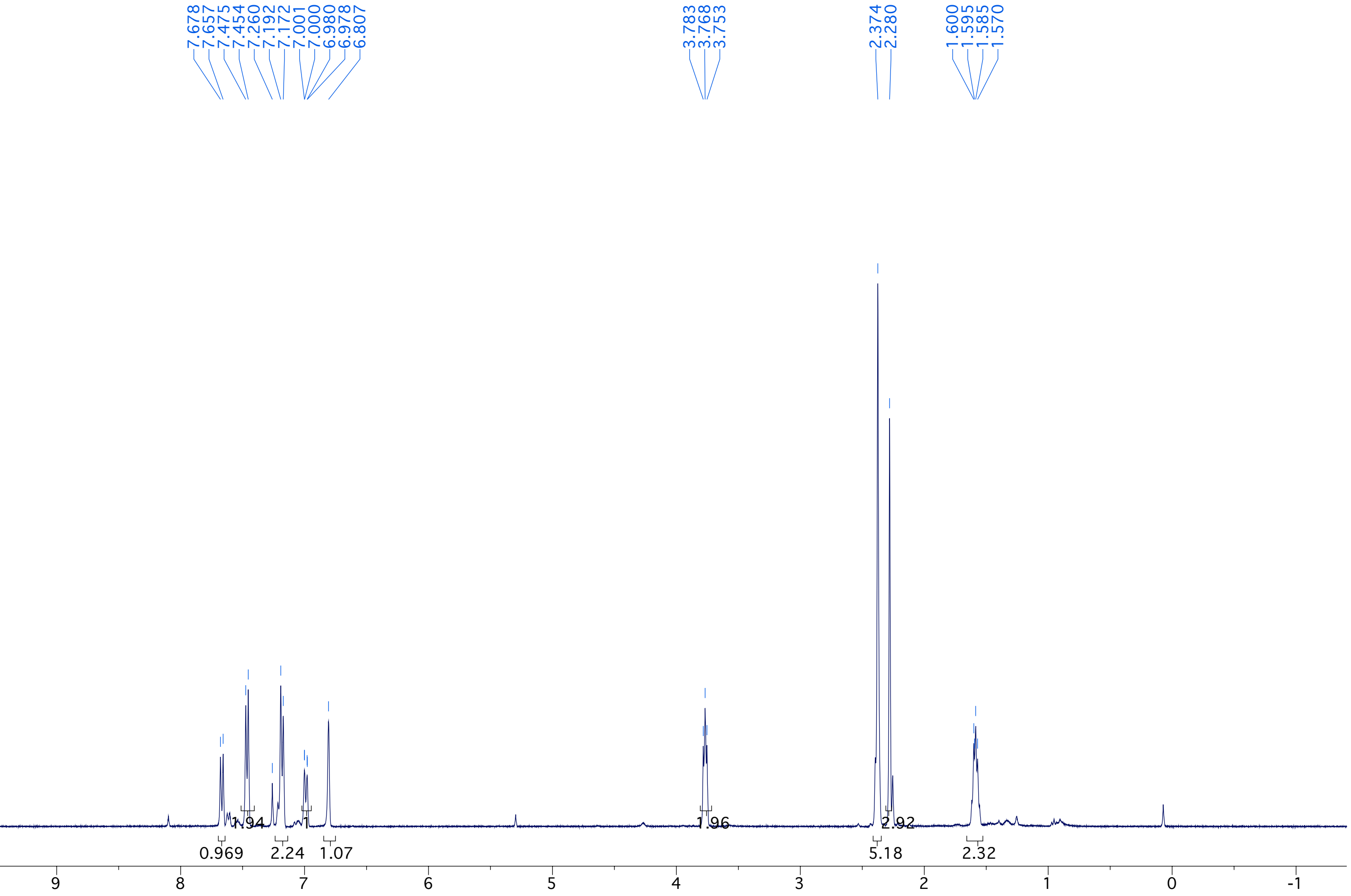

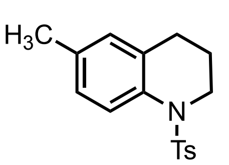

10

$^{13}C\ \{^1H\}$ NMR

100 MHz, $CDCl_3$

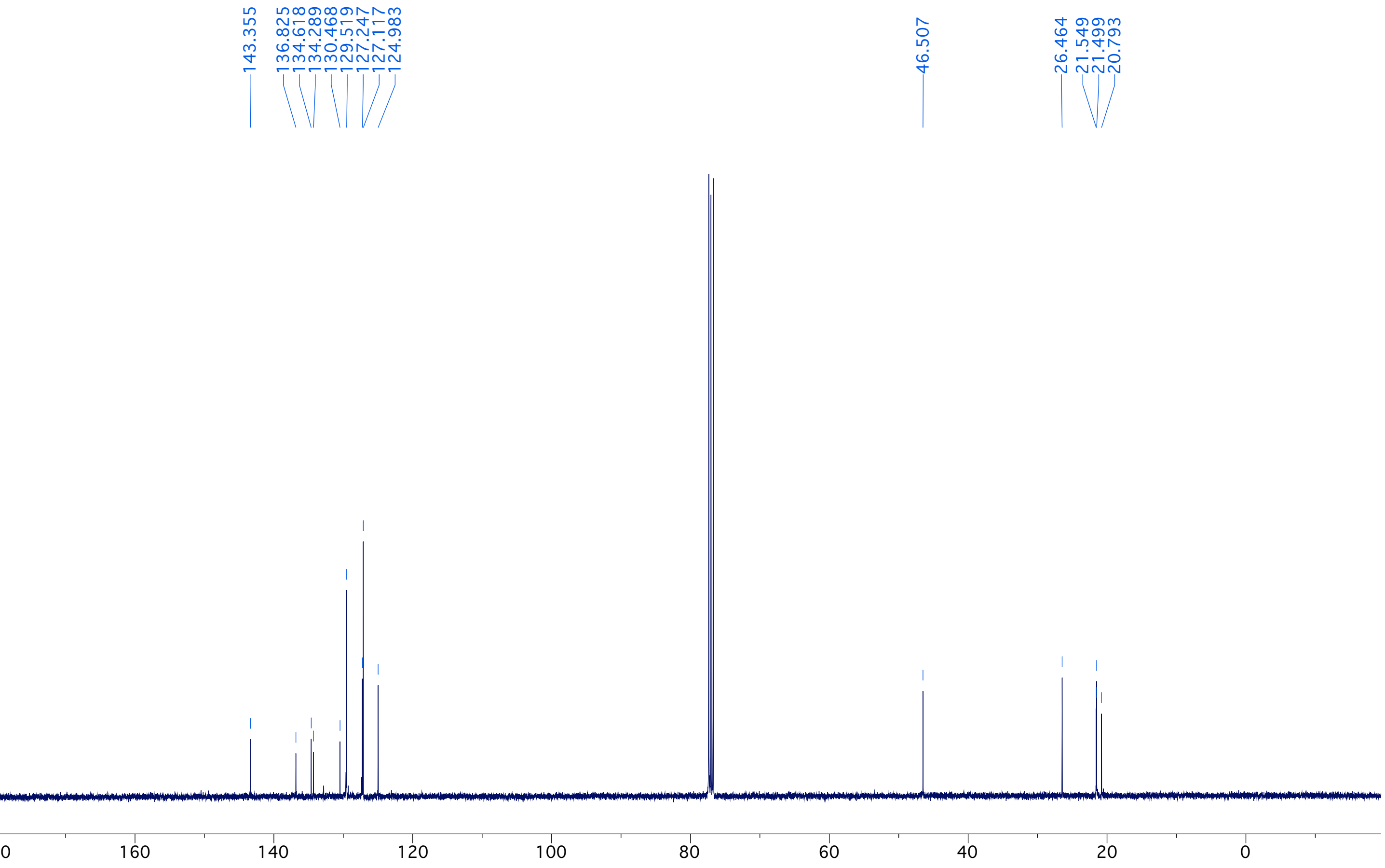

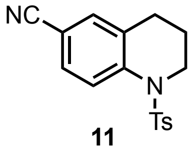

11

^{1}H NMR

400 MHz, $CDCl_3$

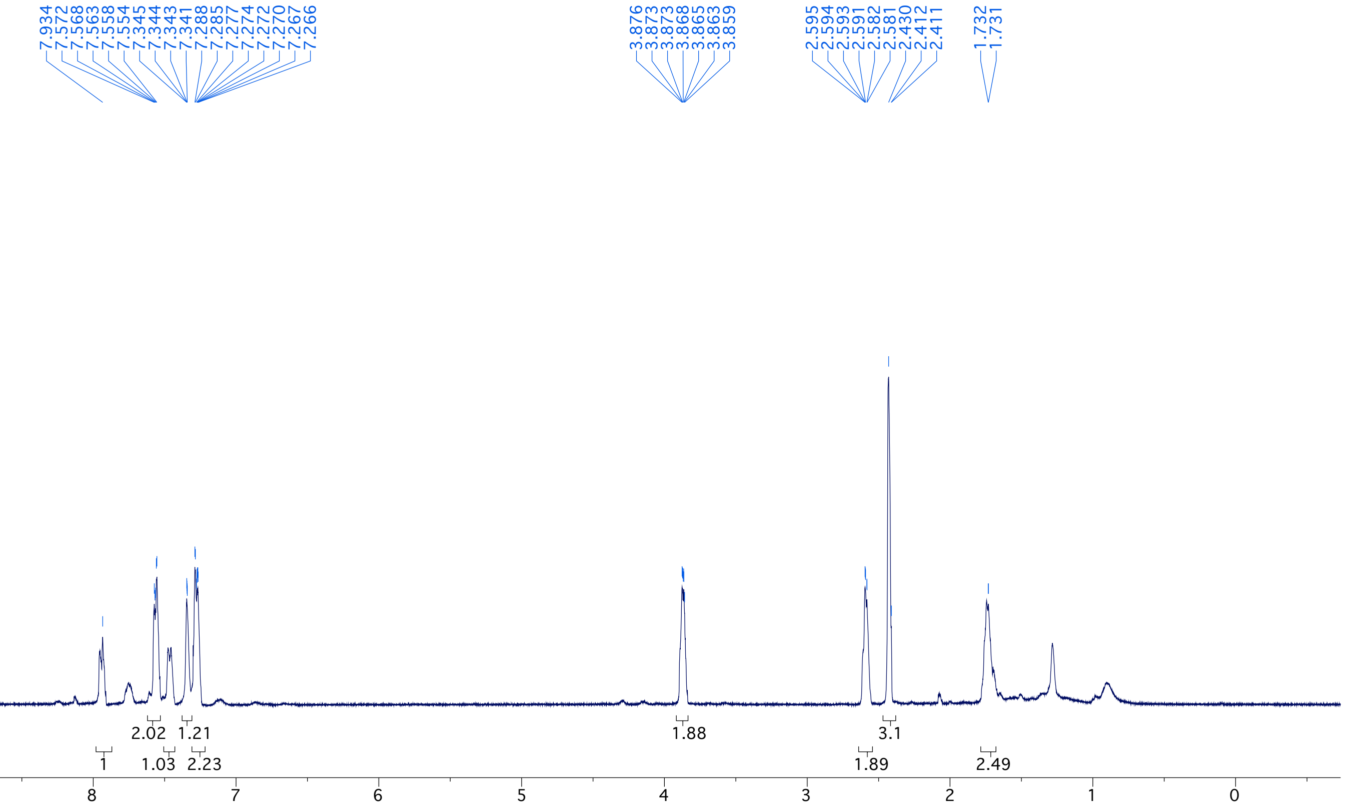

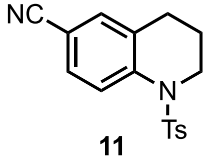

11

$^{13}C\{^{1}H\}$ NMR

100 MHz, $CDCl_3$

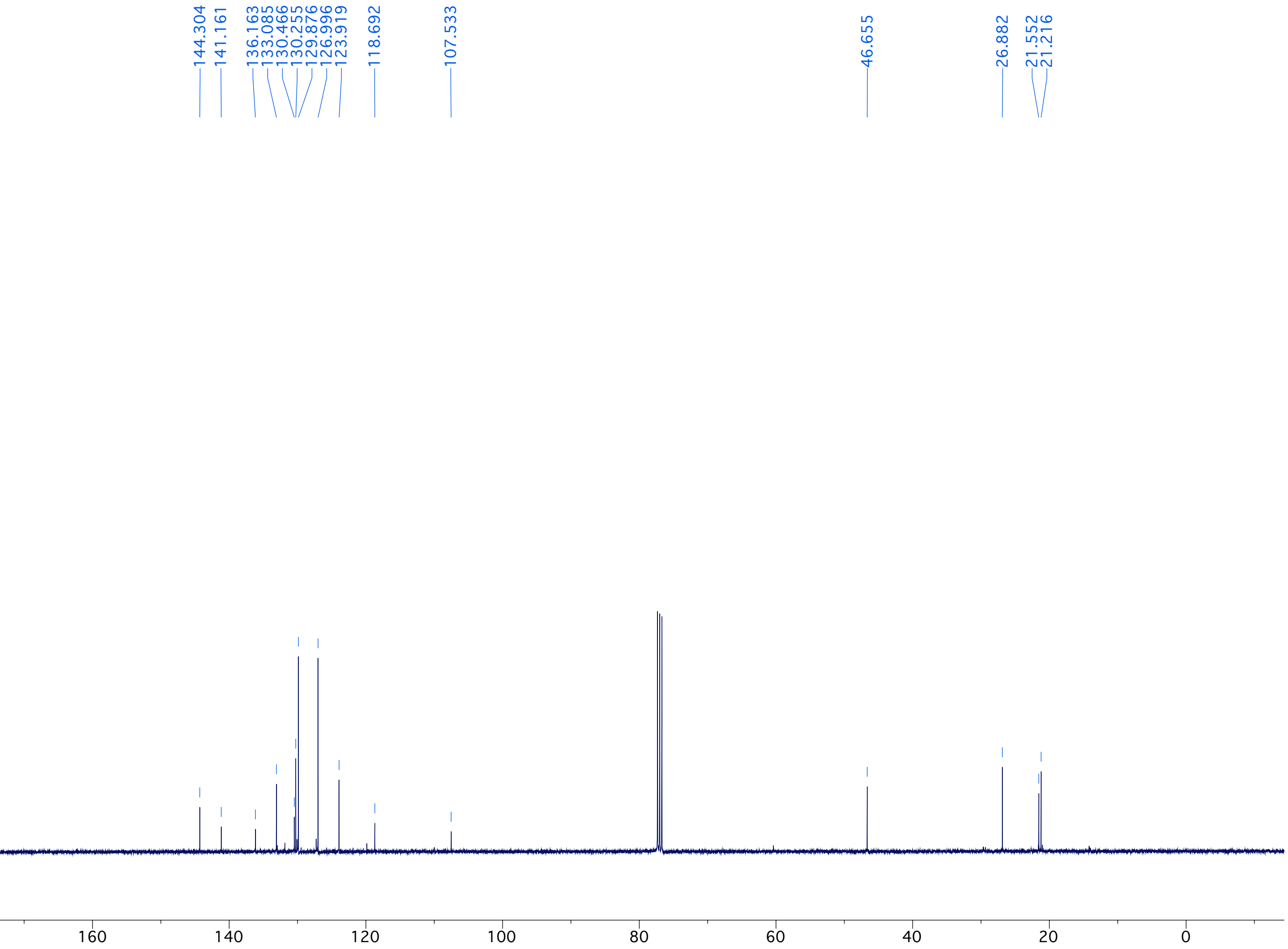

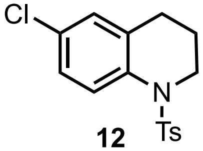

12

^{1}H NMR

400 MHz, $CDCl_3$

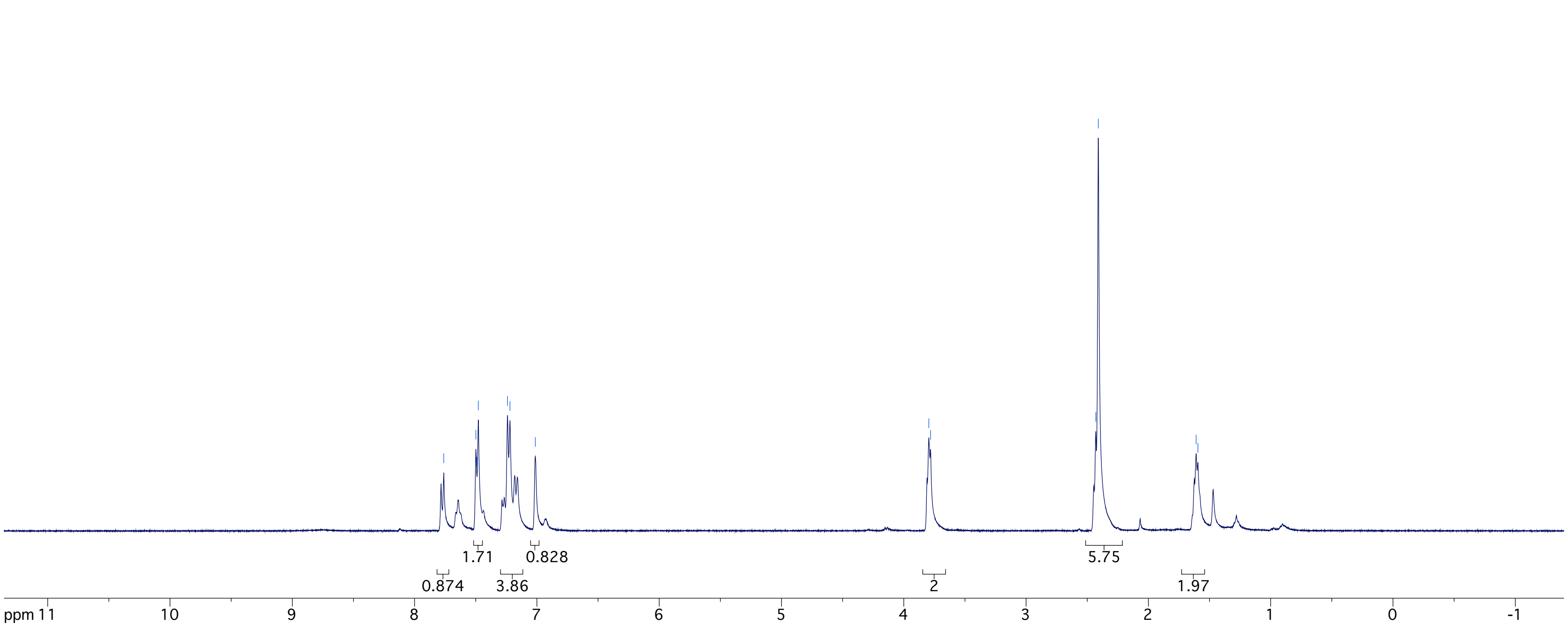

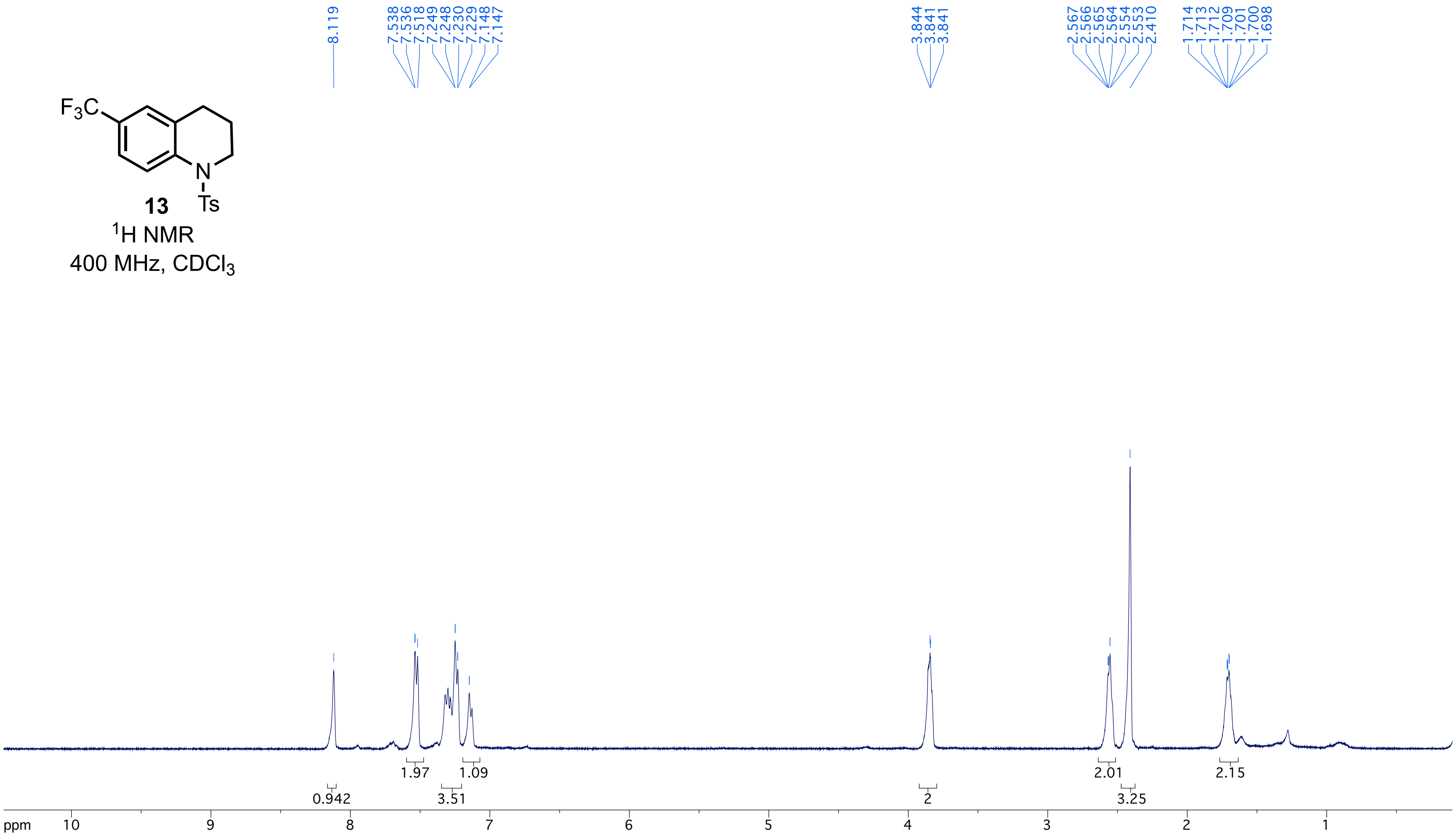
F3C
N
Ts
13
1H NMR
400 MHz, CDCl3
8.119
7.538
7.536
7.518
7.249
7.248
7.230
7.229
7.148
7.147
3.844
3.841
3.841
2.567
2.566
2.565
2.564
2.554
2.553
2.410
1.714
1.713
1.712
1.709
1.701
1.700
1.698
0.942
1.97
3.51
1.09
2
2.01
3.25
2.15
ppm
10
9
8
7
6
5
4
3
2
1

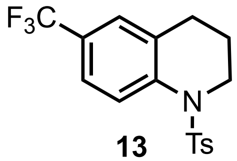

13

$^{19}F\ \{^{1}H\}$ NMR

376 MHz, $CDCl_3$

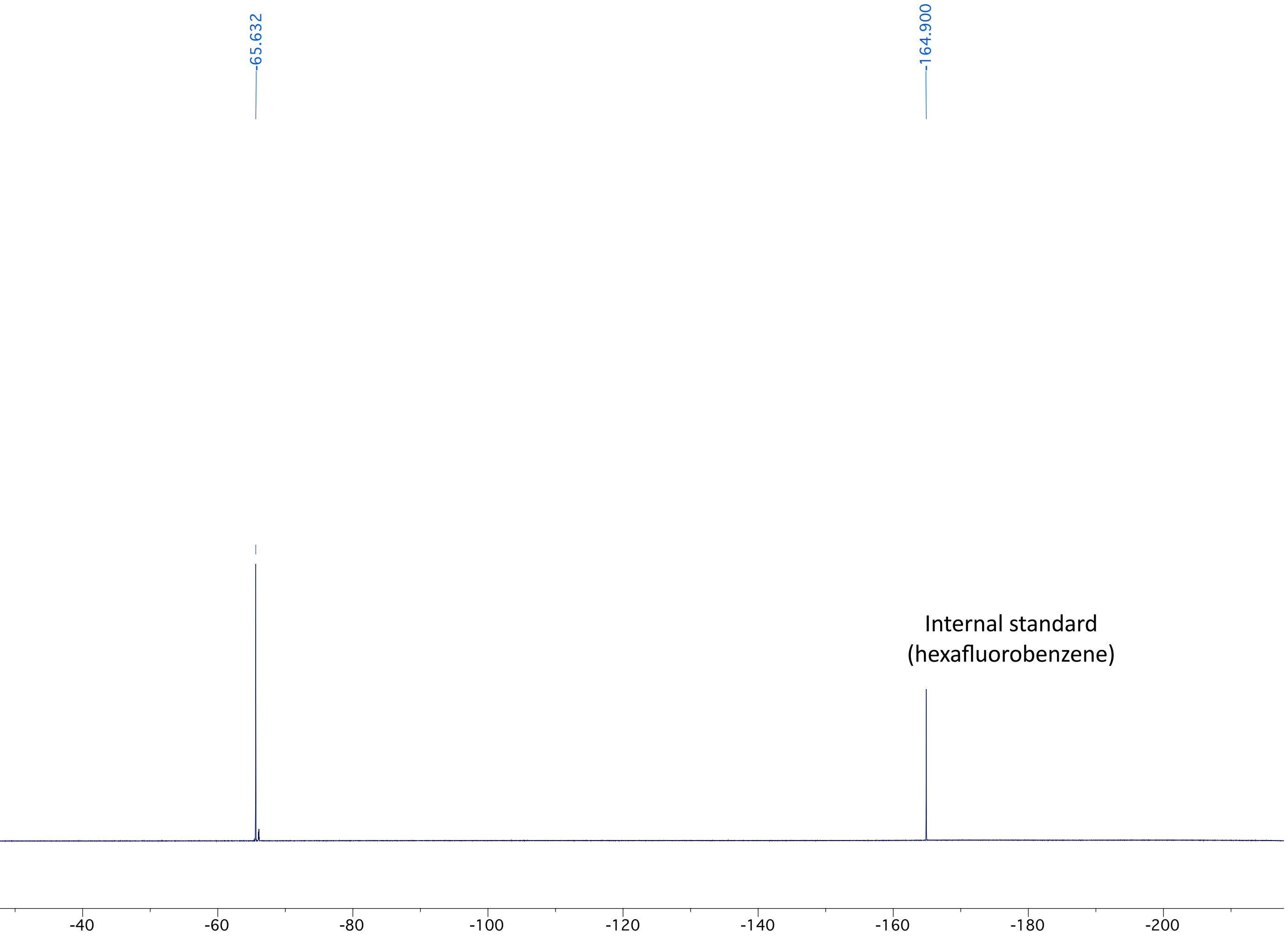

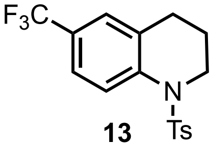

13

^{13}C {^{1}H} NMR
100 MHz, $CDCl_3$

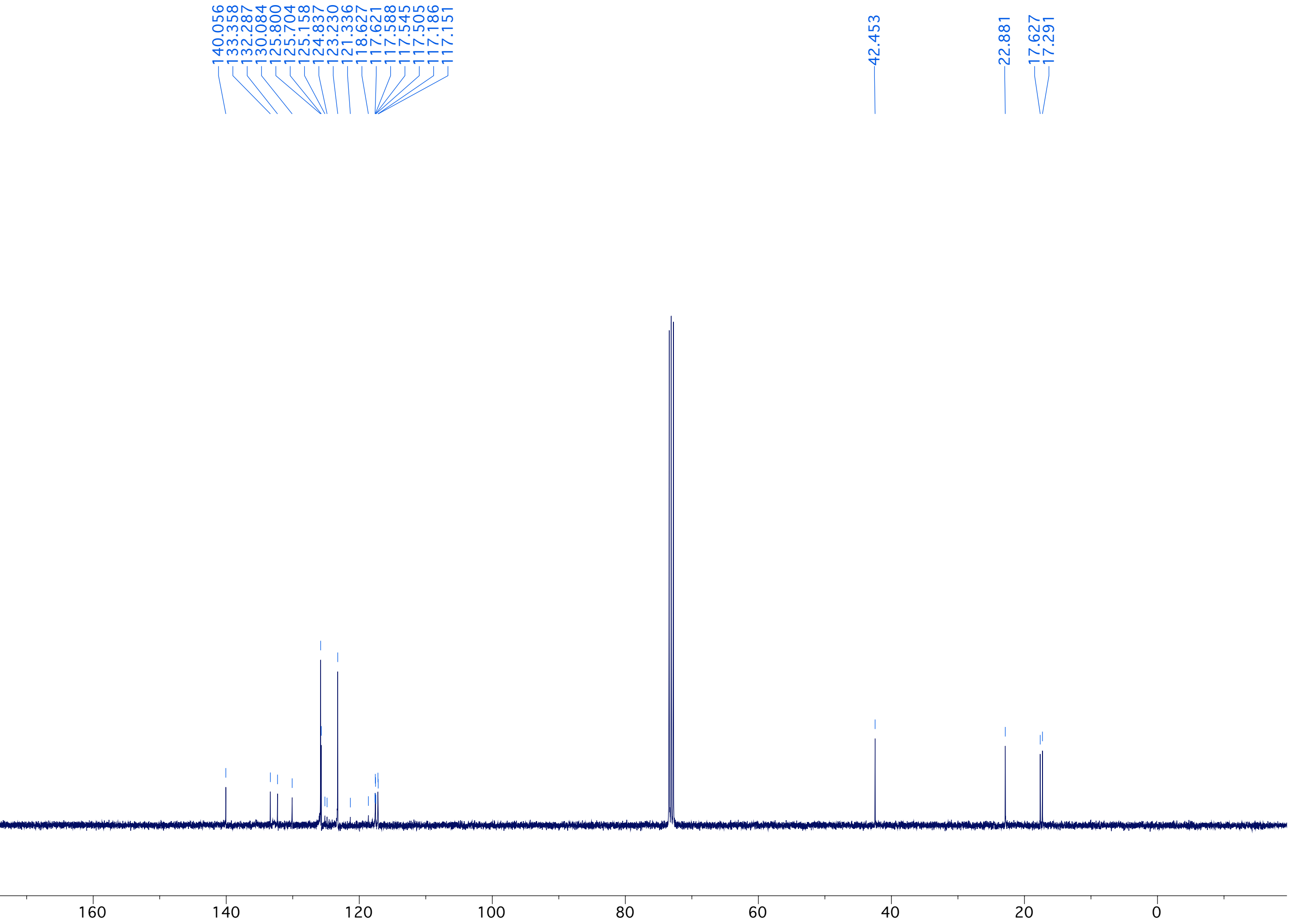

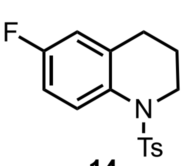

14

^{1}H NMR

400 MHz, $CDCl_3$

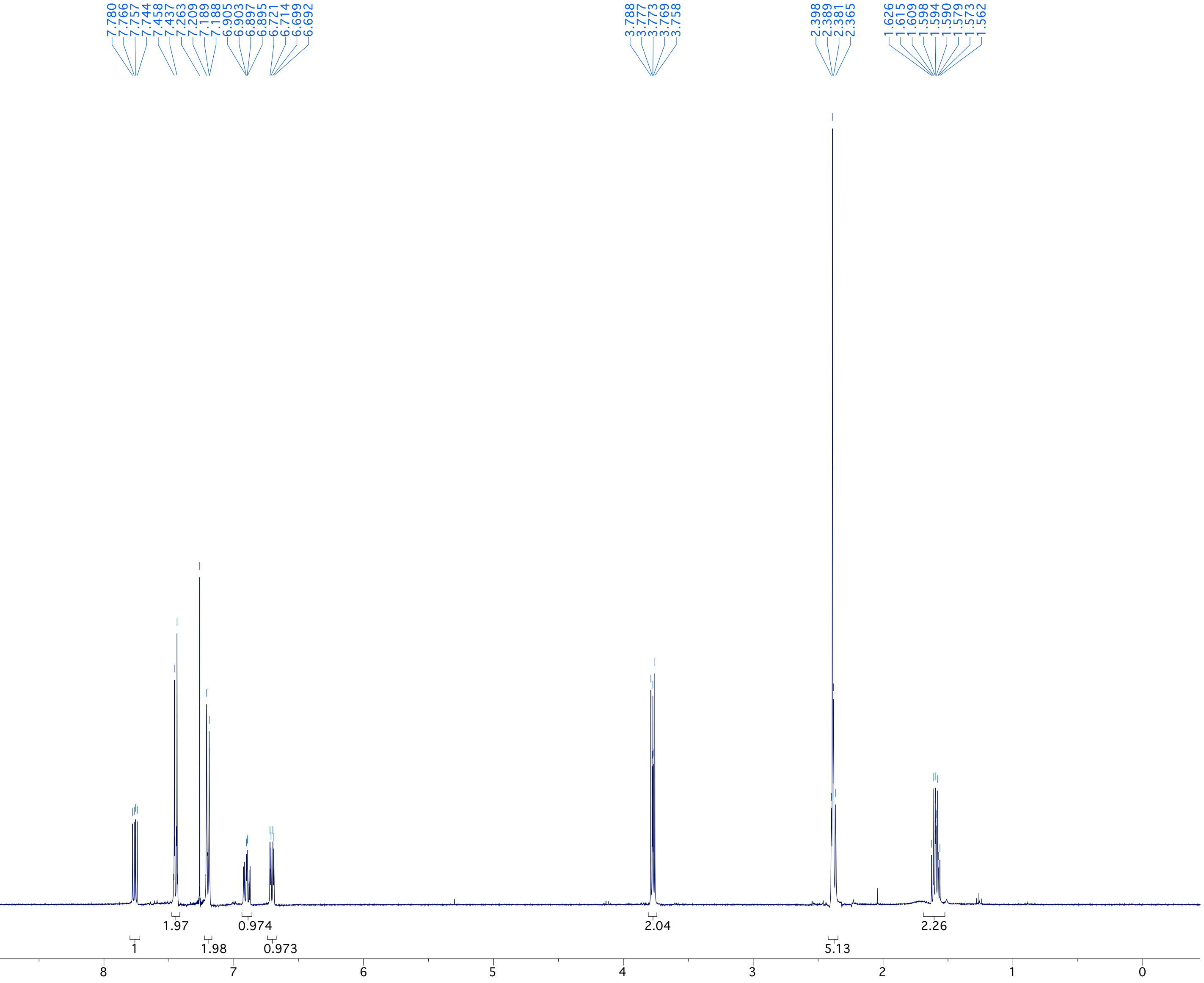

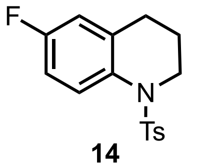

14

$^{19}F\ \{^{1}H\}$ NMR

376 MHz, $CDCl_3$

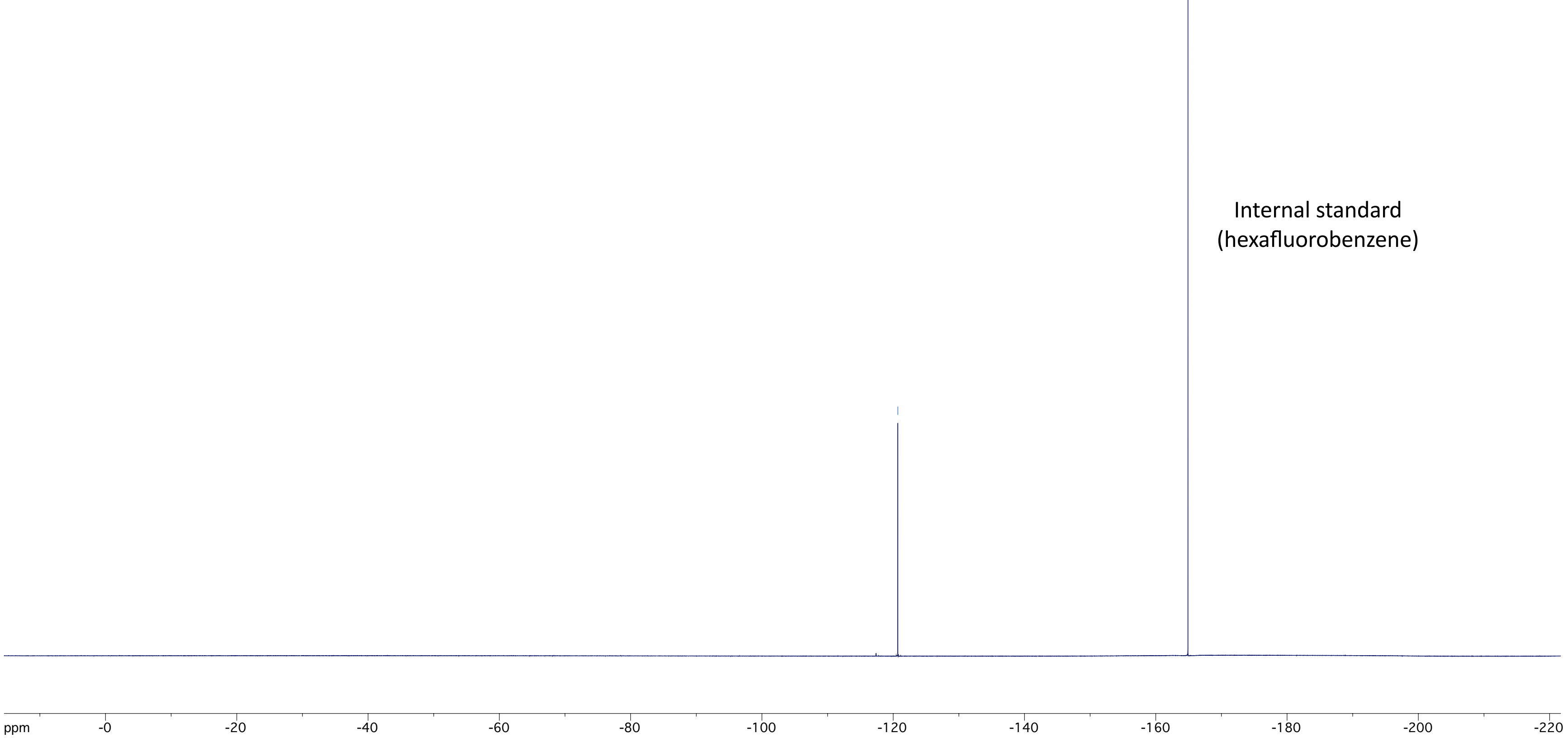

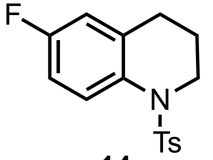

14

$^{13}C\{^1H\}$ NMR

100 MHz, $CDCl_3$

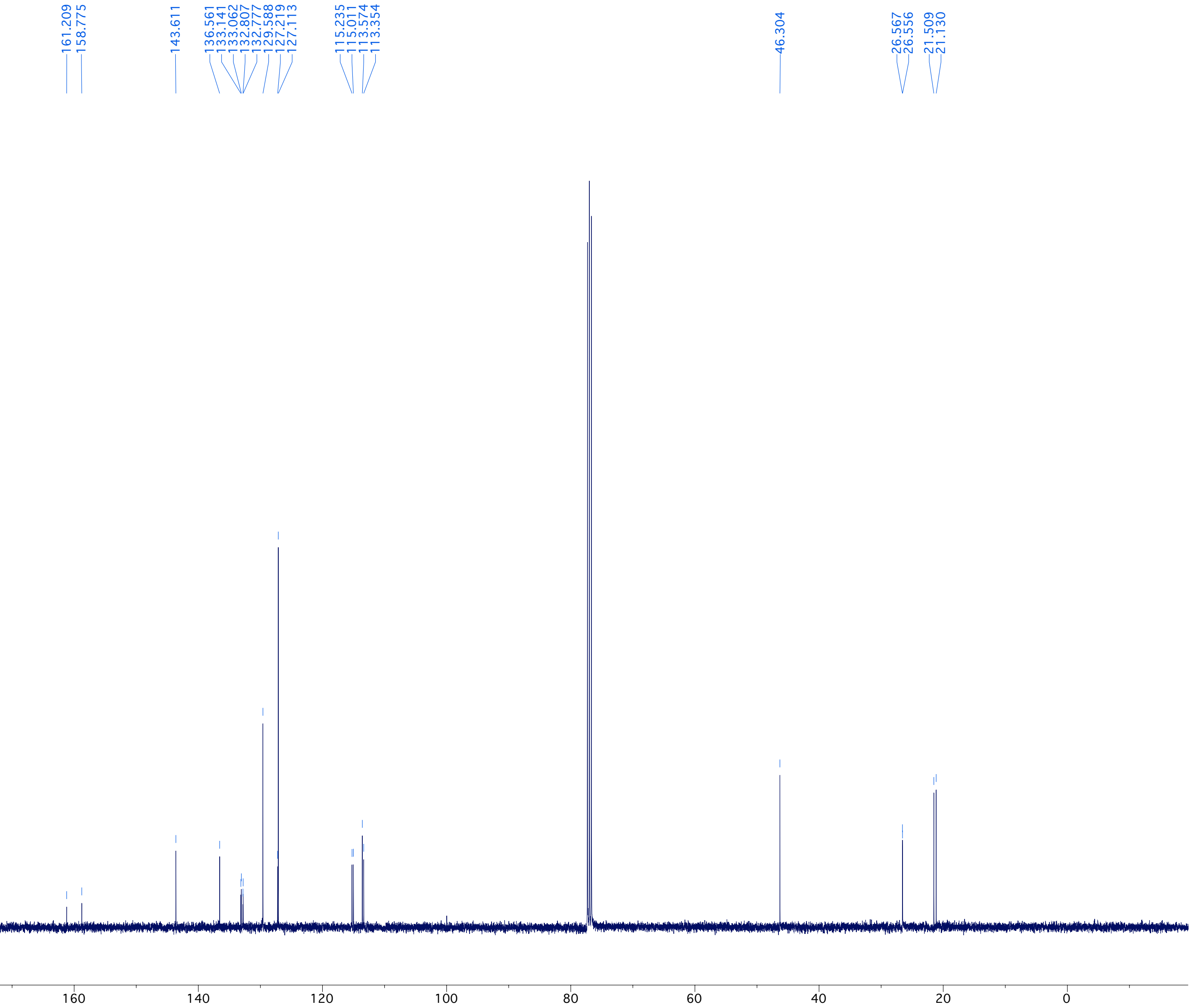

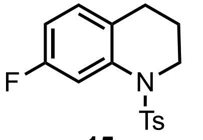

15

^{1}H NMR

400 MHz, $CDCl_3$

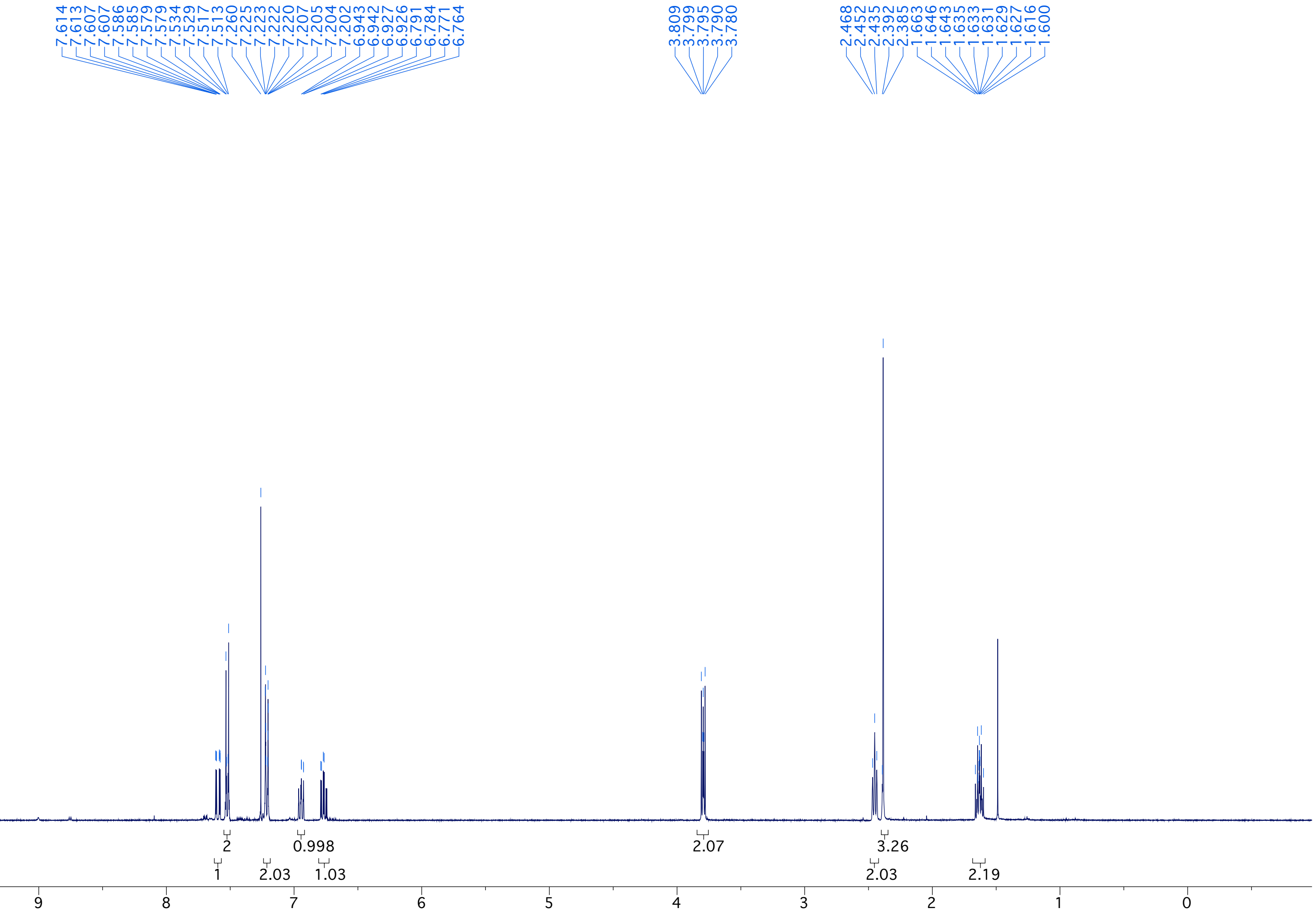

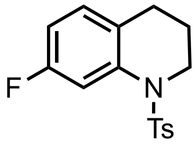

15

^{19}F {^1H} NMR

376 MHz, $CDCl_3$

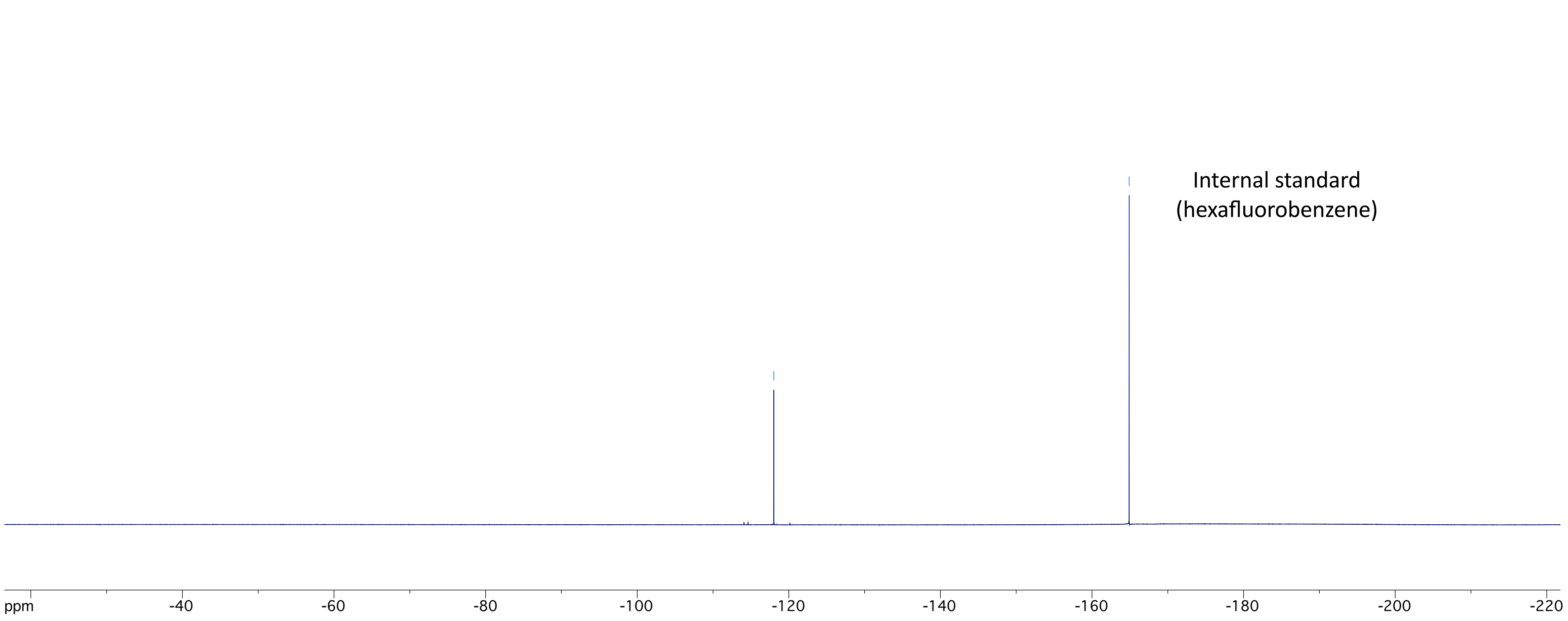

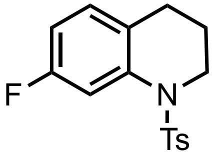

15

$^{13}C\ \{^{1}H\}$ NMR

100 MHz, $CDCl_3$

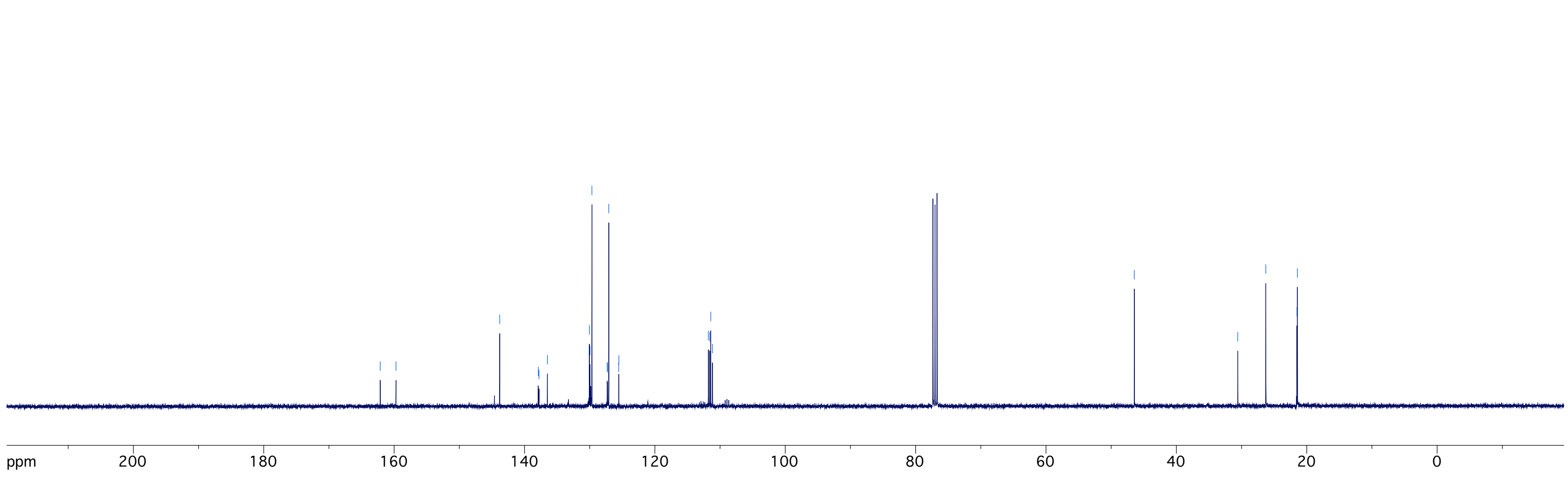

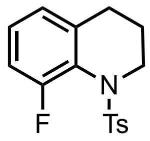

16

^1H NMR

400 MHz, $CDCl_3$

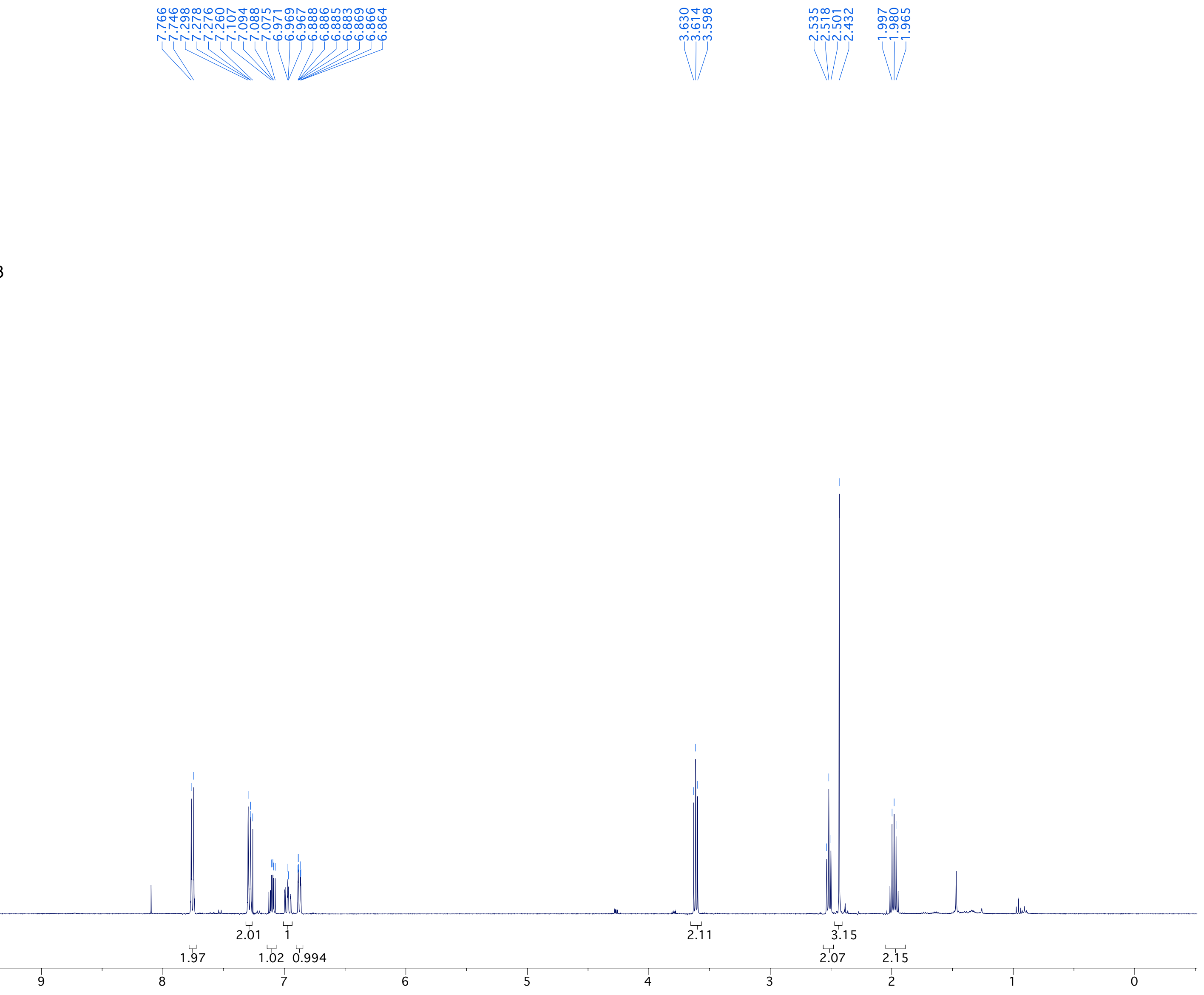

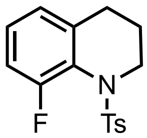

16

$^{13}C\ \{^1H\}$ NMR

100 MHz, $CDCl_3$

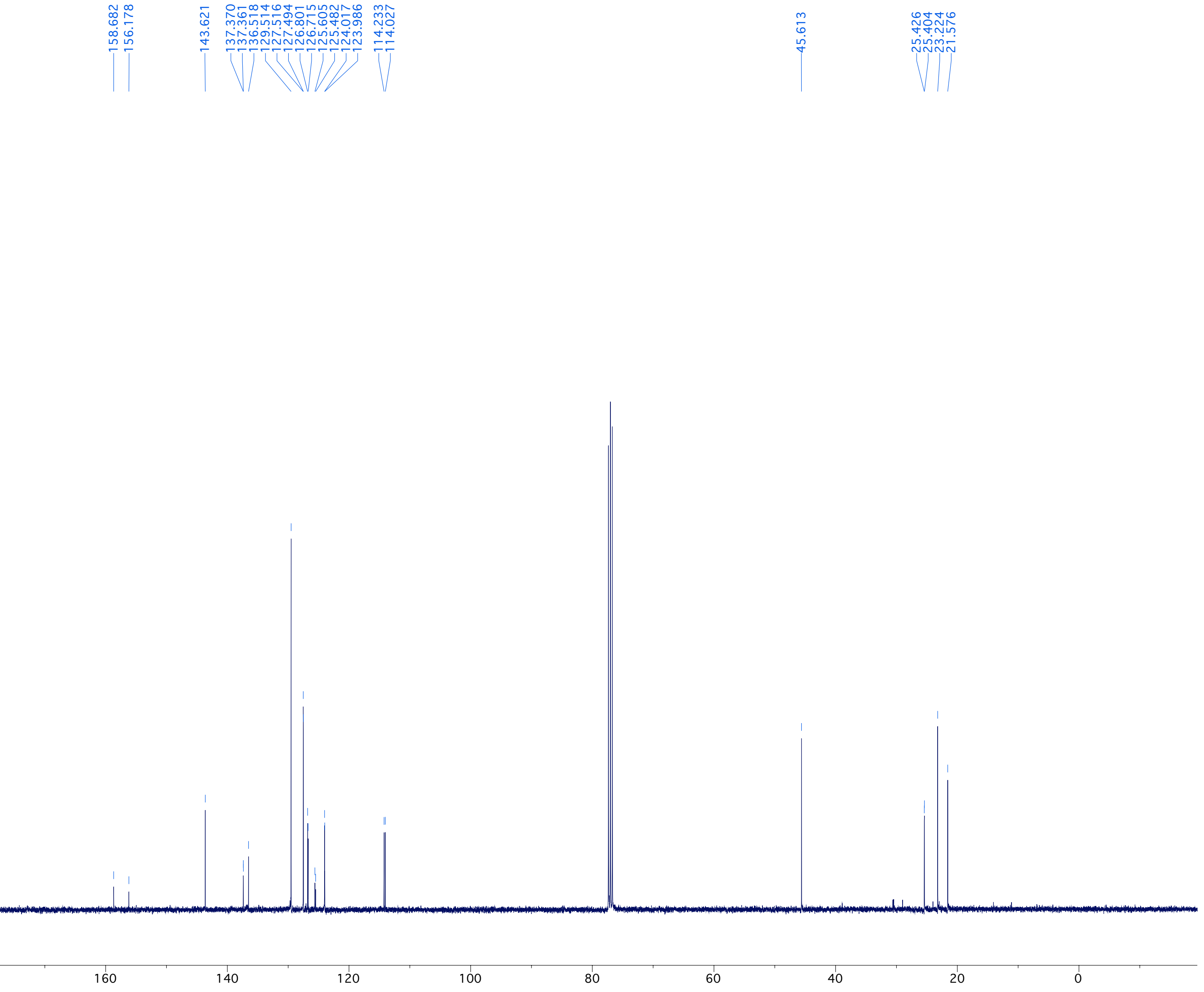

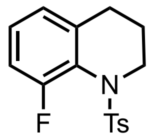

16

^{19}F {^{1}H} NMR

376 MHz, $CDCl_3$

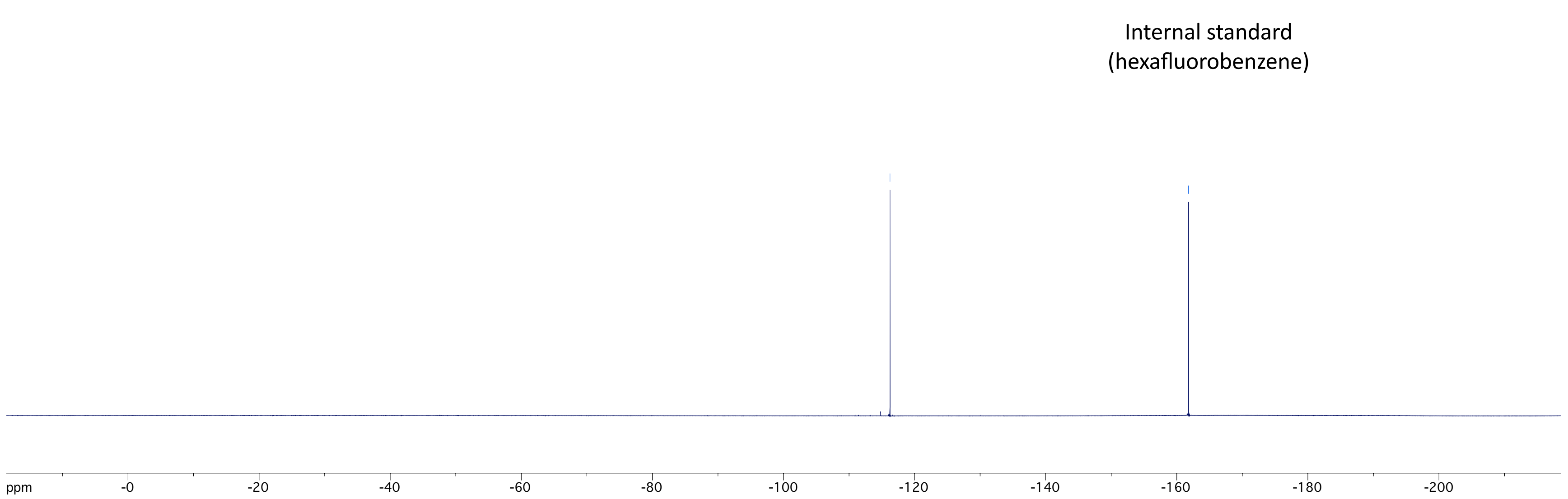

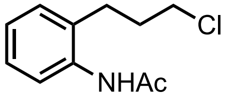

18

^1H NMR

400 MHz, $CDCl_3$

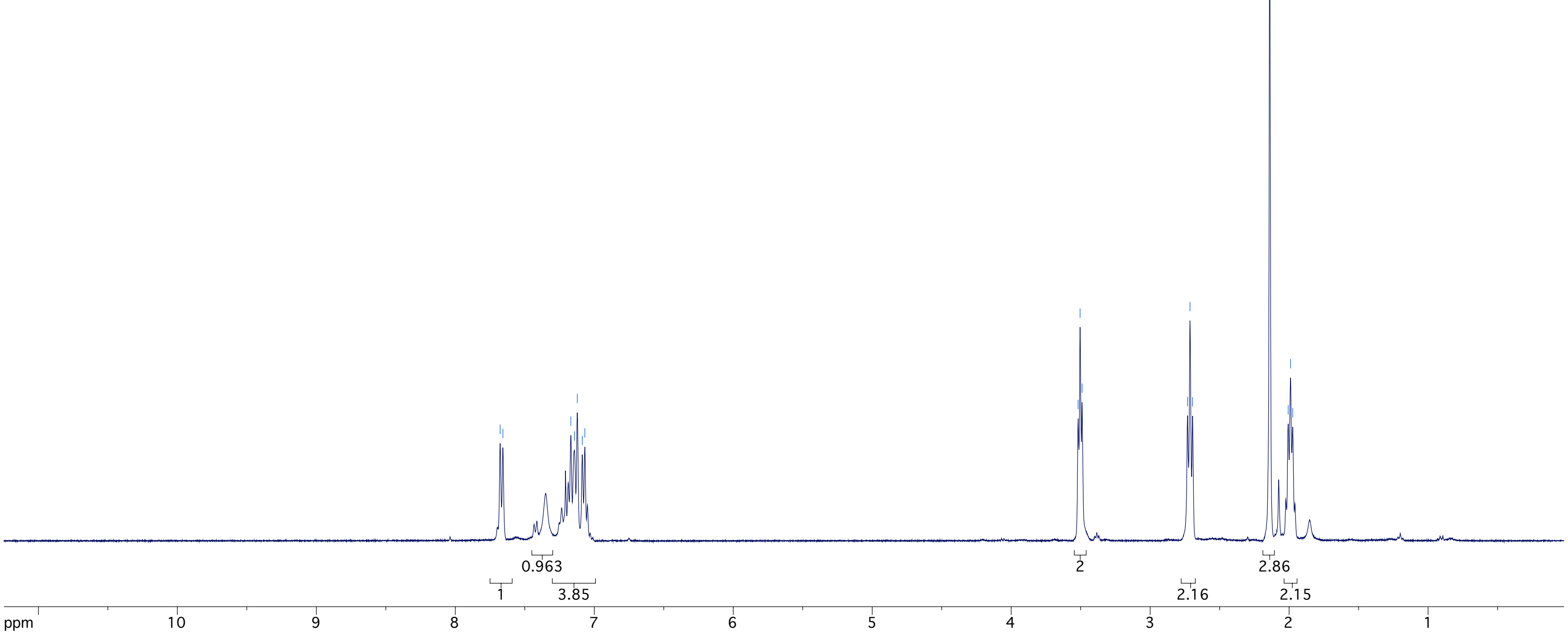

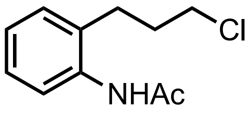

18

$^{13}C\ \{^{1}H\}$ NMR

100 MHz, $CDCl_3$

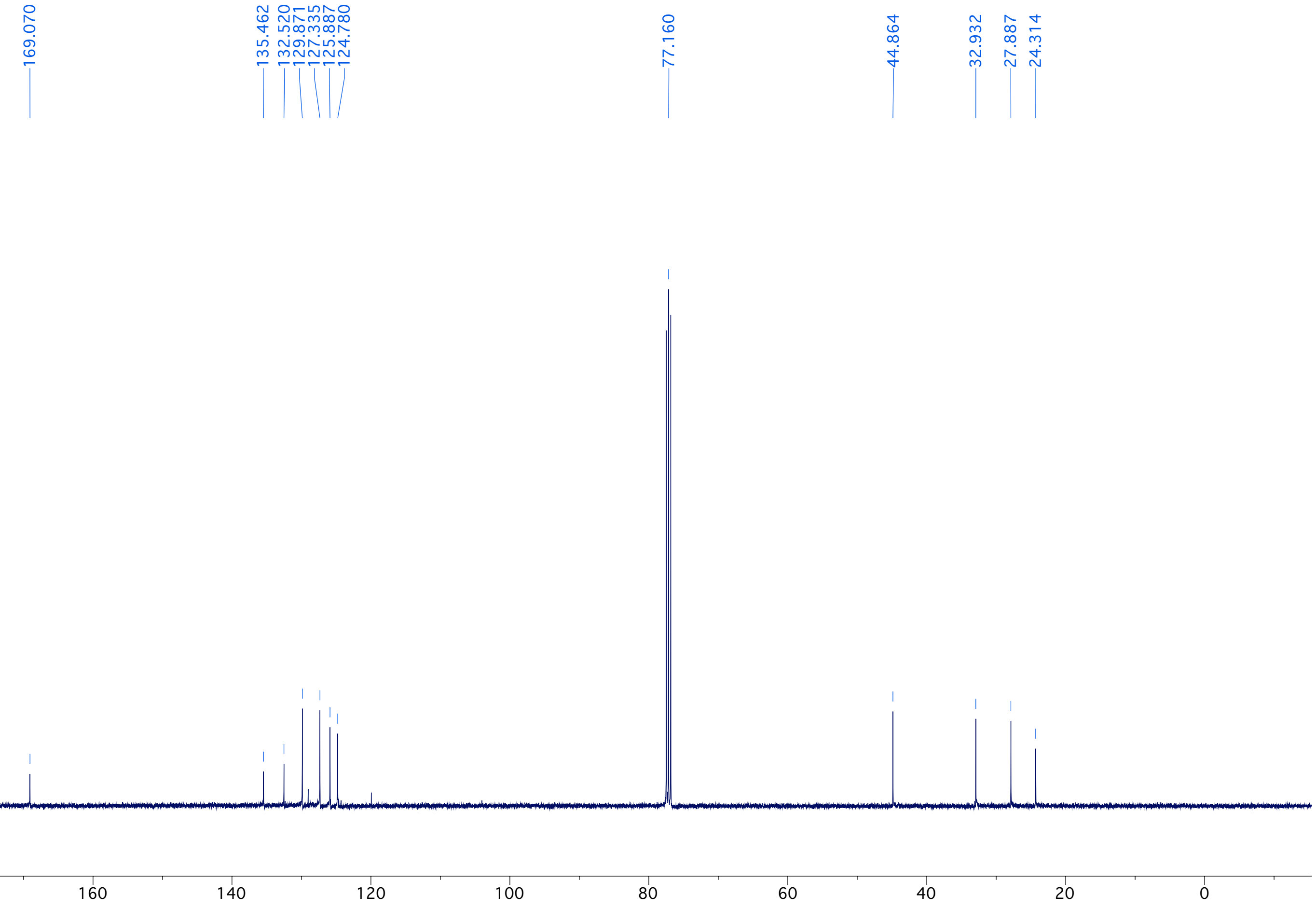

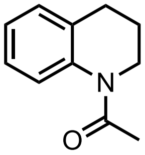

19
^{1}H NMR
400 MHz, $CDCl_3$

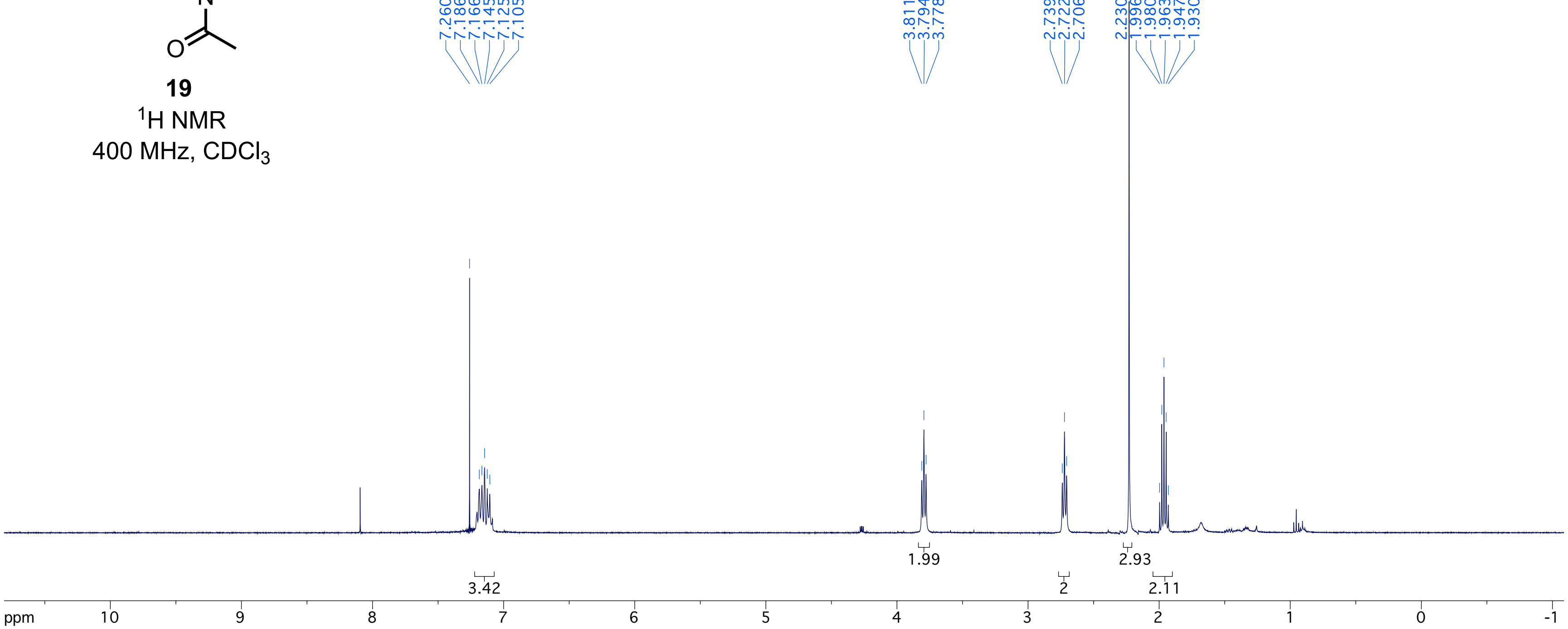

Supplement: Supplementary file 1 — jo3c02267_si_001.pdf [file jo3c02267_si_001.pdf]
